# Identification of CB1 Ligands among Drugs, Phytochemicals and Natural-Like Compounds: Virtual Screening and In Vitro Verification

**DOI:** 10.1021/acschemneuro.2c00502

**Published:** 2022-10-05

**Authors:** Adam Stasiulewicz, Anna Lesniak, Piotr Setny, Magdalena Bujalska-Zadrożny, Joanna I. Sulkowska

**Affiliations:** 1 Department of Drug Chemistry, Faculty of Pharmacy, Medical University of Warsaw, Banacha 1, 02-097 Warsaw, Poland; 2 Centre of New Technologies, University of Warsaw, Banacha 2c, 02-097 Warsaw, Poland; 3 Department of Pharmacodynamics, Faculty of Pharmacy, Medical University of Warsaw, Banacha 1, 02-097 Warsaw, Poland

**Keywords:** cannabinoid receptor, phytocannabinoids, travoprost, ginkgetin, docking, QSAR

## Abstract

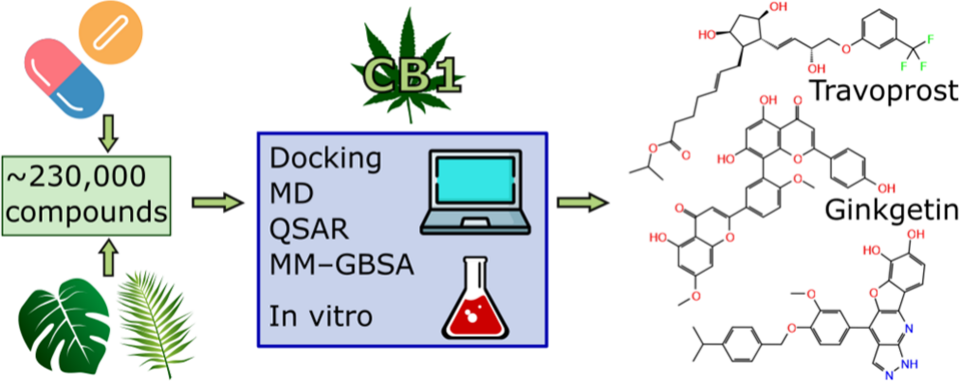

Cannabinoid receptor type 1 (CB1) is an important modulator of many key physiological functions and thus a compelling molecular target. However, safe CB1 targeting is a non-trivial task. In recent years, there has been a surge of data indicating that drugs successfully used in the clinic for years (e.g. paracetamol) show CB1 activity. Moreover, there is a lot of promise in finding CB1 ligands in plants other than *Cannabis sativa*. In this study, we searched for possible CB1 activity among already existing drugs, their metabolites, phytochemicals, and natural-like molecules. We conducted two iterations of virtual screening, verifying the results with in vitro binding and functional assays. The in silico procedure consisted of a wide range of structure- and ligand-based methods, including docking, molecular dynamics, and quantitative structure–activity relationship (QSAR). As a result, we identified travoprost and ginkgetin as CB1 ligands, which provides a starting point for future research on the impact of their metabolites or preparations on the endocannabinoid system. Moreover, we found five natural-like compounds with submicromolar or low micromolar affinity to CB1, including one mixed partial agonist/antagonist viable for hit-to-lead phase. Finally, the computational procedure established in this work will be of use for future screening campaigns for novel CB1 ligands.

## 1 Introduction

The endocannabinoid system (ECS) is one of the most important regulatory systems in the human organism. It is responsible for the control of a vast array of physiological processes and functions, including nociception, mood regulation, appetite, fat and glucose metabolism, neurogenesis and neurodegeneration, cell proliferation and many others.^1^ The most important protein of the ECS, cannabinoid receptor type 1 (CB1), is a well-established molecular target. Compounds acting via this receptor are present in approved drugs, clinical trial candidates or are under consideration at various preclinical levels.^2^

CB1 ligands with different intrinsic activities are valuable for various therapeutic purposes. CB1 agonists or partial agonists are useful among others as analgetics^3^ and anti-emetic agents.^4^ CB1 antagonists/inverse agonists may act as anorectics^5^ or antifibrotic agents.^6^ However, effective and safe CB1 targeting is a non-trivial task, as the ECS is a multi-purpose system.^2^ Although CB1 agonists, including phytocannabinoids, are generally well tolerated,^7^ their pharmacotherapeutic profile is not perfect. For example, long-term use of *Cannabis sativa* may come with a risk of cognitive impairment.^8^ On the other hand, CB1 antagonists/inverse agonists usage may cause serious psychiatric disorders, such as anxiety or depression.^5^

To date, multiple ways to overcome the aforementioned obstacles have been proposed. Most of the adverse effects related to the modulation of CB1 activity are caused by the subpopulation of this receptor localized in the central nervous system.^9^ Thus, one of the possible solutions is to design peripheral CB1 ligands.^10^ Another prominent strategy is to utilize CB1 neutral antagonists instead of inverse agonists.^11^ CB1 allosteric modulation may be a promising direction as well.^12^

It is often not realized that the most successful compounds acting via CB1 in a safe manner are among already known drugs. Paracetamol (acetaminophen), one of the most often used active substance all over the world, was proved to owe its analgesic activity in major part to anandamide reuptake inhibition and CB1 activation by its active metabolite—*N*-arachidonoylphenolamine (AM404).^13,14^ Other notable examples of pharmacologically relevant substances acting via CB1 or interacting with this receptor include metamizole (dipyrone),^15^ fenofibrate^16^ and raloxifene.^17^ Is is possible that there may be other active drug ingredients with considerable affinity toward CB1. If so, recognizing their mechanism of action may lead to more rational and thus safer use of those substances in pharmacotherapy. In addition, rational repurposing of drugs with known safety profiles may be implemented.

One of the most successful strategies of targeting the ECS is based on *Cannabis sativa* or its specific phytocannabinoids. While they have proved effective in many indications^18,19^ and are generally well tolerated, the therapy comes with a risk of addiction and adverse effects, such as memory impairment or cognitive dysfunctions caused by the long-term use.^8^ Thus, there is still room for improvement in this therapeutic area. Indeed, in recent years there have been numerous attempts to find phytocannabinoids in other plants.^20^ Alas, so far the results are not satisfactory enough, as the revealed compounds have only low or moderate affinities toward CB1, unsatisfactory intrinsic activity and their pharmacological relevance has not been fully determined.^20^ Therefore, there is a huge potential in finding new non-*Cannabis* phytocannabinoids with a more favorable pharmacological profile.

**Figure 1:**
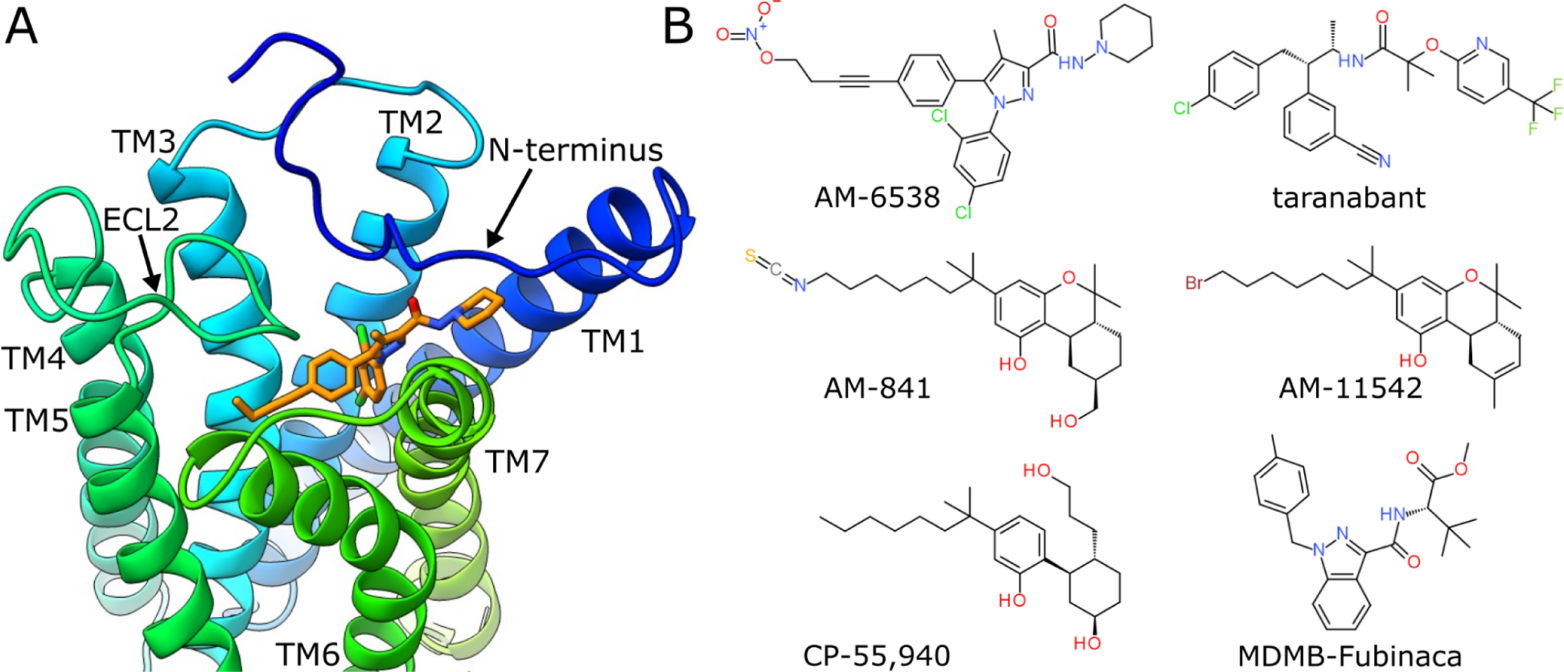
(A) CB1 binding site with bound AM-6538. Based on PDB ID: 5TGZ. (B) Structural formulas of six ligands present in CB1 structures deposited in the PDB. ECL2, extracellular loop 2; TM, transmembrane helix.

Because of its importance in the human organism and possible medical applications, CB1 has been a subject of great scientific interest. Many drug design projects, aimed at this crucial molecular target, were hindered by insufficient knowledge of its structure. In 2016, Hua et al.^21^ crystallized CB1 and elucidated its tertiary structure (Figure 1A). This allowed for better understanding of CB1 action and created a possibility for rational drug design. As of today, there are eight CB1 structures deposited in the Protein Data Bank (PDB)^21−27^ (Table 1). They bind six different ligands (Figure 1B) from four chemotypes and in some cases are complexed with G-protein or allosteric modulators. Accordingly, they represent diverse binding site conformations, providing a suitable ground for structure-based (SB) design or screening. Moreover, there are over 4000 records on *K*_i_ values measured for CB1 ligands in the ChEMBL database,^28,29^ which correspond to over 2500 unique compounds with known binding affinities. Such quantity allows for rational utilization of ligand-based (LB) methods.

**Table 1:** CB1 Structures Deposited in the PDB^a^

PDB ID	ligand	intrinsic activity	additional features	method	resolution (Å)	references
5TGZ	AM-6538	antagonist		XRD	2.80	(21)
5U09	taranabant	inverse agonist		XRD	2.60	(22)
5XR8	AM-841	agonist		XRD	2.95	(23)
5XRA	AM-11542	agonist		XRD	2.80	(23)
6N4B	MDMB-Fubinaca	agonist	G-protein	Cryo-EM	3.00	(24)
6KPG	AM-841	agonist	G-protein	Cryo-EM	3.00	(26)
6KQI	CP-55,940	agonist	ORG27569 (NAM)	XRD	3.25	(25)
7V3Z	CP-55,940	agonist		XRD	3.29	(27)

a Cryo-EM, cryoelectron microscopy; NAM, negative allosteric modulator; XRD, X-ray diffraction.

The aim of this study was to identify novel CB1 ligands among drugs, drug metabolites, phytochemicals, and related compounds. The elucidation of CB1 structure provided an excellent opportunity to screen a large number of compounds in a short time, using in silico methods. In this study, we utilized a wide range of computational techniques, combining both SB and LB approaches and evaluated the results of our predictions with in vitro binding and functional assays. As a result, we identified a drug active ingredient and a phytochemical compound as new CB1 ligands. Also, we found several hits among natural-like compounds, one of them being a suitable candidate for hit-to-lead stage. Moreover, the in silico procedure established in this study could be utilized in other future projects focusing on search for novel CB1 ligands.

## 2 Results and Discussion

### 2.1 Double Iterative Screening

In order to find CB1 ligands among drugs, drug metabolites, and natural compounds, we conducted iterative screening that consisted of two major parts. The first iteration included initial virtual screening (VS) of small ligand libraries (∼10,000 compounds total), with the focus on SB methods. This strategy was adopted to test whether SB techniques alone would be suitable for such non-trivial, hydrophobic molecular targets as CB1 (Figure S1) and to possibly increase chances for the identification of new CB1 ligands with considerably distinct chemotypes. The best candidates were evaluated in a cell-based displacement binding assay. The second iteration was planned based on the conclusions derived from the first one. Herein, we decided to combine LB and SB methods. This time, we screened a more vast chemical space of ∼230,000 compounds, which also included the previously mentioned 10,000. The top scored molecules were verified in the in vitro binding assay.

#### 2.1.1 First Iteration

The first iteration relied heavily on SB methods, mainly docking and molecular dynamics (MD). From the four CB1 structures available at the time (PDB IDs: 5TGZ, 5U09, 5XR8, and 5XRA), we selected one active CB1 conformation for this part of the study. As two such PDB entries, 5XR8 and 5XRA, are of similar quality and possess ligands from the same chemotype (Table 1 and Figure 1B), we chose 5XR8, because of its less truncated N-terminus, providing more complete structure for the sake of MD simulations. We screened three ligand libraries: DrugBank-approved, HMDB Drug Metabolites, and Biopurify Phytochemicals subsets from the ZINC database (∼10,000 compounds). Initially, the molecules were docked to CB1 model based on PBD ID: 5XR8 using CDOCKER. The energies of the ligands were minimized in situ and the complexes were scored with PMF04 function. According to docking results and subsequent analysis, 40 ligands were selected for MD verification of their putative binding poses. The CB1–ligand complexes were embedded in lipid bilayer and full-atom MD simulations were conducted using GROMACS. In each case, we performed three repetitions per complex (see Methods). Based on the docking scores and root-mean-square deviation (rmsd) of the ligands’ heavy atoms from their initial geometries across MD simulations, we selected 22 drugs and phytochemical compounds for the in vitro binding assay (Table S1, Figures S2 and S3).

#### 2.1.2 Second Iteration

**Figure 2:**
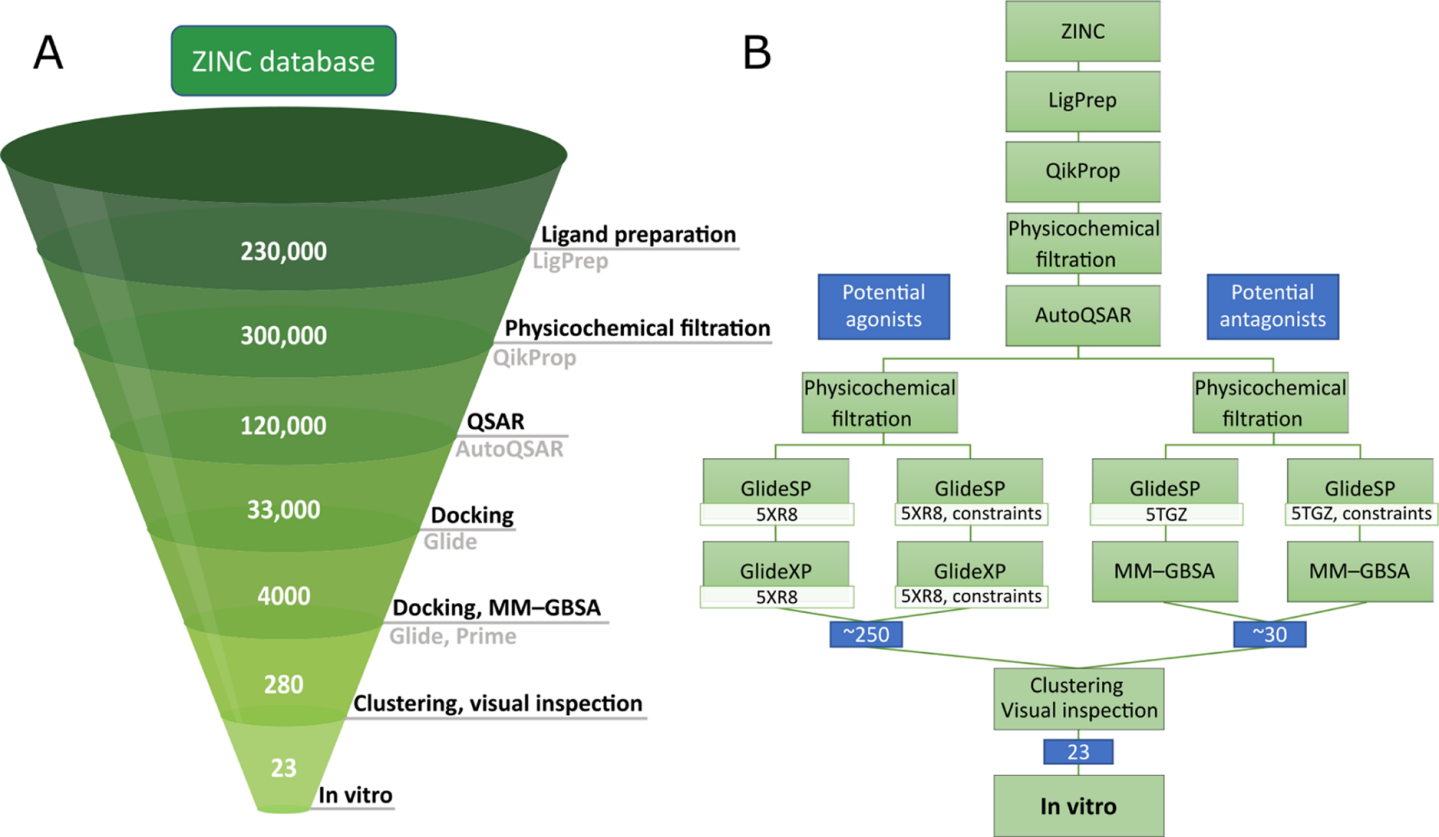
Computational workflow used in the second iteration of screening. (A) Main steps of the procedure with the approximate numbers of compounds left after each phase. The number of compounds initially increased because of the generation of possible ionization states and tautomers during ligand preparation. (B) Detailed order of the methods used, along with the branching off the workflow for potential agonists and antagonists.

In the second iteration, we combined SB and LB methods. The LB techniques were implemented to allow for the efficient screening of larger ligand libraries and to alleviate the inaccuracy of the SB approach when used alone for such a hydrophobic molecular target as CB1. In this part of the study, we screened ∼230,000 compounds, including drugs, drug metabolites, phytochemicals, and natural-like compounds from the following libraries derived from the ZINC database: DrugBank-approved, HMDB Drug Metabolites, SMPDB, and ZINC biogenic subset (the libraries comprised all compounds from the first iteration). Based on the conclusions derived from the first iteration, we established and followed a multi-step workflow (Figure 2).

The first major part of the computational procedure involved filtration of physicochemical properties. The ligands were prepared using Schrödinger LigPrep, including generation of possible ionization states and tautomers, which resulted in ∼300,000 structures. Then, their physicochemical properties were computed with QikProp. For the filtration, we adopted our own criteria, mainly based on Lipinski’s and Veber’s rules, with less strict cutoffs for molecular weight (MW) (≤600 g/mol) and the number of rotatable bonds (≤20) as well as an altered range for log *P* (3–8). The first two changes were introduced due to multiple CB1 ligands, mainly antagonists, possessing MW above 500 g/mol and endocannabinoids having in some cases even up to 25 rotatable bonds. The modified criterion for log *P* was meant to account for high hydrophobicity of CB1 orthosteric binding site and thus also CB1 ligands (Figure S4). The filtration allowed for considerable reduction of compounds pool to ∼120,000.

In the next part of the second iteration, we estimated the p*K*_i_ values of the remaining compounds with machine learning quantitative structure–activity relationship (QSAR) models. This phase of the screening was conducted using Schrödinger AutoQSAR. We based our models on ChEMBL-deposited compounds with known *K*_i_ values toward human CB1 (nearly 5000 entries at the time of the study). Those molecules were prepared in Schrödinger suite and then we applied the same criteria for the physicochemical properties as for the screening libraries. This filtration resulted in 2355 non-redundant compounds, based on which ten QSAR models were prepared. They exhibited desired binding affinity prediction capability, based on the statistical parameters (Table S2 and Figure S5). We utilized them to perform a consensus prediction of p*K*_i_ values for the screening compounds. We retrieved molecules that were within the applicability domains of all QSAR models, as labelled by built-in function of Schrödinger AutoQSAR, and obtained the estimated average p*K*_i_ value ≥6.5.

The resulting ∼33,000 potential CB1 ligands were subjected to the next stage of the VS procedure, employing mainly SB methods. At this point, we divided the workflow into two branches. The first was aimed to identify potential CB1 agonists and the second—antagonists or inverse agonists (Figure 2B). In both branches, the remaining compounds were once again filtered based on their computed physicochemical properties, this time with specific criteria modified for potential agonists and antagonists (Table S3). In the case of agonists, we altered the cutoffs for MW (≤500 g/mol) and log *P* (3.5–8). For antagonists, we reduced the maximal number of rotatable bonds to 10 (see Comments on the Virtual Screening Procedure).

**Figure 3:**
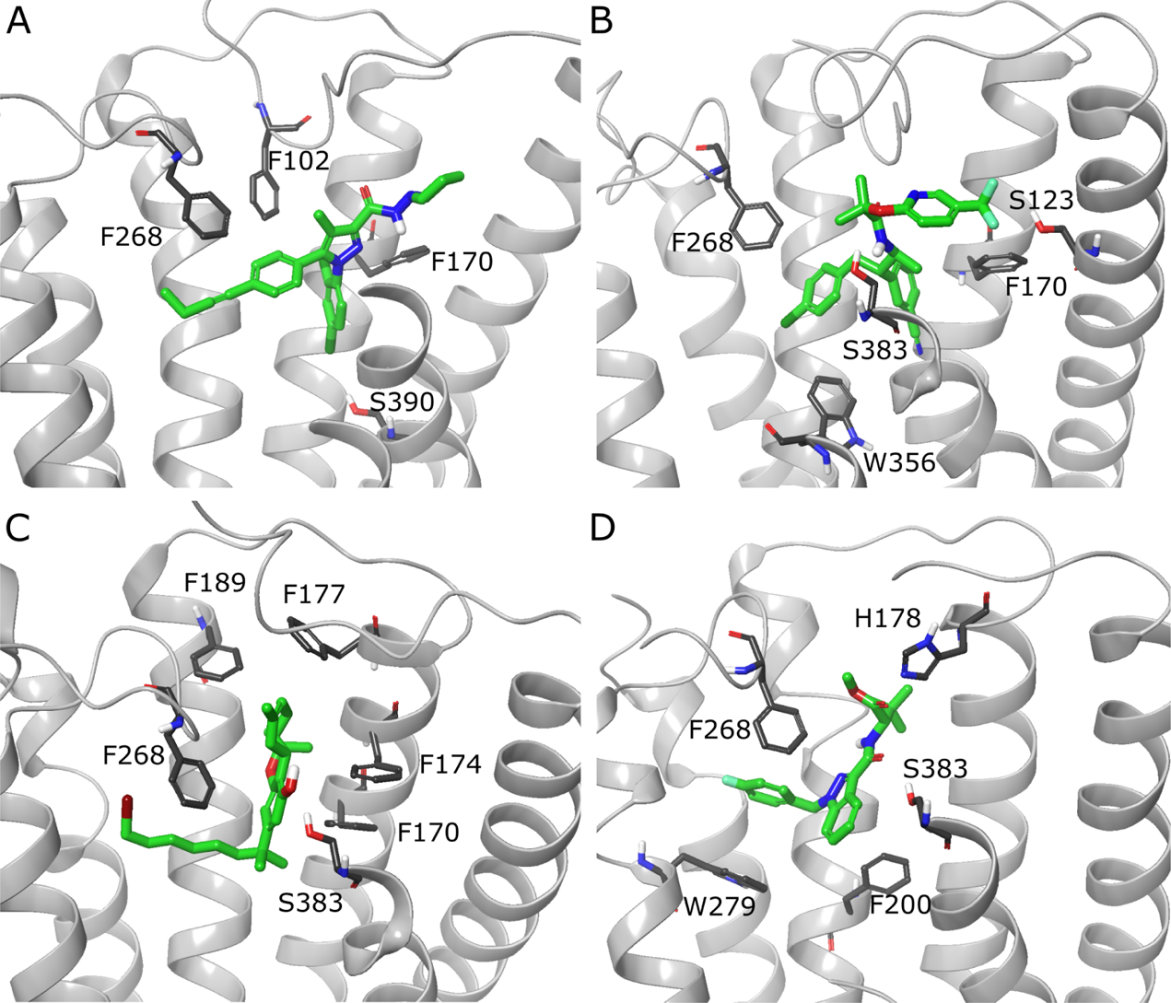
CB1 binding sites with ligands from four chemotypes present in PDB-deposited CB1 structures. Complexes with antagonists/inverse agonists: (A) CB1-AM-6538 (PDB ID: 5TGZ), (B) CB1-taranabant (PDB ID: 5U09). Complexes with agonists: (C) CB1-AM-11542 (PDB ID: 5XRA), (D) CB1-MDMB-Fubinaca (PDB ID: 6N4B). Ligands are depicted in green stick representation, amino acids that are crucial or could take part in ligand binding—grey stick representation. Parts of TM6 and TM7 not shown to increase readability.

The next step consisted of docking the two sets to specific CB1 models using Schrödinger Glide with standard precision (SP). Based on prior validation of binding affinity prediction (see Methods), we selected one active CB1 conformation for potential agonists (PDB ID: 5XR8) and one inactive for potential antagonists (PDB ID: 5TGZ). For each set, we performed two versions of docking—standard one and one with constraints on forming an H-bond with Ser383 hydroxyl group (Figure 2B), as this interaction was shown to be important for ligand binding in three out of four ligand chemotypes present in PDB-deposited CB1 structures (Figure 3). We retained the compounds with docking score values ≤−10. Then, the potential agonists were redocked using Glide extra precision (XP). In turn, due to differences in the binding site conformation (see Comments on the Virtual Screening Procedure), in the case of potential antagonists, we calculated the molecular mechanics-generalized Born surface area (MM-GBSA) binding free energies of the CB1–ligand complexes obtained from the Glide SP docking.

For the final analysis, we selected compounds with Glide XP docking score ≤−12 or MM-GBSA Δ*G*_bind_ ≤−85 kcal/mol. Additionally, we took into account several potentially interesting drugs that obtained results close to fulfilling the criteria. The resulting molecules were subjected to an auxiliary structural clustering, which was of assistance especially for potential agonists, ∼250 of which met the previous screening criteria. Selected compounds were analyzed with the focus on their binding poses and interactions formed with the receptor. Also, we took into account docking scores, MM-GBSA Δ*G*_bind_, and QSAR-predicted p*K*_i_ values. We aimed to select structurally diverse compounds. We identified several known CB1 and/or CB2 ligands or their close derivatives, which we did not take into account for further verification. Finally, the commercial availability of the most promising candidates was evaluated. As a result of this procedure, we hand-picked 23 compounds for the in vitro binding assay (Table 2, Table S4, Figures S6 and S7).

**Table 2:** Results of the Second Iteration of Virtual Screening^a^

		5XR8				5TGZ			
ID	pred. p*K*_i_	SP DS	XP DS	SP-constraints DS	XP-constraints DS	SP DS	MM-GBSA Δ*G*_bind_ (kcal/mol)	SP-constraints DS	MM-GBSA-constraints Δ*G*_bind_ (kcal/mol)
23	6.7	–11.3	–13.4	–11.3	–13.4	–9.6		–10.0	
24	6.5	–10.7	–13.4	–10.2	–13.5	–8.4		–7.9	
25	6.5	–11.3	–13.1			–8.7			
26	6.7	–10.5	–13.1	–10.8	–13.0	–8.9		–7.4	
27	6.6	–11.9	–13.0			–8.5			
28	6.6	–12.2	–13.7	–12.0	–13.7	–9.3		–9.4	
29	6.6	–10.1	–12.5	–10.3	–12.4	–8.8			
30	6.5	–10.9	–14.0	–10.7	–13.7	–8.9			
31	6.6	–10.4	–13.2						
32	6.5	–12.0	–12.3	–12.1	–13.0	–9.5		–10.1	–44.8
33	6.6	–10.1	–11.6			–11.2	–59.6	–4.7	
34	6.5	–9.6				–10.1	–85.0	–10.0	–86.7
35	6.6					–10.3	–93.7	–10.2	–95.5
36	6.6					–11.2	–80.7	–11.4	–91.5
37	6.8					–11.8	–93.9		
38	6.6	–11.0	–11.9	–10.0		–10.2	–71.1	–10.5	–86.3
39	6.7					–10.8	–83.4	–10.2	–85.5
40	6.7					–11.9	–85.2	–12.0	–62.5
41	6.6					–10.6	–85.9	–10.3	–82.8
42	6.7	–7.4				–10.7	–89.0	–8.5	
43	6.5	–9.6				–10.7	–86.0	–11.4	–82.9
44	6.8	–11.3	–10.0	–6.4		–10.3	–84.8	–8.7	
45	6.6	–8.3				–11.5	–71.3	–11.5	–71.1

a DS, docking score.

### 2.2 In Vitro Binding Affinity and Intrinsic Activity Assays

**Figure 4:**
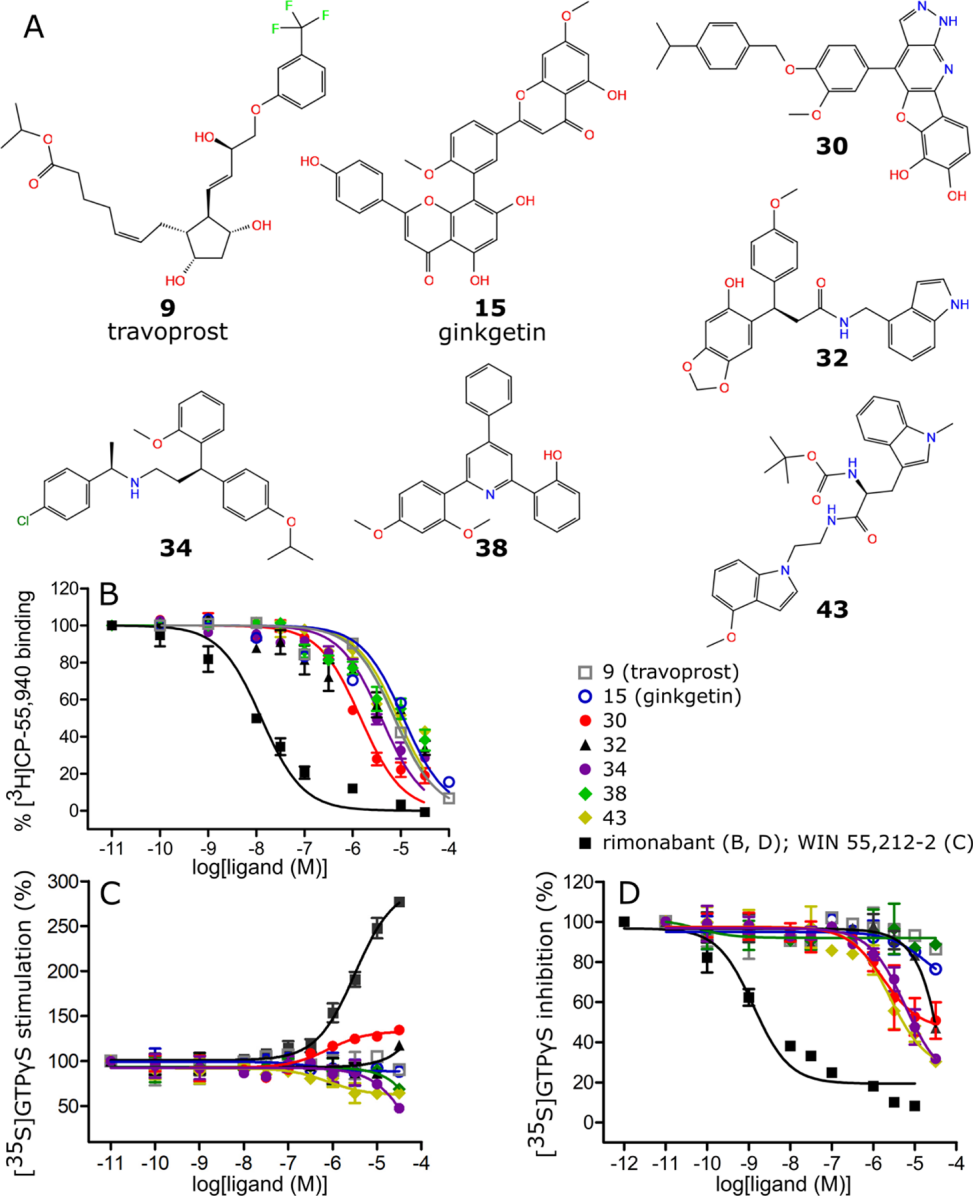
Structural formulas (A), radioligand displacement curves (B) and [^35^S]GTPγS functional curves (C,D) of the compounds with best *K*_i_ values toward CB1.

CB1 binding affinities of 45 compounds selected from both iterations of virtual screening were evaluated in the radioligand displacement assay. Seven ligands exhibited submicromolar or low micromolar affinities toward CB1 (Figure 4A,B). Two molecules from the first iteration showed low micromolar affinities: compound **9** (travoprost) obtained a *K*_i_ value of 3.6 μM, while compound **15** (ginkgetin)—8.6 μM. The most potent CB1 ligand identified during the second iteration, compound **30**, exhibited submicromolar affinity of 0.8 μM. Four other ligands from the second iteration, compounds **32**, **34**, **38**, and **43** obtained low micromolar *K*_i_ values of 6.8, 2.7, 2.5, and 6.6 μM, respectively. Compounds **32** and **34** were tested as racemic mixtures. Thus, their specific enantiomers identified during VS could possess lower *K*_i_ values (Table 3). The other compounds obtained *K*_i_ values >10 μM or showed no affinity toward CB1 (Tables S5 and S6).

**Table 3:** Selected Results of the *K*_i_ Determination with the In Vitro Binding Assay

ID	ZINC ID	name	p*K*_i_ ± SEM	*K*_i_ (μM, 95% CI)
9	ZINC000004474682	travoprost	5.40 ± 0.14	3.6 (1.8–7.4)
15	ZINC000001531664	ginkgetin	5.06 ± 0.16	8.6 (3.8–19.2)
30	ZINC000217658088		6.07 ± 0.09	0.8 (0.6–1.1)
32^a^	ZINC000824654462		5.20 ± 0.10	6.8 (3.8–10)
34^a^	ZINC000001832514		5.57 ± 0.07	2.7 (1.9–3.7)
38	ZINC000006040794		5.60 ± 0.09	2.5 (1.5–4.1)
43	ZINC000263585252		5.18 ± 0.07	6.6 (4.7–9.4)
	ZINC000001540228	rimonabant (reference)	8.30 ± 0.13	0.0043 (0.0023–0.0088)

a Racemic mixture.

Intrinsic activities of seven identified CB1 ligands were evaluated in the [^35^S]GTPγS ([^35^S] guanosine 5′-[γ-thio]triphosphate) assay (Figure 4C,D and Table 4). Compounds **9**, **15**, **32**, **34**, **38**, and **43** were determined to act as antagonists, with **34** and **43** obtaining IC_50_ values of 3.7 and 1.9 μM, respectively. Compound **30** showed interesting functional activity of mixed CB1 partial agonist/antagonist, with EC_50_ = 0.42 μM and IC_50_ = 2.1 μM.

**Table 4:** Results of the Intrinsic Activity Determination with the [^35^S]GTPγS assay

			CB1 inhibition		CB1 stimulation		
ID	ZINC ID	name	pIC_50_	IC_50_ (μM, 95% CI)	pEC_50_	EC_50_ (μM, 95% CI)	*E*_max_ (%)
9	ZINC000004474682	travoprost	3.64 ± 0.30	232 (34–782)			
15	ZINC000001531664	ginkgetin	4.20 ± 0.16	63 (29–144)			
30	ZINC000217658088		5.68 ± 0.06	2.1 (1.5–2.7)	6.38 ± 0.26	0.42 (0.12–1.48)	126.2 ± 5.0
32^a^	ZINC000824654462		4.48 ± 0.11	40 (23–71)			
34^a^	ZINC000001832514		5.43 ± 0.08	3.7 (2.6–5.5)			
38	ZINC000006040794		3.85 ± 0.28	140 (36–601)			
43	ZINC000263585252		5.73 ± 0.09	1.9 (1.2–2.9)			
	ZINC000001540228	rimonabant (reference)	8.88 ± 0.18	0.0013 (0.0005–0.0032)			

a Racemic mixture.

### 2.3 Analysis of the Binding Modes

**Figure 5:**
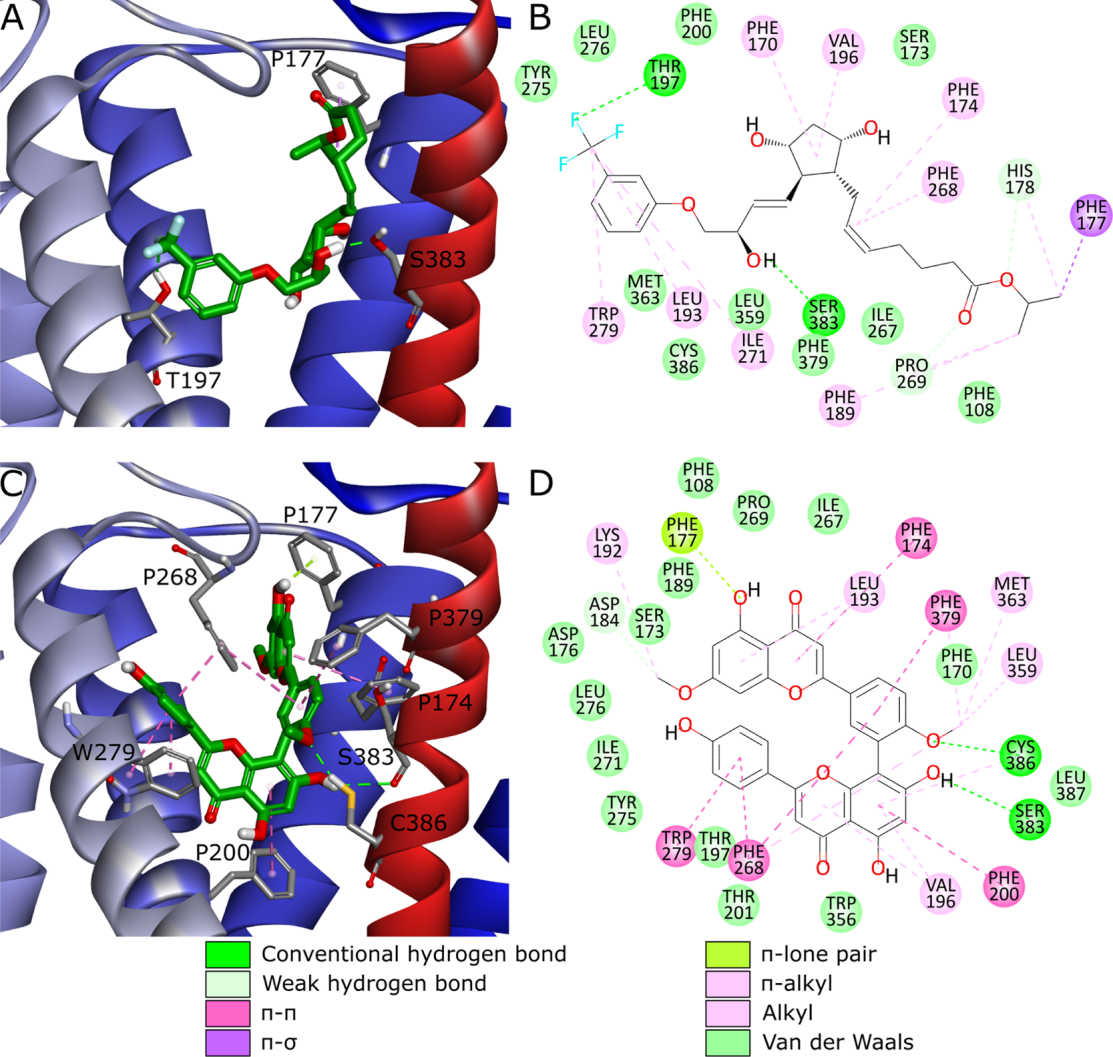
Two most potent CB1 ligands from the first iteration—travoprost (compound **9**, ZINC000004474682) (A,B) and ginkgetin (**15**, ZINC000001531664) (C,D). Putative poses and binding modes obtained from docking to PDB ID: 5XR8. Ligands are depicted in green stick representation, amino acids that are crucial for ligand binding—grey stick representation. Part of TM6 not shown to increase readability.

Seven compounds that exhibited the highest binding affinities toward CB1 were analyzed in detail regarding their putative binding modes. The ligands from the first iteration of VS were docked to CB1 active conformation based on PDB ID: 5XR8. Compound **9** forms H-bonds with Ser383 and Thr197 as well as multiple weak hydrophobic interactions (Figure 5A,B). Interestingly, binding mode of **9** relies heavily on H-bonds and lacks π–π interactions with several Phe residues present in the binding site, which are usually crucial for CB1–ligand complexes (Figure 3). On the other hand, compound **15** creates H-bonds with Ser383 and Cys386 but also π–π interactions with Phe174, Phe200, Phe268, Trp279, and Phe379, and additionally other weak hydrophobic interactions (Figure 5C,D). This binding mode is more alike to those observed in PDB-deposited structures (Figure 3), rather than the one discussed in the case of compound **9**. Notably, both ligands maintained their putative binding modes during MD simulations (Figure S8). Importantly, compound **9** showed also promising results during the second iteration (Table S7). However, in this case it achieved desired scores for the inactive CB1 conformation (PDB ID: 5TGZ) and assumed a distinct binding mode (Figure S9).

Compounds **30**, **32**, **34**, **38**, and **43** were docked to active and inactive CB1 conformations, based on PDB IDs: 5XR8 and 5TGZ, respectively. Moreover, we also conducted docking with constraints on forming an H-bond with Ser383. Additionally, in the case of active CB1 conformation, docking was performed with SP and XP Glide modes. Thus, we obtained multiple CB1–ligand complexes for the candidates from the second iteration. Compounds **30** and **32** were selected because of their high docking scores for CB1 active conformation. Compounds **34**, **38** and **43** achieved desired docking scores and Δ*G*_bind_ for inactive CB1 conformation. Interestingly, **32** showed also promising results for the inactive binding site conformation (5TGZ), while **38**—for the active one (5XR8).

Compounds **30**, **34**, and **43** assumed consistent putative binding modes, regardless of the docking settings, and exhibited only small differences in conformations and interaction patterns. Compound **32** showed the same pose in three versions of docking (SP, SP with constraints, and XP with constraints) while an alternative one for XP. In turn, compound **38** adopted three different binding poses, depending on CB1 conformation and settings.

**Figure 6:**
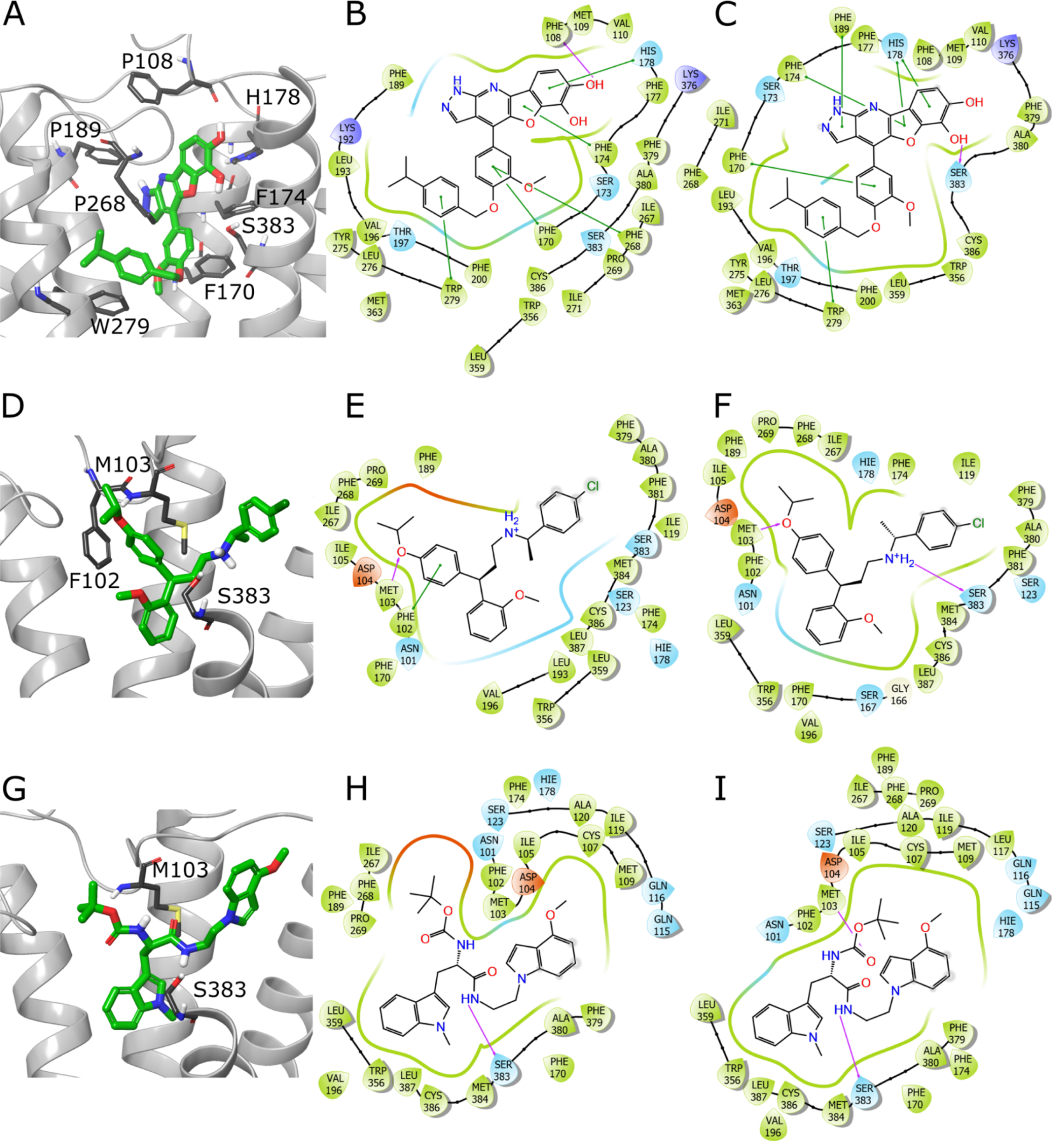
Putative binding modes of compounds **30** (ZINC000217658088), **34** (ZINC000001832514), and **43** (ZINC000263585252). Compound **30** pose obtained from docking with Glide XP (A) and 2D interaction schemes from Glide XP (B) and Glide XP with constraints on forming an H-bond with Ser383 (C). Analogically, panels (D–F) depict poses and binding modes predicted by Glide SP for compound **34**, while panels (G–I) for **43**. Ligands are depicted in green stick representation, amino acids that are crucial for ligand binding—grey stick representation. Purple arrow—H-bond; green line—π–π interaction. Parts of TM6 and TM7 not shown to increase readability.

**Figure 7:**
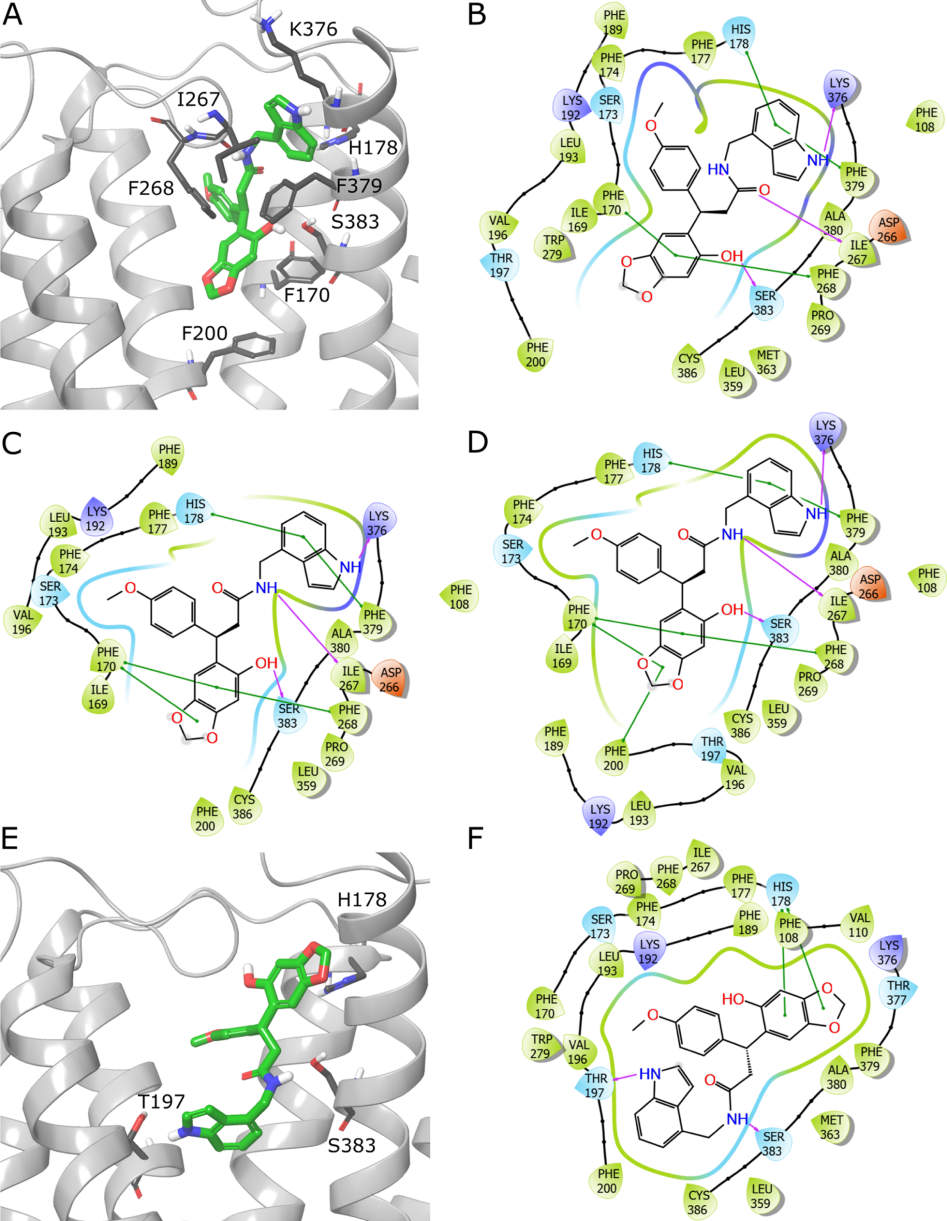
Putative binding modes of compound **32** (ZINC000824654462). (A) The more prevalent pose, obtained from docking with Glide XP with constraints on forming an H-bond with Ser383. 2D interaction schemes from docking with Glide XP with constraints (B), SP (C), and SP with constraints (D). Panels E, F show the second pose encountered in complex from Glide XP docking. Ligands are depicted in green stick representation, amino acids that are crucial for ligand binding—grey stick representation. Purple arrow—H-bond; green line—π–π interaction. Part of TM6 not shown to increase readability.

Compound **30** forms H-bonds with either Ser383 or Phe108. Additionally, this ligand maintains multiple π–π interactions with Phe170, Phe174, His178, Phe189, Phe268, and Trp279 (Figure 6A–C). The binding modes of compounds **34** and **43** are rather scarce in terms of the number of strong contacts, especially of the π-stacking character. Both ligands form H-bonds with Met103 and Ser383 and, in the case of compound **34**, also a π–π interaction with Phe102 (Figure 6D–I). The more prevalent putative pose of compound **32** forms H-bonds with Ile267, Lys376, and Ser383 as well as π–π interactions with Phe170, His178, Phe200, Phe268, and Phe379 (Figure 7A–D). On the other hand, the second predicted pose displays a sparse interaction pattern including H-bonds with Thr197 and Ser383, and π–π interaction with His178 (Figure 7E,F). Compound **38** is able to form a diverse set of interactions, depending on the putative pose. As this ligand is quite symmetrical in terms of its three phenyl groups branching from the pyridine core, some of the π–π interactions occur consistently, including those with the side chains of Phe102, Phe170, and Phe268. In some cases compound **38** forms H-bond with Ser383 (Figure S10).

### 2.4 Prediction of Pharmacological and Toxicological Properties

Additionally to the binding affinity and intrinsic activity determination, for the newly identified CB1 ligands, we assessed pharmacological and toxicological properties to gain more insight into their potential for further optimization or utilization. Because travoprost, as an approved drug, is a well-inspected compound, we excluded it from this part of the study.

Pharmacological properties were estimated using SwissADME server. Selected properties are shown in Table 5. We considered water solubility, gastrointestinal (GI) absorption, blood–brain barrier (BBB) permeation, P-glycoprotein (Pgp) binding and inhibition of the most important cytochrome P450 isoforms. As expected, because of high hydrophobicity, the ligands exhibit only moderate or poor solubility. Interestingly, all compounds were predicted to be unable to cross the BBB, which is a very desired trait in terms of the advantages of CB1 peripheral ligands. Such compounds could display better safety profile while still being effective in multiple indications.^10,30^ However, these predictions have to be taken with caution, as ginkgetin (compound **15**) was shown to exhibit cerebral activity in several in vivo models.^31,32^ In the case of compound **30**, potentially low GI absorption might be a matter to address during further stages of optimization.

**Table 5:** Selected Pharmacological Parameters Estimated for Newly Identified CB1 Ligands^a^

		water solubility					cytochrome P450 inhibition				
ID	ZINC ID	ESOL LogS	ESOL Class	GI absorption	BBB permeant	Pgp substrate	CYP1A2	CYP2C19	CYP2C9	CYP2D6	CYP3A4
15	ZINC000001531664	–7.17	poorly soluble	low	no	no	no	no	yes	no	no
30	ZINC000217658088	–6.93	poorly soluble	low	no	no	no	yes	no	yes	no
32	ZINC000824654462	–5.01	moderately soluble	high	no	yes	yes	yes	yes	yes	yes
34	ZINC000001832514	–6.63	poorly soluble	low	no	yes	no	no	no	yes	yes
38	ZINC000006040794	–5.90	moderately soluble	high	no	Yes	yes	Yes	no	yes	yes
43	ZINC000263585252	–4.99	moderately soluble	high	no	yes	no	yes	yes	yes	yes

a BBB, blood–brain barrier; CYP1A2, cytochrome P450 1A2; ESOL, Estimated SOLubility; GI, gastrointestinal; Pgp, *P*-glycoprotein.

In the case of toxicological properties prediction, given its often limited accuracy or chemical class-dependence,^33^ we utilized two programs—Toxtree and Toxicity Estimation Software Tool (TEST), and critically analyzed the results where possible. With the use of Toxtree, we determined Kroes threshold of toxicological concern (TTC)^34^ and Ames mutagenicity.^35^ In the case of TEST, we estimated developmental toxicity and also Ames mutagenicity, for comparison with Toxtree (Table 6).

**Table 6:** Results of the Toxicity Prediction for the New CB1 Ligands Identified among Natural and Natural-like Compounds^a^

		Toxtree		TEST			
		Kroes TTC	Ames mutagenicity	Ames mutagenicity		developmental toxicity	
ID	ZINC ID			predicted value	result	predicted value	result
15	ZINC000001531664			0.23		0.80	positive
30	ZINC000217658088	negligible	structural alert	0.14		0.73	positive
32	ZINC000824654462			0.21		0.76	positive
34	ZINC000001832514			0.19		0.59	positive
38	ZINC000006040794			0.71	positive	0.64	positive
43	ZINC000263585252	negligible	structural alert	0.51	positive	0.44	

a TTC, threshold of toxicological concern.

Kroes TTC estimation showed no safety concern in four and negligible risk in two compounds. Nearly all identified CB1 ligands were predicted to potentially exhibit developmental toxicity and could be not suitable for pregnant women and children. Interestingly, the Ames mutagenicity varied, depending on the software, confirming that the data should be interpreted with caution.

Compounds **30** and **43** received “negligible risk” flags or “structural alerts” in both Toxtree algorithms. Importantly, both notices applied to the same structural feature for each ligand. Compound **30** was flagged in two Toxtree tests based on its heteropolycyclic aromatic moiety. However, this is a structural feature encountered among many approved drugs and thus, does not raise concerns for the suitability of compound **30** for further optimization. Accordingly, this ligand was not flagged as mutagenic by the TEST program, and the low predicted Ames mutagenicity test value (0.14) suggests that there is a low probability of such outcome. On the other hand, compound **43** was consistently predicted as potentially mutagenic by both programs. In this case, Toxtree algorithms based their rating on the presence of alkyl carbamate moiety. Although this feature is also present among some approved drugs, it raises more substantial concerns^36,37^ and should be considered for further decision making on potential optimization. Other compounds raised no mutagenicity concerns or were flagged by only a single method.

### 2.5 Significance of the Results, Future Directions, and Limitations

Among drug active ingredients, travoprost (compound **9**) was found to possess low micromolar affinity to CB1. However, the promising *K*_i_ value did not convert to a relevant result in the functional assay. Nevertheless, travoprost is a prodrug, utilized in the treatment of ocular hypertension, including glaucoma. After topical administration it is hydrolyzed to its active metabolite—travoprost free acid. This compound acts as a FP prostanoid receptor agonist. However, its exact mechanism of action has not yet been determined.^38^ It was proved that CB1 takes part in the regulation of intraocular pressure and both CB1 agonists and antogonists could alleviate intraocular hypertension through different mechanisms.^39−41^ Thus, conducting studies regarding travoprost free acid’s impact on ECS may be a promising direction.

The most potent phytochemical encountered during the VS, ginkgetin (compound **15**), is a biflavone found most notably in *Ginkgo biloba* but also in several other plants. Ginkgetin exhibits a wide range of therapeutic properties, including neuroprotective, anti-adipogenic, anti-inflammatory, anti-oxidant, anti-microbial, and anti-cancer actions.^42^ Similarly to travoprost, despite low micromolar affinity to CB1, ginkgetin was found to possess insignificant functional activity. However, it is worth to bear in mind the entourage effect. Non-cannabinoid compounds are able to improve the pharmacological profiles of cannabinoid receptor ligands when used in combination.^43−45^ Thus, it is worth to conduct further research on the extracts from specific preparations of plants containing ginkgetin. This could be especially interesting in the context of some properties shared by ginkgetin and CB1 antagonists, namely anti-adipogenic and anti-inflammatory actions.^46,47^

Several natural-like compounds exhibited low micromolar affinities toward CB1, but most importantly, compound **30** achieved a *K*_i_ value of 800 nM. To the best of our knowledge, this molecule represents a new chemotype of CB1 ligands. Moreover, it serves as a rare example of a mixed CB1 partial agonist/antagonist. Therefore, despite moderate binding affinity, compound **30** is an interesting hit and could be introduced to hit-to-lead stage.

The structural distinctiveness of compound **30**, with respect to established CB1 ligands, is especially valuable in giving prospect for safe modulation of this specific receptor. Although multiple compounds with high affinities toward CB1 are known, they usually struggle with various issues, most notably with CB1-related adverse effects.^48−50^ Thus, multiple attempts, and consequently—diverse chemotypes among hits and leads might be needed to design a safe CB1 ligand.

Moreover, compound **30** is valuable due to its rare functional activity. Mixed agonists/antagonists are known to normalize the functions of the affected system. For example, pindolol, a β-adrenoreceptor antagonist, acts also as a partial agonist which is responsible for its intrinsic sympathomimetic activity.^51^ Conversely, CB1 partial agonists/antagonists could be very valuable due to the regulatory character of the ECS. In the case of CB1, such functional activity was observed under certain conditions for several tetrahydrocannabinol (THC) derivatives, for example Δ9-THC^52^ or O-823.^53^ Therefore, identification of CB1 partial agonist/antagonist representing another chemotype is a significant step toward rational design of such CB1 ligands.

Apart from *K*_i_ improvement, the major challenge of compound **30** optimization would lie in its high log *P* (5.2). However, such hydrophobicity is a standard issue for CB1 ligands (e.g. 5.8 for dronabinol). Moreover, high log *P* is a problem that medicinal chemistry community has been able to successfully tackle in multiple hit-lo-lead projects.^54^

### 2.6 Comments on the Virtual Screening Procedure

Utilization of a wide range of in silico methods across the two VS iterations allowed us to gain a valuable insight into the effectiveness of specific computational techniques in combination with CB1, a highly hydrophobic, non-trivial molecular target. The first iteration showed that solely SB methods struggle with a large amount of false positives. This issue was expected, nevertheless we decided to take the risk in order to increase the chances of encountering new chemotypes of CB1 ligands. The difficulties are caused mainly by the high hydrophobicity of CB1 orthosteric binding site (Figure S1). This hinders proper pose prediction as well as binding affinity estimation because of limited performance of most docking programs and scoring functions in the case of very lipophylic pockets.

A strategy to increase the true positive rate involves a combination of SB and LB methods. However, this approach comes with a cost of decreasing the chances of encountering new chemotypes and increasing the number of false negatives. We employed this strategy in the second iteration, which indeed allowed us to obtain a higher percentage of ligands with relevant *K*_i_ among the finally selected compounds (22 vs 9%, in comparison to SB only approach, respectively). Because the ligand set screened during the second iteration was broader than the one considered during the first iteration, a precise comparison between both approaches can not be made. However, focusing on in silico results of the second iteration screening limited to compounds considered during the first iteration shows that combination of SB and LB methods effectively tackled the false positives’ problem, rating highly only travoprost (Table S7).

The study also allowed us to draw conclusions regarding the ability of specific techniques to cope with troublesome CB1 docking. During the course of both iterations we utilized two docking programs—CDOCKER and Glide. They proved to perform quite similarly, both struggling with the hydrophobic binding site. Overall, Glide produced slightly better results in terms of the identification of CB1 ligands, as shown by *K*_i_ values obtained in the second iteration. Moreover, according to results of the second iteration’s in silico validation, in the case of many CB1 conformations, Glide SP exhibited similar or even superior ability to order ligands based on their binding affinities compared to Glide XP or MM-GBSA. Importantly, Glide XP docking performed for the purpose of validation was independent of SP docking, providing more adequate data for comparison than the sequential screening. What is more, Glide SP obtained high enrichment factors (EFs) for most of the CB1 structures, with EF_1%_ >20 for four and >10 for six out of seven tested CB1 conformations (Supporting Information S2). It is also worth to note the difficulties of docking programs in terms of proper binding pose prediction in some cases. This was caused mainly by non-specific π–π interactions with numerous Phe side chains in the CB1 binding site and was particularly evident for symmetrical-like ligands which possess aromatic rings in two or three vertices, for example taranabant or compound **38**.

In one of the last steps of the second iteration, we applied diverse cutoffs for physicochemical properties filtration (Table S3) as well as utilized Glide XP or MM-GBSA for potential CB1 agonists and antagonists (Figure 2B). The decision regarding the thresholds change for agonists was caused by the smaller volume of the active conformation of CB1 binding site.^23^ Thus, MW above 500 g/mol is attributed mainly to CB1 antagonists/inverse agonists. Also, the smaller volume of active CB1 conformation allows for the influx of even fewer water molecules to the binding site compared to the inactive conformation, which is already hardly accessible to the extracellular solvent. This imposes usually higher minimal hydrophobicity of CB1 agonists. Reduction of the maximal number of rotatable bonds to 10 for potential antagonists was caused by the occurrence of the higher values only in endocannabinoid-like CB1 agonists.

The reasoning behind the utilization of either Glide XP or MM-GBSA was based on validation results obtained during the second iteration. Both methods achieved high EFs for different CB1 models. In particular, MM-GBSA proved effective especially in the case of inactive geometry, represented by PDB ID: 5TGZ (EF_1%_ = 32.0), whereas Glide XP—for several active conformations: 5XR8 (EF_1%_ = 32.0), 5XRA (EF_1%_ = 24.0), and 6KQI (EF_1%_ = 20.0) (Supporting Information S2). This may be also explained by the disparity in the size of the orthosteric binding site between CB1 conformations and the subsequent exposure to solvent, as discussed above. As MM-GBSA is a method more accurate in terms of mimicking realistic solvent influence, it is reasonable that it copes better with more spacious binding site but struggles with the exceptionally lipophylic one.

Herein, we focused only on previously unidentified CB1 ligands with moderate to low affinity. However, it is worth to note that among the compounds placed highly by various methods during the second iteration, we encountered several known, potent CB1 ligands (Table 7). This includes compounds with very high affinity, for example rimonabant and nabilone, and the ones with moderately high affinity, such as bazedoxifene. Moreover, we encountered a few cannabinoids, mainly derivatives of cannabinol, with unspecified affinity toward CB1. This serves as an additional proof of concept and shows that the VS procedure is suitable to identify compounds with high affinity. Accordingly, we believe that the VS workflow used in the second iteration is also able to detect new, very potent compounds. To maximize the chances for obtaining such results, the final set selected for in vitro verification should include ligands that achieved both high docking scores or MM-GBSA binding energies and p*K*_i_ predicted by QSAR. Within the considered libraries, apart from already known CB1 ligands, we encountered compounds that fully matched only the first criterion. The calculated p*K*_i_ values came around 6.5–6.8 at best for ligands that also achieved desired SB results, whereas we would ideally expect p*K*_i_ values above 7. This suggests that there is a low probability of encountering any other CB1 ligands among the screened libraries but also that the procedure could be successfully employed to seek for potent CB1 ligands among other compound sets.

**Table 7:** Examples of Known CB1 Ligands or Cannabinoids Identified during the Second Iteration of Virtual Screening^a^

				5XR8				5TGZ			
ZINC ID	name	p*K*_i_	pred. p*K*_i_	SP DS	XP DS	SP-constraints DS	XP-constraints DS	SP DS	MM-GBSA Δ*G*_bind_ (kcal/mol)	SP-constraints DS	MM-GBSA-constraints Δ*G*_bind_ (kcal/mol)
ZINC000001540228	rimonabant	8.2^55^	7.7	–8.2				–11.4	–94.5	–11.2	–94.6
ZINC000001542930	nabilone	8.7^56^	8.1	–11.2	–12.3	–11.1	–12.3	–7.4		–7.8	
ZINC000001895505	bazedoxifene	6.1^17^	6.5	–11.6	–7.8			–6.0			
ZINC000002008394	11-hydroxy-Δ9-THC		7.1	–11.0	–12.1	–10.9	–12.0	–8.2		–8.4	
ZINC000005821102	9-α,10-α-epoxyhexahydrocannabinol		6.7	–10.2	–11.2	–10.2	–11.2	–6.9		–8.4	

a DS, docking score.

Our computational procedure was established to identify new, possibly potent and structurally diverse CB1 ligands. Although we utilized active and inactive CB1 conformations and divided compounds into potential agonists and antagonists at specific stages, these actions were taken mainly for the sake of the aforementioned aims. It is important to state that in silico methods utilized in this study are not suited for reliable intrinsic activity estimation. This was confirmed by the discrepancy between loose, computational predictions and the [^35^S]GTPγS assay. Namely, out of seven identified CB1 ligands, two appeared in silico as possible agonists (compounds **15** and **30**), two as antagonists (**34** and **43**), and the latter three (**9**, **32** and **38**) showed ambiguous results. Therefore, estimating functional activity based on VS methods has to be taken with caution. Nevertheless, other, more time-costly in silico approaches, developed specifically for the discrimination of ligand efficacy, have been demonstrated for CB1.^57^

## 3 Conclusions

We conducted a double-iterative, multi-step, computational screening to seek for novel CB1 ligands among active drug ingredients, their metabolites, phytochemicals, and natural-like compounds. We selected 45 candidates for verification with an in vitro binding assay and, in the case of specific compounds, also with a functional assay. We identified travoprost and ginkgetin as CB1 ligands with low micromolar affinity. This finding may act as a starting point for further research on the impact of metabolites or preparations of these compounds on ECS. Moreover, we identified five natural-like compounds with submicromolar or low micromolar affinity toward CB1. The most potent CB1 ligand found, compound **30** (ZINC000217830653), because of its structural distinctiveness and rare mixed partial agonist/antagonist functional activity, could be considered in future hit-to-lead endeavours. Finally, the computational procedure established during this work will be of use for other VS campaigns aimed to search for novel CB1 ligands, especially in broader, synthetic libraries.

## 4 Methods

### 4.1 First Iteration of Virtual Screening

The first iteration of virtual screening included docking with BIOVIA Discovery Studio 2018^58^ and subsequent verification of the results with molecular dynamics in GROMACS.^59^

Screening compounds were downloaded from the ZINC15 database.^60^ The molecules were prepared in BIOVIA Discovery Studio 2018^58^ using Prepare Ligands protocol with the generation of possible ionization states in pH 7.5 ± 1. The CB1 model based on PDB ID: 5XR8(23) was prepared with Prepare Proteins protocol. A spherical gridbox with a radius of 12 Å was created around the crystal ligand. Docking was conducted using CDOCKER^61^ with the maximum of ten poses saved per compound. Ligands were scored with PMF04 function.^62^ For the best pose for each docked molecule, the energy of the ligand was minimized with In Situ Ligand Minimization protocol. The obtained complexes were rescored with PMF04 function.

Selected CB1–ligand complexes were subjected to MD verification. Using Discovery Studio, we reverted the point mutations present in PDB ID: 5XR8 crystal structure, removed the fusion protein and reconstructed the intracellular loop 3 (ICL3) with Prepare Protein protocol, based on the human CB1 sequence from UniProt.^63^ We utilized CHARMM-GUI Membrane Builder^64^ to prepare the systems for simulations. The CB1–ligand complexes were embedded in 1-palmitoyl-2-oleoylphosphatidylcholine (POPC) lipid bilayer and the reminder of the system was filled with explicit water molecules and 0.15 M NaCl. Parametrization of the ligands was performed with Antechamber program from AmberTools 18 package,^65^ utilizing AM1-BCC charges and General Amber Force Field (GAFF).^66,67^ The reminder of the system preparation was conducted in GROMACS 2018.7.^59^ The Amber 99SB-ILDN force field^68^ was selected for the protein, Lipid 14^69^ for the membrane, and water model was set to TIP3P. We performed energy minimization with steepest descent algorithm consisting of maximum 10,000 steps. It was followed by several phases of equilibration with changing restraints: (1) *NVT* ensemble, *t* = 100 ps, position restraints on protein heavy atoms, force constant 1000 kJ/mol/nm^2^; (2) *NPT* ensemble phase 1, *t* = 5 ns, position restraints on protein heavy atoms, force constant 1000 kJ/mol/nm^2^; (3) *NPT* ensemble phase 2, *t* = 5 ns, position restraints on protein heavy atoms, force constant 100 kJ/mol/nm^2^; (4) *NPT* ensemble phase 3, three versions with *t* = 5–8 ns, position restraints on protein Cα atoms, force constant 1000 kJ/mol/nm^2^. Temperature, T was set to 310 K and time step, Δ*t* to 0.002 ps. Berendsen thermostat and pressure coupling were selected for equilibration. Then, the systems were subjected to three independent runs of 300 ns simulations with Δ*t* = 0.002 ps, Nose–Hoover thermostat, and Parrinello–Rahman semi-isotropic pressure coupling at 1 atm.

The MD trajectories were analyzed in VMD.^70^ We calculated rmsd of the ligands’ heavy atoms with respect to their starting positions. Beforehand, we had superimposed CB1 Cα atoms (excluding very flexible ICL3) on the starting conformation.

### 4.2 Second Iteration of Virtual Screening

#### 4.2.1 Preparation of Ligands and Proteins

Screening compounds’ libraries were downloaded from the ZINC15 database. The classification of compounds as ”natural-like” was based on the criteria established by the ZINC database for the assembly of its biogenic libraries.^71^ Test CB1 ligands were downloaded from PubChem.^72^ Compounds were prepared in Schrödinger Maestro 2017-1 software^73^ using LigPrep with generation of possible protonation states in the pH range 7.0 ± 2.0 with Epik and generation of possible tautomeric forms.

The CB1 structures were downloaded from the PDB. They were prepared in Maestro 2017-1 with Protein Preparation Wizard. Water molecules and other redundant, post-crystallization small molecules were removed. The CB1 structures were preprocessed, including the addition of hydrogen atoms and the generation of probable protonation states using Epik in the pH 7.0 ± 2.0. The H-bond assignment was optimized using PROPKA and structures were minimized using the OPLS3 force field.^74^

#### 4.2.2 Physicochemical Properties Filtration and QSAR

Initial preparation of compounds for the QSAR part of the study included the calculation of their physicochemical properties with Schrödinger QikProp. Then, they were filtered with a custom set of criteria that included the combination of properties from Lipinski’s^75^ and Veber’s^76^ rules and properties observed for most of the CB1 ligands (Figure S4): MW ≤600 g/mol; log *P* 3–8; number of hydrogen bond acceptors ≤10; number of hydrogen bond donors ≤5; number of rotatable bonds ≤20, and polar surface area (PSA) ≤140 Å^2^.

Generation of QSAR models and subsequent screening were performed with Schrödinger AutoQSAR.^77^ Training and test compounds for QSAR models were downloaded from the ChEMBL database.^28,29^ We selected 2549 compounds with *K*_i_ values known for human CB1. Compounds were prepared in LigPrep without generation of protonation states or tautomers. Then, we generated probable protonation states and tautomeric forms in a separate Epik protocol in the pH 7.0 ± 2.0 with the maximum number of output structures per molecule set to 1. Physicochemical properties of prepared training/test compounds were calculated with QikProp. Then, we filtered compounds with the same set of criteria as the screening compounds, leaving 2355 training/test compounds. We used AutoQSAR protocol to generate QSAR models. Random training set was set to 75% compounds. Prediction property was set to p*K*_i_. Consensus prediction of p*K*_i_ values was performed for the screening compounds by taking an average for all QSAR models. Only results with Domain Alert = 0 were kept.

#### 4.2.3 Docking and MM-GBSA

Docking was conducted in Schrödinger Maestro 2017-1. The first step consisted of preparation of grid files for the previously prepared CB1 structures. We utilized Receptor Grid Generation Tool and created grids using ligands as the grids’ centers. For each CB1 structure, two grid files were created—a standard one and one with constraints for the formation of H-bonds with Ser383 hydroxyl group.

The docking itself was conducted using Glide^78,79^ with SP and XP modes. One best pose for ligand in each docking was saved. MM-GBSA binding free energy calculations were preformed using Prime MM-GBSA with VSGB solvation model^80^ and OPLS3 force field.

Before the actual screening, our docking procedure has been validated. We conducted a test screening using two sets of active test compounds and two sets of decoys. We prepared separate libraries of active CB1 ligands for agonists and antagonists/inverse agonists. Each set contained 25 compounds with *K*_i_ values toward human CB1 ≤100 nM (Tables S8 and S9). Based on these two sets, we generated two libraries of similar decoys (1250 compounds each) using DUD-E server.^81^ Active compounds and decoys were prepared using Schrödinger LigPrep. Decoy sets were filtered based on their physicochemical properties calculated in QikProp, using custom criteria (Table S3). As receptor models, we used CB1 structures based on PDB IDs: 5TGZ,^21^5U09,^22^5XR8, 5XRA,^23^6N4B,^24^6KPG,^26^ and 6KQI.^25^ We performed Glide SP and XP docking (independent of each other) and subsequent MM-GBSA binding free energy calculations. The agonists active and decoys sets were docked to PDB IDs: 5XR8, 5XRA, 6N4B, 6KPG, and 6KQI, while antagonists to 5TGZ and 5U09. Using Schrödinger Enrichment Calculator, we determined Boltzmann-enhanced discrimination of the receiver operating characteristic (BEDROC) values and EF for docking scores and MM-GBSA Δ*G*_bind_ obtained after Glide SP and XP docking (Table S10, Figures S11 and S12 and Supporting Information S2).

Based on the validation results, we conducted docking of the screening compounds to PDB IDs: 5XR8 and 5TGZ. Prior to that, the compounds had been filtered using custom physicochemical criteria different for potential agonists and antagonists (Table S3), analogically to decoys. Both screening sets were processed using Glide SP, with potential agonists docked to 5XR8 and potential antagonists docked to 5TGZ. Finally, potential agonists were docked using Glide XP, while in the case of CB1-potential antagonists complexes, we conducted additional MM-GBSA binding free energy calculations.

#### 4.2.4 Clustering and Analysis

The selected output compounds from docking and MM-GBSA calculations were analyzed for the sake of subsequent in vitro evaluation. We clustered them based on the structural properties using ChemBioServer 2.0^82^ with Hierarchical Clustering. The properties were set to: distance selection: Soergel; clustering linkage selection: Ward; clustering threshold: 0.3. We analyzed compounds based on their QSAR-predicted p*K*_i_ values, docking score, and MM-GBSA binding energy. For selected compounds, we analyzed binding poses and protein–ligand interactions in Schrödinger Maestro. The final selection was based on the obtained numerical values, clustering, visual analysis, and purchasability, favoring compounds from diverse structural groups.

### 4.3 Pharmacological and Toxicological Properties Estimation

Pharmacological properties were estimated using SwissADME server.^83^ Water solubility was assessed with ESOL (Estimated SOLubility) model.^84^ Toxicological properties were computed using Toxtree 3.1.0^85^ and TEST 5.1.1^86^ programs. In Toxtree, the selected models were utilized: Kroes TTC decision tree^34^ and in vitro mutagenicity (Ames test) alerts,^87,88^ whereas in TEST—Ames mutagenicity and developmental toxicity.

### 4.4 In Vitro

#### 4.4.1 Radioligand Displacement Assay

Compounds selected for the in vitro binding assay from the first iteration of VS were purchased via MolPort, SIA, Riga, Latvia and Biopurify Phytochemicals Ltd., Chengdu, China. Compounds from the second iteration were purchased from MolPort, SIA, Riga, Latvia and Chemspace, SIA, Riga, Latvia (Supporting Information S3). [3H]CP-55,940 and the EcoScint-20 scintillation fluid were purchased from Perkin Elmer (USA). Membranes from cells overexpressing the human CB1 receptor (ChemiScreen CB1 Cannabinoid Receptor Membrane Preparation, cat. no. HTS019M) as well as MgCl_2_, CaCl_2_, Trizma Base, NaCl, polyethylenimine (PEI) and dimethyl sulfoxide (DMSO) were purchased from Merck (USA). Bovine serum albumin (BSA) was obtained from Pol-Aura (Poland). WIN 55,212-2 mesylate was provided by Tocris Bioscience (UK).

Membrane preparations (2.5 μg protein/tube) from Chem-1 cells expressing human CB1 receptors were incubated in duplicate with 2 nM [3H]CP-55,940 (specific activity: 108.5 Ci/mmole) in a 50 mM Tris-HCl, pH = 7.4 buffer supplemented with 5 mM MgCl_2_, 1 mM CaCl_2_, 0.2% BSA and increasing concentrations of the compounds tested. Compounds were dissolved in 50% DMSO and added to the reaction mixture at 10 concentrations equally spaced on a log scale (10^–10^ to 10^–4.5^ M). The final DMSO concentration was 5%. Non-specific binding was determined with 10 μM WIN 55,212-2. The reaction mixture (500 μL) was incubated for 1.5 h at 30°C . Before harvesting, Whatman GF/B Filter Paper (Brandel, USA) was presoaked with 0.33% PEI buffer for 30 min. and then washed with 2 ml of 50 mM Tris-HCl buffer (pH = 7.4) and 0.5% BSA to minimize non-specific binding. The reaction was terminated by depositing the samples onto GF/B filter paper with the Brandel M-24 Cell Harvester (Brandel, USA). Samples were then rapidly washed 3 times with 2 ml of ice-cold wash buffer (50 mM Tris-HCl pH 7.4, 500 mM NaCl) to separate the bound radioligand from free. Filters were then air-dried for 1 h at 60°C . After drying, filter discs were placed on a flexible 24-well plate and 500 μL of EcoScint-20 scintillant was added to each well. Plates were counted (2 min. per well) in a Trilux MicroBeta^2^ 2450 scintillation counter (Perkin Elmer, USA). Data were analyzed with GraphPad Prism 5.0 software.^89^ Curves were fitted with a one-site non-linear regression model and inhibitory constants (p*K*_i_ ± SEM and *K*_i_, 95% CI) were calculated from the Cheng–Prusoff equation.

#### 4.4.2 [^35^S]GTPγS Assay

Ten compound concentrations equally spaced on a log scale (10^–3^ to 10^–9^ M) were incubated in triplicate with membrane preparations from CHO-K1 cells expressing the human CB1 receptor (5 μg per well) (Perkin Elmer, cat. no. ES-110-M400UA) in an assay buffer containing 50 mM Tris-HCl, pH = 7.4, 1 mM EGTA, 3 mM MgCl_2_, 100 mM NaCl and 30 μM GDP) in the presence of 0.08 nM [^35^S]GTPγS (specific activity: 1250 Ci/mmole, Perkin Elmer). Non-specific binding was determined with 100 μM of unlabeled GTPγS. WIN 55,212-2 (3 μM) was used as stimulating ligand. The final DMSO concentration in the assay was 5%. The reaction mixture was incubated for 90 min. at 30°C . Next, the samples were deposited under vacuum with the FilterMate Harvester® (Perkin Elmer, USA) onto Unifilter® GF/B Plates (Perkin Elmer, USA) presoaked with wash buffer (50 mM Tris-HCl, pH = 7.4). The samples were then rapidly washed with 2 ml of wash buffer. Filter plates were dried for 30 min. at 50 °C and 40 μL of MicroScint PS (Perkin Elmer, USA) scintillation fluid was added to each well. Radioactivity was counted in a Trilux MicroBeta^2^ counter (Perkin Elmer, USA). Data were analyzed with GraphPad Prism 5.0 software. Curves were fitted with three-parameter non-linear regression model. Inhibitory potency (IC_50_) was calculated and expressed as means from three separate experiments ± 95% confidence intervals (95% CI).

## Supporting Information Available

Hydrophobic surface of CB1 binding site (Figure S1); computational results of the first and second iteration of VS (Tables S1 and S4); structural formulas of compounds selected for the in vitro binding assay (Figures S2, S3, S6, and S7); statistical parameters and correlation plots for QSAR models (Table S2 and Figure S5); histograms of selected physicochemical properties of CB1 ligands with with *K*_i_ ≤100 nM deposited in ChEMBL (Figure S4); cutoffs used in physicochemical properties screening (Table S3); results of the in vitro binding assay (Tables S5 and S6); rmsd plots from MD simulations of CB1 complexes with travoprost and ginkgetin (Figure S8); results of the second iteration methods applied for compounds selected from the first iteration (Table S7); putative binding mode of compound **9** obtained during the second iteration (Figure S9); putative binding modes of compound **38** (Figure S10); CB1 agonists and antagonists used in docking and MM-GBSA validation (Tables S8 and S9); selected results of docking and MM-GBSA validation (Table S10, Figures S11 and S12) (PDF)
Results of the docking and MM-GBSA validation of the second iteration (XLSX)
Information regarding suppliers and purity of the compounds selected for the in vitro binding assays. Chromatograms, spectra, and certificates of analysis provided by suppliers (ZIP)

## Abbreviations

[^35^S]GTPγS: [^35^S] guanosine 5′-[γ-thio]triphosphate
AM404: *N*-arachidonoylaminophenol
BBB: blood–brain barrier
BEDROC: Boltzmann-enhanced discrimination of the receiver operating characteristic
BSA: bovine serum albumin
CB1: cannabinoid receptor type 1
CI: confidence interval
Cryo-EM: cryoelectron microscopy
CYP1A2: cytochrome P450 1A2
DMSO: dimethyl sulfoxide
DS: docking score
*E*_max_: maximum effect
EC_50_: half maximal effective concentration
ECL2: extracellular loop 2
ECS: endocannabinoid system
EF: enrichment factor
ESOL: estimated SOLubility
GAFF: General Amber Force Field
GI: gastrointestinal
IC_50_: half maximal inhibitory concentration
ICL3: intracellular loop 3
*K*_i_: inhibition constant
LB: ligand-based
MD: molecular dynamics
MM-GBSA: molecular mechanics-generalized Born surface area
MW: molecular weight
NAM: negative allosteric modulator
PDB: Protein Data Bank
PEI: polyethylenimine
Pgp: *P*-glycoprotein
POPC: 1-palmitoyl-2-oleoylphosphatidylcholine
QSAR: quantitative structure–activity relationship
rmsd: root-mean-square deviation
SB: structure-based
SEM: standard error of measurement
SP: standard precision
THC: tetrahydrocannabinol
TM: transmembrane (helix)
TTC: threshold of toxicological concern
VS: virtual screening
XP: extra precision
XRD: X-ray diffraction
