# Supplementary material for: Identification of CB1 Ligands among Drugs, Phytochemicals and Natural-Like Compounds: Virtual Screening and In Vitro Verification
Source: ACS Chem Neurosci. 2022 Oct 5;13(20):2991–3007. doi: 10.1021/acschemneuro.2c00502 (PMC9585589; doi:10.1021/acschemneuro.2c00502)
Supplement: Supplementary file 1 — cn2c00502_si_001.pdf [file cn2c00502_si_001.pdf]

Supporting Information: Identification of CB1 Ligands among Drugs, Phytochemicals and Natural-Like Compounds: Virtual Screening and In Vitro Verification

Adam Stasiulewicz,[†,‡] Anna Lesniak,[¶] Piotr Setny,[‡] Magdalena Bujalska-Zadrożny,[¶] and Joanna I. Sulkowska[*,‡]

†*Department of Drug Chemistry, Faculty of Pharmacy, Medical University of Warsaw, Banacha 1, 02-097 Warsaw, Poland*

‡*Centre of New Technologies, University of Warsaw, Banacha 2c, 02-097 Warsaw, Poland*

¶*Department of Pharmacodynamics, Faculty of Pharmacy, Medical University of Warsaw, Banacha 1, 02-097 Warsaw, Poland*

E-mail: j.sulkowska@cent.uw.edu.pl

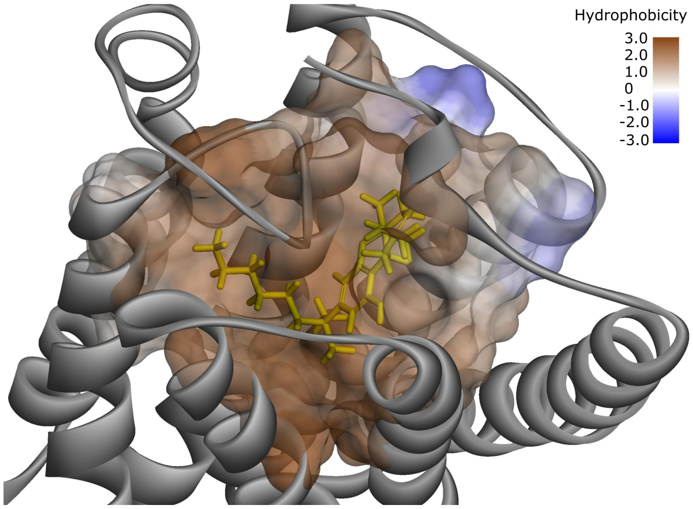

Figure S1: Hydrophobic surface of the CB1 binding site, based on PDB ID: 5XR8.

Table S1: Results of the first iteration VS for the compounds selected for in vitro binding assay.

ID	PMF04	RMSD (Å)		
		Trajectory 1	Trajectory 2	Trajectory 3
1	−151.4	1.7	2.5	1.9
2	−136.1	1.3	1.3	1.8
3	−137.0	1.2	1.3	1.3
4	−131.8	1.7	2.5	1.3
5	−149.4	1.3	1.2	1.7
6	−138.4	2.0	1.6	1.4
7	−144.0	0.8	0.9	1.0
8	−169.0	1.7	2.7	1.3
9	−153.7	1.9	1.1	1.8
10	−142.6	1.1	1.5	1.4
11	−166.7	1.5	1.1	1.9
12	−166.8	1.4	1.0	1.4
13	−159.0	1.2	3.1	2.4
14	−131.9	1.3	1.3	1.2
15	−164.5	1.5	1.6	1.4
16	−152.0	3.0	1.3	1.1
17	−173.6	1.4	2.7	1.0
18	−155.5	1.4	3.7	0.9
19	−148.3	2.7	1.6	1.9
20	−154.3	1.4	1.1	0.9
21	−151.4	0.7	1.5	0.7
22	−182.8	1.2	1.4	1.3

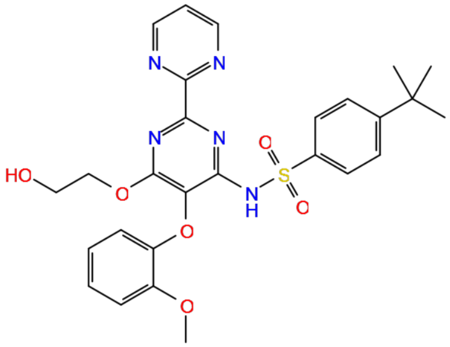

1
Bosentan

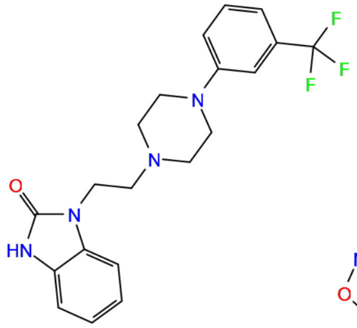

2
Flibanserin

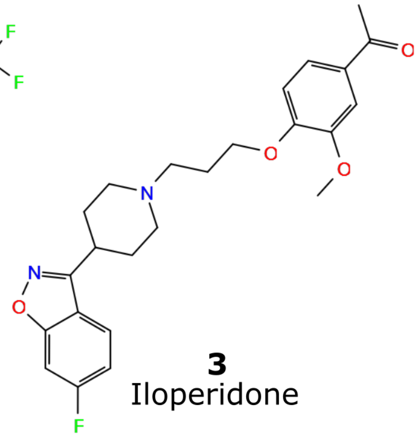

3
Iloperidone

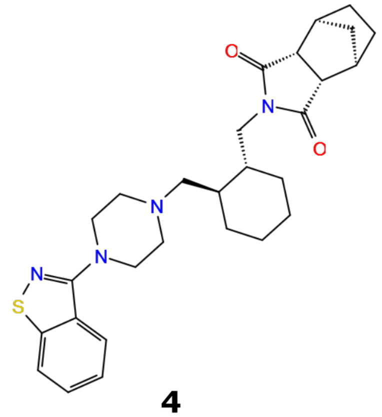

4
Lurasidone

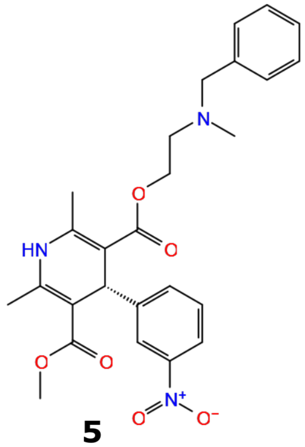

5
Nicardipine

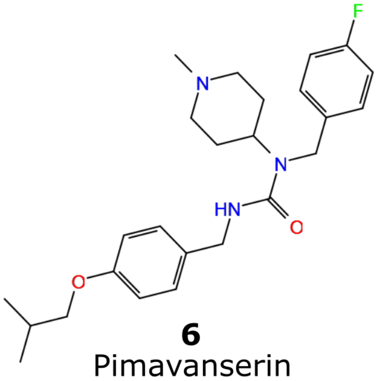

6
Pimavanserin

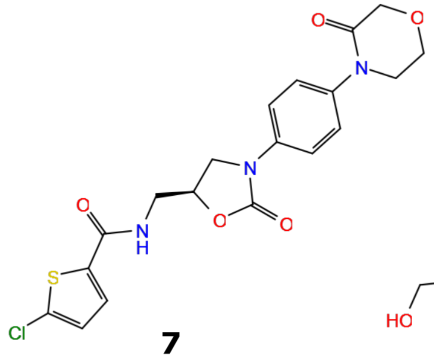

7
Rivaroxaban

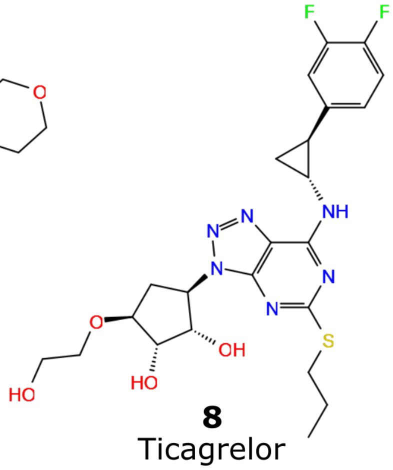

8
Ticagrelor

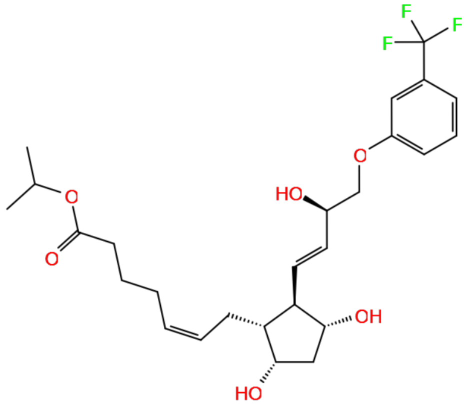

9
Travoprost

Figure S2: Structural formulas of the drugs from the first iteration selected for the in vitro binding assay.

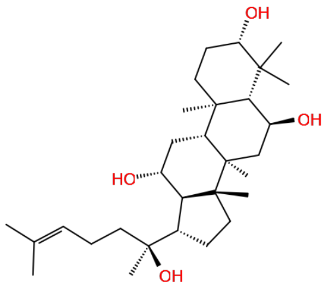

10
(20S)-Protopanaxatriol

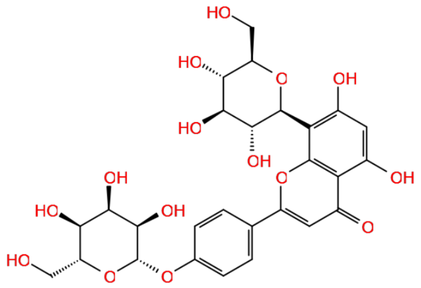

11
4''-O-Glucosylvitexin

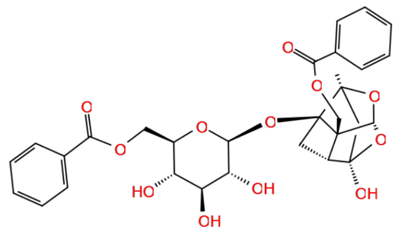

12
Benzoylpaeoniflorin

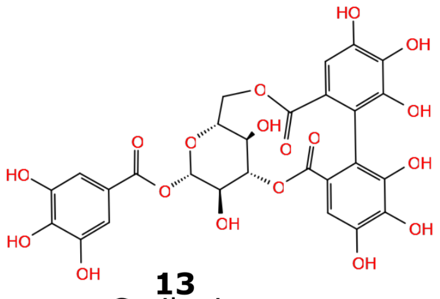

13
Corilagin

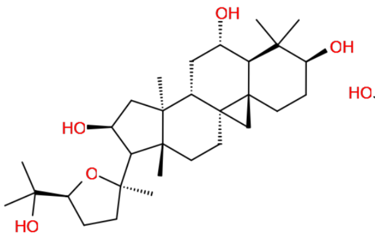

14
Cyclogalegenol

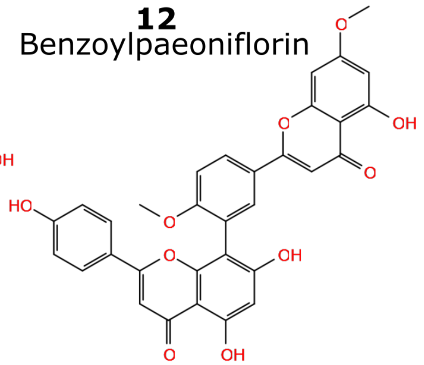

15
Ginkgetin

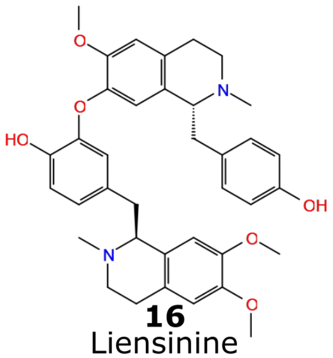

16
Liensinine

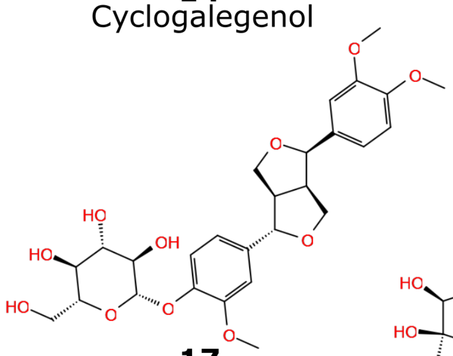

17
Phillyrin

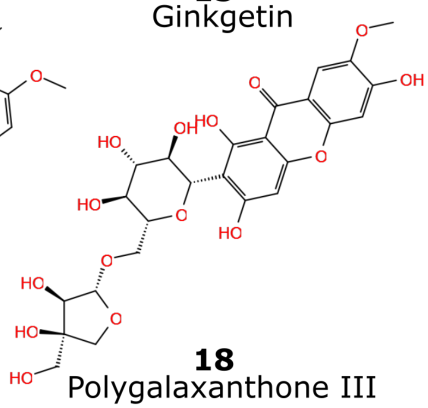

18
Polygalaxanthone III

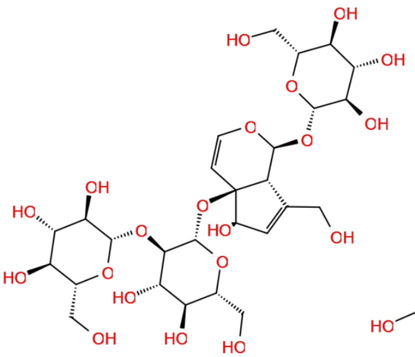

19
Rehmannioside D

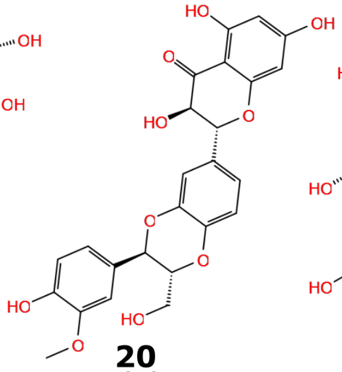

20
Silybin

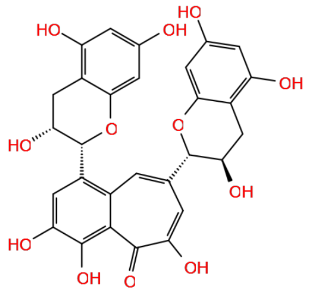

21
Theaflavin

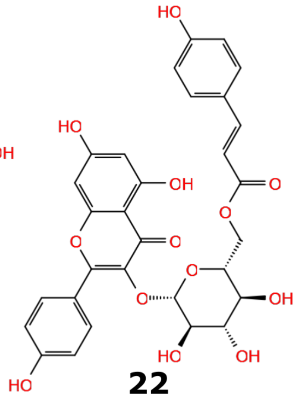

22
Tiliroside

Figure S3: Structural formulas of the phytochemical compounds from the first iteration selected for the in vitro binding assay.

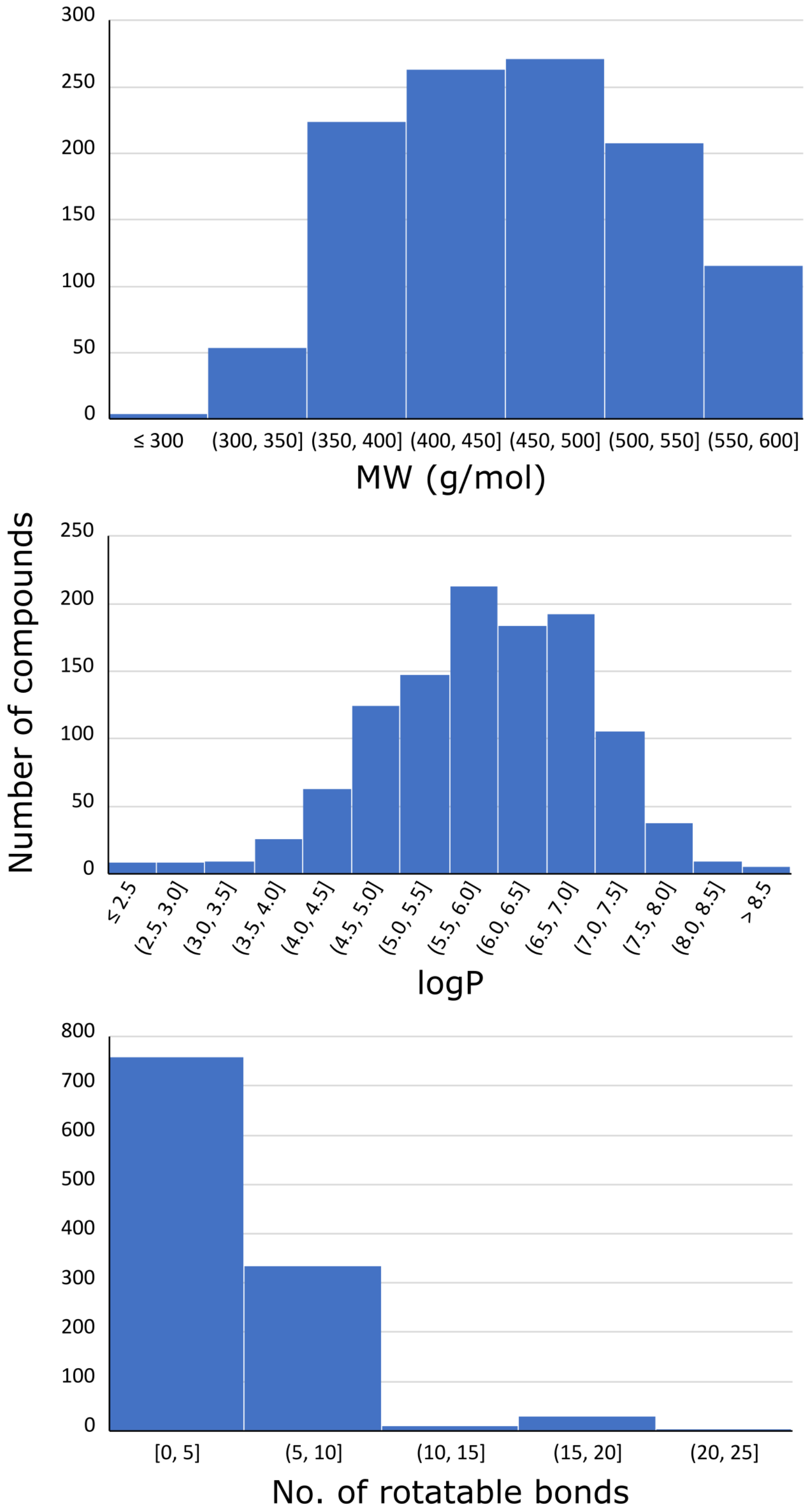

Figure S4: Histograms of MW and computed logP and number of rotatable bonds of CB1 ligands with $K_i \leq 100$ nM deposited in ChEMBL.

Table S2: Statistical parameters of the prepared QSAR models.

Model code	Score	R^2	Q^2	SD	RMSE
kpls_linear_9	0.5690	0.7366	0.6366	0.5881	0.6931
kpls_linear_7	0.5620	0.7299	0.6254	0.5967	0.7000
kpls_dendritic_7	0.5572	0.7442	0.6282	0.5806	0.6974
kpls_linear_2	0.5495	0.7399	0.6189	0.5861	0.7038
kpls_dendritic_9	0.5458	0.8139	0.6541	0.4945	0.6762
kpls_linear_8	0.5458	0.8113	0.6471	0.4993	0.6774
kpls_linear_19	0.5450	0.7395	0.6192	0.5856	0.7068
kpls_linear_4	0.5446	0.8142	0.6504	0.4948	0.6769
kpls_linear_29	0.5444	0.6712	0.5907	0.6579	0.7323
kpls_dendritic_2	0.5442	0.7485	0.6188	0.5763	0.7038

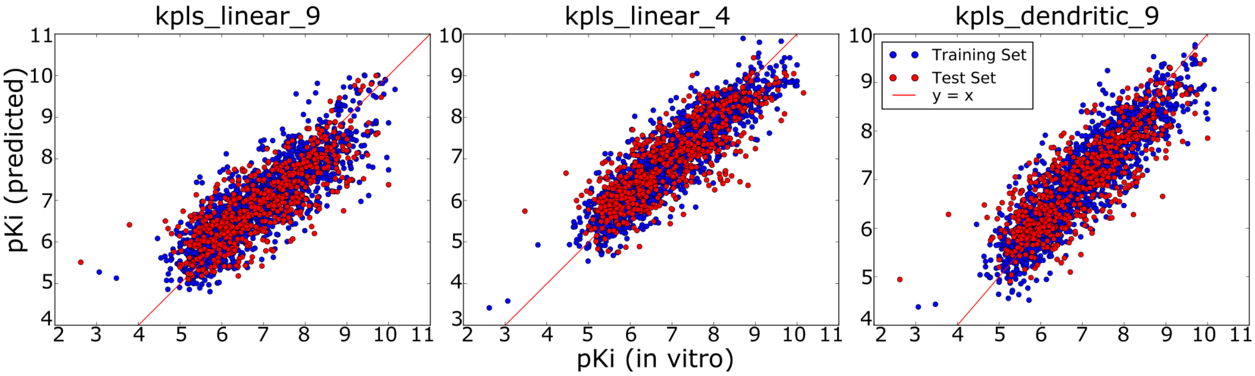

Figure S5: Scatter plots showing correlation of QSAR-predicted and in vitro pK_i values for training and test sets for the best QSAR models based on AutoQSAR score (kpls_linear_9), R^2 (kpls_linear_4), and Q^2 (kpls_dendritic_9).

Table S3: Cutoffs for the physicochemical properties used for separate filtration of potential CB1 agonists and antagonists/inverse agonists.

Property	Agonists	Antagonists
MW (g/mol)	≤ 500	≤ 600
logP	3.5–8	3–8
HBD	≤ 5	≤ 5
HBA	≤ 10	≤ 10
PSA (Å^2)	≤ 140	≤ 140
No. of rotatable bonds	≤ 20	≤ 10

Table S4: Computed physicochemical properties of the compounds from the second iteration VS selected for in vitro binding assay.

ID	ZINC ID	MW (g/mol)	logP	HBD	HBA	No. of rotatable bonds	PSA (Å^2)
23	ZINC000002117717	427.45	6.5	2	4	5	59.0
24	ZINC000005158610	424.49	3.8	3	5	9	117.3
25	ZINC000008790881	443.50	3.9	2	7	8	100.6
26	ZINC000014690613	422.48	4.5	2	5	6	102.5
27	ZINC000015953923	489.61	5.2	1	7	6	88.6
28	ZINC000021362315	457.53	5.7	2	6	8	97.5
29	ZINC000052509366	489.92	4.7	1	7	7	101.2
30	ZINC000217658088	495.53	5.2	3	6	8	110.5
31	ZINC000217830653	482.85	3.9	1	6	3	120.9
32	ZINC000824654462	444.49	4.1	3	6	8	97.2
33	ZINC000081139092	436.53	4.3	1	6	7	32.5
34	ZINC000001832514	438.01	7.6	1	3	10	26.4
35	ZINC000002119702	520.41	6.3	2	5	7	74.6
36	ZINC000002149301	542.61	4.6	1	9	7	135.9
37	ZINC000003033194	509.99	6.9	1	6	8	74.3
38	ZINC000006040794	383.45	6.1	1	3	6	45.9
39	ZINC000013629850	497.65	5.7	1	6	8	83.1
40	ZINC000020237143	523.56	6.5	1	7	7	100.5
41	ZINC000020904533	458.51	4.5	1	8	7	82.7
42	ZINC000036062145	440.54	4.2	1	8	8	84.1
43	ZINC000263585252	490.60	6.3	1	5	9	82.6
44	ZINC000000538337	440.95	5.8	1	4	3	51.0
45	ZINC000012503187	498.58	6.0	2	7	4	83.6

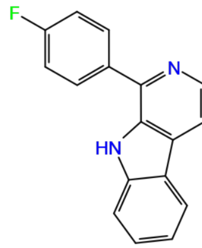

23

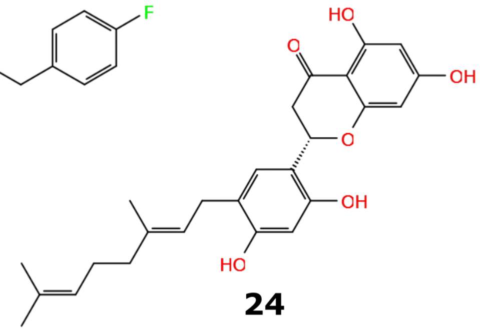

24

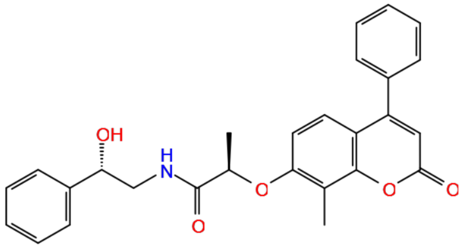

25

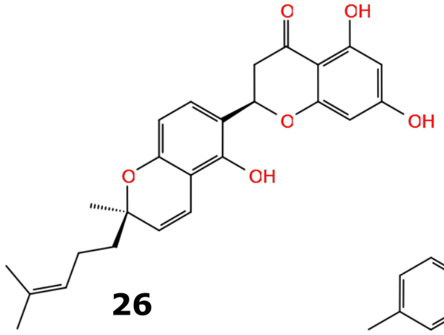

26

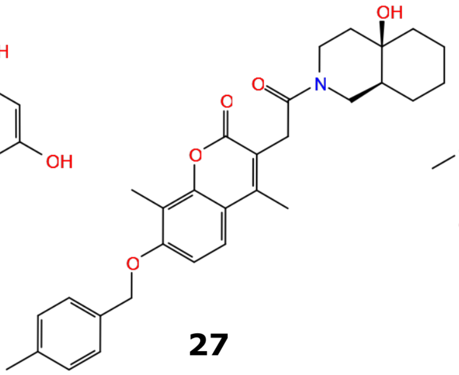

27

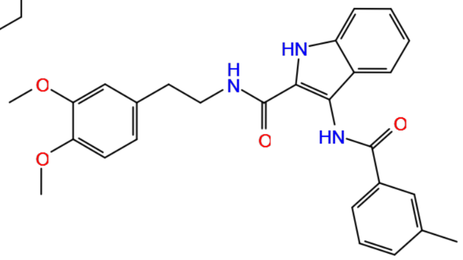

28

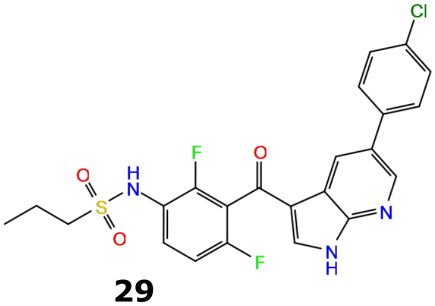

29

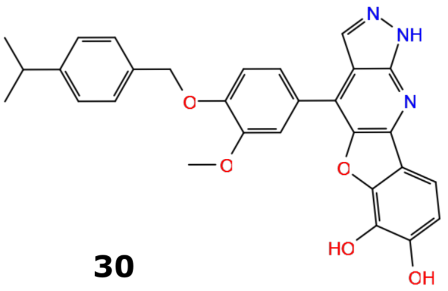

30

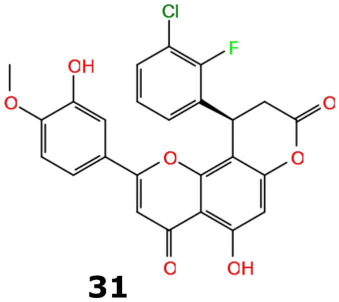

31

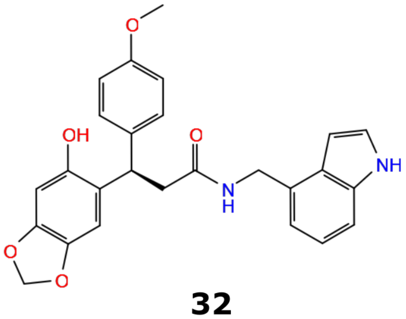

32

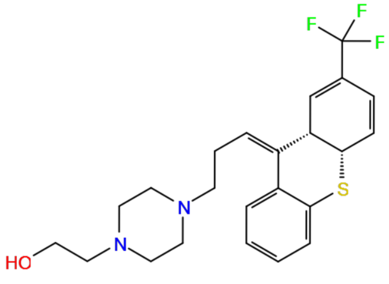

33

Figure S6: Structural formulas of the potential CB1 agonists from the second iteration selected for the in vitro binding assay.

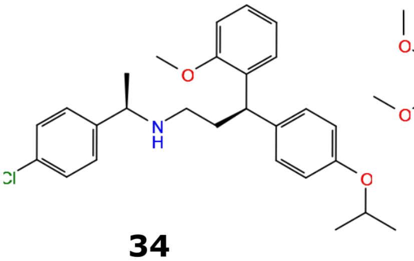

34

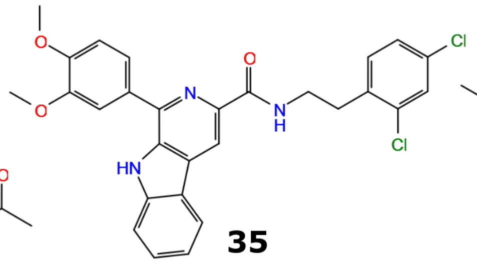

35

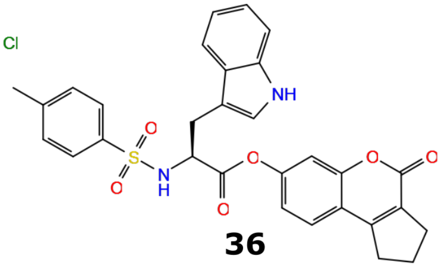

36

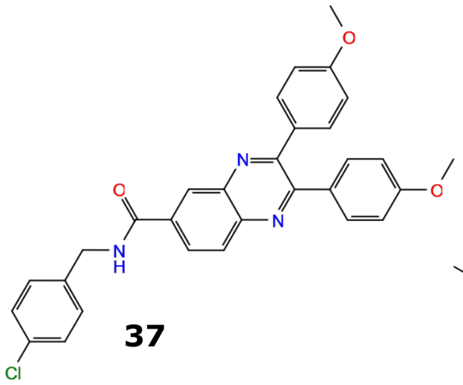

37

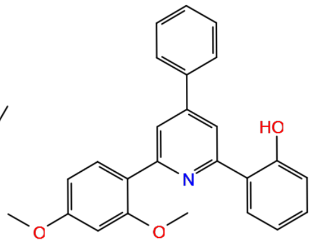

38

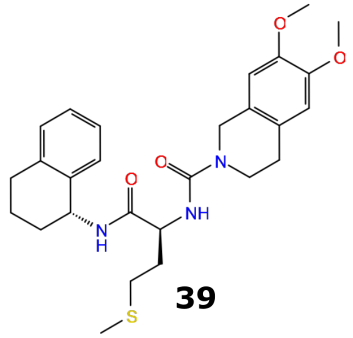

39

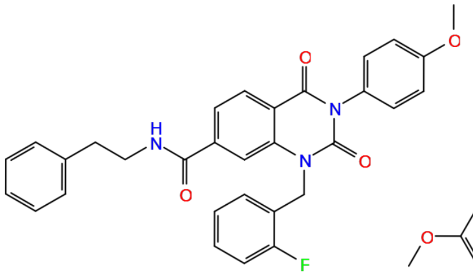

40

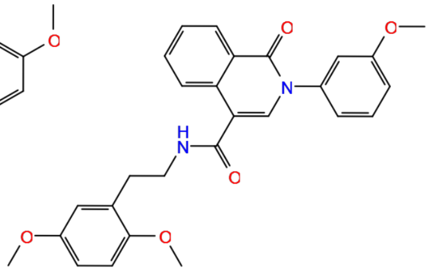

41

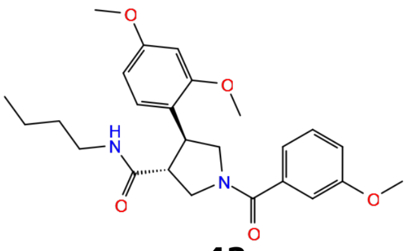

42

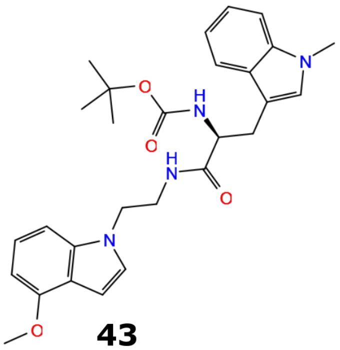

43

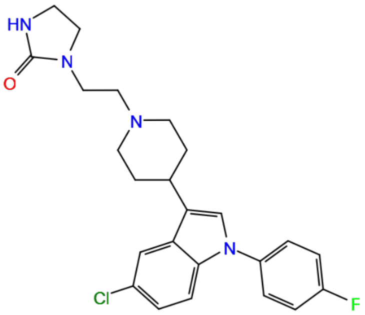

44

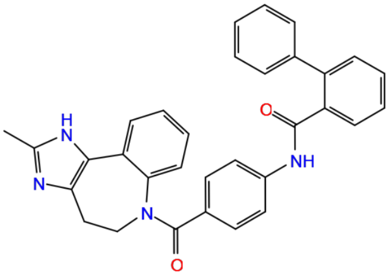

45

Figure S7: Structural formulas of the potential CB1 antagonists from the second iteration selected for the in vitro binding assay.

Table S5: Results of the first iteration K_i determination with [^{3}H]CP-55,940 displacement assay.

**ID**	**ZINC ID**	**Name**	**$pK_i \pm$ SEM**	**K_i (μM, 95% CI)**
1[a]	ZINC000001538857	Bosentan	3.59 ± 0.09	259 (166–403)
2[b]	ZINC000052716421	Flibanserin	3.96 ± 0.08	109 (73.9–160)
3	ZINC000001548097	Iloperidone	4.05 ± 0.12	87.9 (49.8–155.4)
4	ZINC000003927822	Lurasidone	4.40 ± 0.09	39.8 (25.2–63)
5[b,c]	ZINC000019796087	Nicardipine	no activity	no activity
6	ZINC000016159083	Pimavanserin	4.47 ± 0.21	34 (12–93)
7	ZINC000003964126	Rivaroxaban	no activity	no activity
8	ZINC000028957444	Ticagrelor	4.44 ± 0.29	36 (8.8–148)
9	ZINC000004474682	Travoprost	5.40 ± 0.14	3.6 (1.8–7.4)
10	ZINC000043606386	(20S)-Protopanaxatriol	no activity	no activity
11	ZINC000299817443	4"-O-Glucosylvitexin	no activity	no activity
12	ZINC000101324562	Benzoylpaeoniflorin	3.75 ± 0.09	179 (104–307)
13	ZINC000004098612	Corilagin	4.20 ± 0.12	48 (26.8–85.9)
14	ZINC000068248924	Cyclogalegenol	3.76 ± 0.20	175 (64.3–474)
15	ZINC000001531664	Ginkgetin	5.06 ± 0.16	8.6 (3.8–19.2)
16	ZINC000206658454	Liensinine	no activity	no activity
17	ZINC000056874329	Phillyrin	4.20 ± 0.09	62.8 (39.6–99.5)
18	ZINC000085531601	Polygalaxanthone III	no activity	no activity
19	ZINC000253387896	Rehmannioside D	no activity	no activity
20[d]	ZINC000002033589	Silybin	3.78 ± 0.16	165 (76.7–354)
21	ZINC000095850257	Theaflavin	no activity	no activity
22	ZINC000017654711	Tiliroside	4.33 ± 0.11	47.2 (26.5–84.1)
		Rimonabant (reference)	8.30 ± 0.13	0.0043 (0.0023–0.0088)

[a] Monohydrate; [b] hydrochloride; [c] racemic mixture; [d] mixture A and B.

Table S6: Results of the second iteration K_i determination with $[^3H]$CP-55,940 displacement assay.

ID	ZINC ID	Name	$pK_i \pm$ SEM	K_i (μM, 95% CI)
23[a]	ZINC000002117717		no activity	no activity
24	ZINC000005158610	Kuwanon E	4.51 ± 0.08	31 (21–45)
25[a]	ZINC000008790881		4.86 ± 0.09	13.9 (8.7–22)
26	ZINC000014690613	Sanggenon N	4.71 ± 0.05	19 (15–24)
27[a]	ZINC000015953923		insoluble	insoluble
28[a]	ZINC000021362315		no activity	no activity
29	ZINC000052509366	Vemurafenib	no activity	no activity
30	ZINC000217658088		6.07 ± 0.09	0.8 (0.6–1.1)
31[a]	ZINC000217830653		no activity	no activity
32[a]	ZINC000824654462		5.20 ± 0.10	6.8 (3.8–10)
33[b]	ZINC000081139092	Flupentixol	4.27 ± 0.04	53 (44 – 65)
34[a]	ZINC000001832514		5.57 ± 0.07	2.7 (1.9–3.7)
35[a]	ZINC000002119702		4.35 ± 0.09	44 (28–69)
36[a]	ZINC000002149301		4.39 ± 0.09	41 (27–62)
37	ZINC000003033194		no activity	no activity
38	ZINC000006040794		5.60 ± 0.09	2.5 (1.5–4.1)
39[a]	ZINC000013629850		4.40 ± 0.07	40 (29 – 56)
40	ZINC000020237143		no activity	no activity
41	ZINC000020904533		no activity	no activity
42[a]	ZINC000036062145		no activity	no activity
43	ZINC000263585252		5.18 ± 0.07	6.6 (4.7–9.4)
44	ZINC000000538337	Sertindole	4.38 ± 0.05	42 (32–54)
45	ZINC000012503187	Conivaptan	no activity	no activity
		Rimonabant (reference)	8.30 ± 0.13	0.0043 (0.0023–0.0088)

[a] Racemic mixture; [b] dihydrochloride.

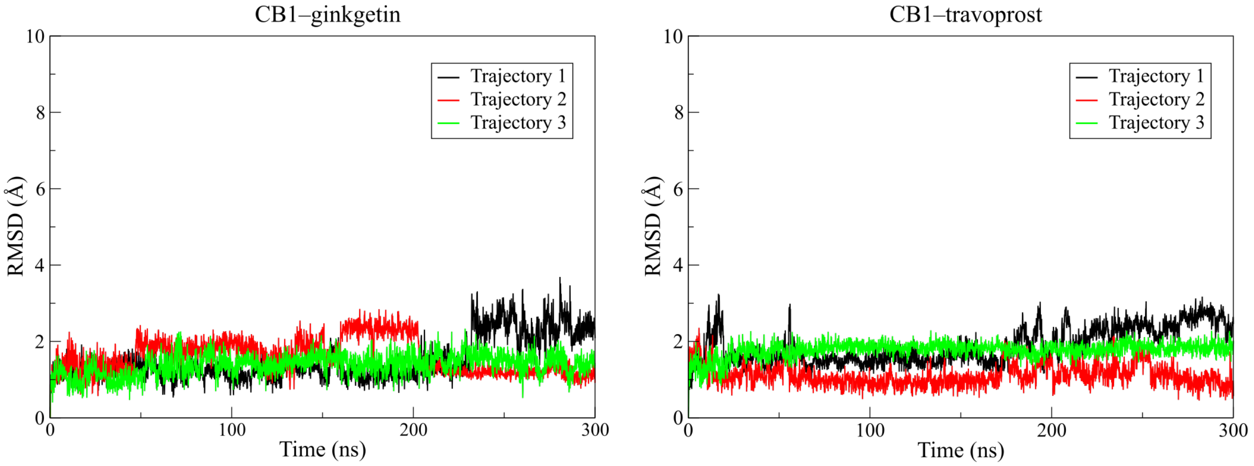

Figure S8: RMSD plots of ligands' heavy atoms from MD simulations of CB1–ginkgetin and CB1–travoprost complexes. RMSD computed after superposition on C_α atoms with the exception of ICL3 (residues 307–335).

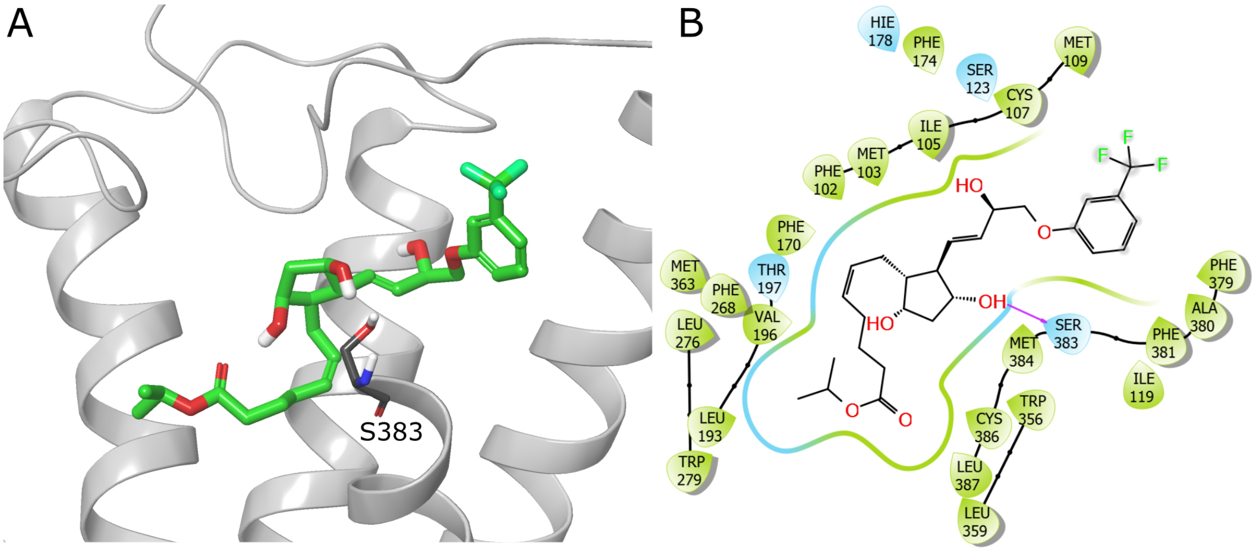

Figure S9: Putative binding mode of compound **9** (ZINC000004474682) from the second iteration. Panels **(A)** and **(B)** depict the pose and 2D interaction scheme obtained from docking to PDB ID: 5TGZ using Glide SP. Ligand is depicted in green stick representation, amino acids that are crucial for ligand binding—grey stick representation. Purple arrow—H-bond. Parts of TM6 and TM7 not shown to increase readability.

Table S7: Results of the second iteration methods applied for compounds selected from the first iteration.

ID	Pred. pK_i	5XR8				5TGZ			
		SP DS	XP DS	SP—constraints DS	XP—constraints DS	SP DS	MM–GBSA ΔG_{bind} (kcal/mol)	SP—constraints DS	MM–GBSA—constraints ΔG_{bind} (kcal/mol)
1	–	–	–	–	–	–	–	–	–
2	−2.1	−8.2	–	−8.8	–	−8.6	–	−7.5	–
3	−2.4	−8.9	–	–	–	−9.0	–	−8.2	–
4	−2.3	–	–	–	–	−8.9	–	−9.3	–
5	−3.2	–	–	–	–	–	–	–	–
6	−2.6	–	–	–	–	–	–	–	–
7	–	–	–	–	–	–	–	–	–
8	–	–	–	–	–	–	–	–	–
9	−2.2	−8.5	–	−10.4	−3.8	−10.3	−83.3	−10.3	−83.4
10	−2.9	–	–	–	–	–	–	–	–
11	–	–	–	–	–	–	–	–	–
12	–	–	–	–	–	–	–	–	–
13	–	–	–	–	–	–	–	–	–
14	−2.9	–	–	–	–	–	–	–	–
15	–	–	–	–	–	–	–	–	–
16	–	–	–	–	–	–	–	–	–
17	–	–	–	–	–	–	–	–	–
18	–	–	–	–	–	–	–	–	–
19	–	–	–	–	–	–	–	–	–
20	–	–	–	–	–	–	–	–	–
21	–	–	–	–	–	–	–	–	–
22	–	–	–	–	–	–	–	–	–

DS, docking score.

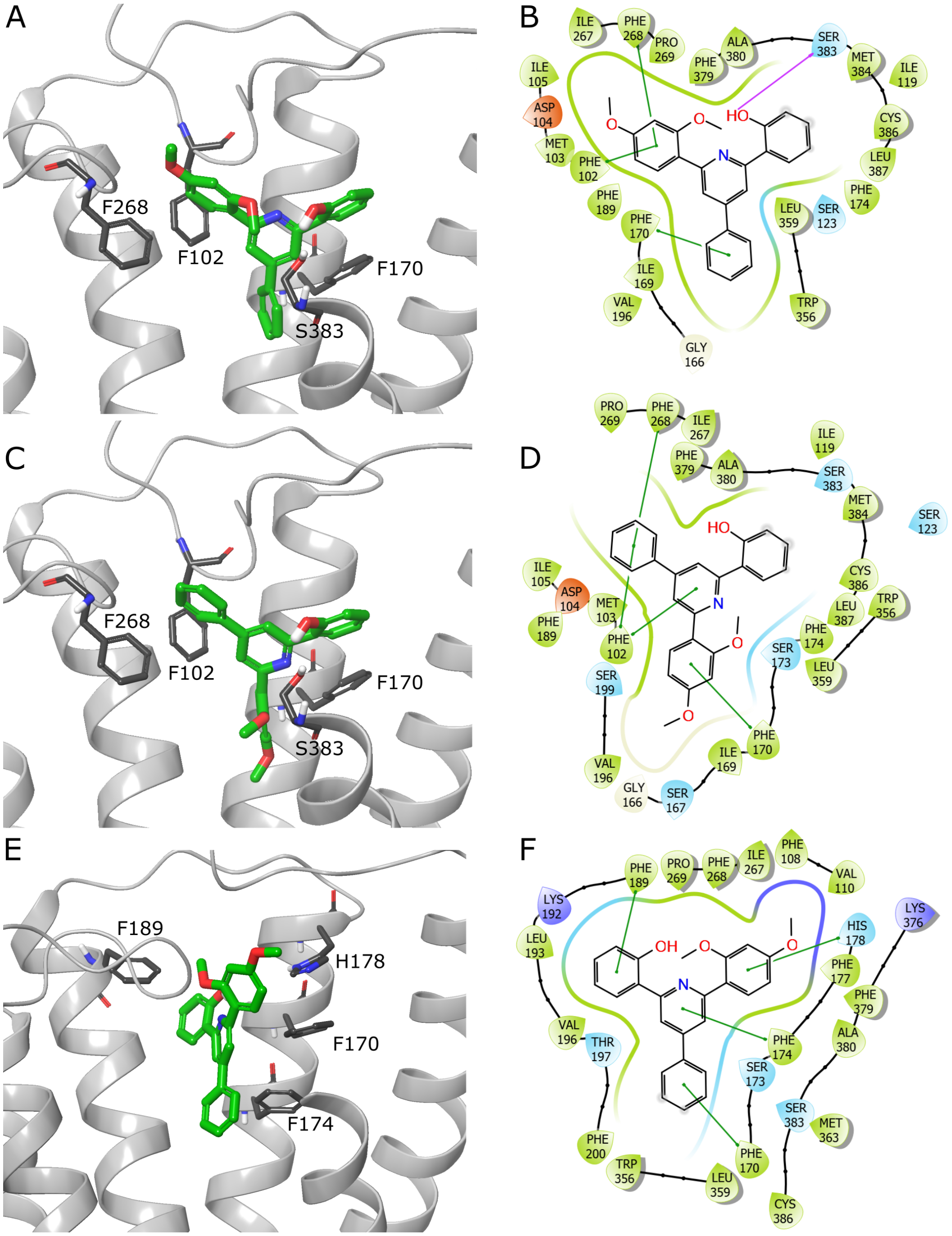

Figure S10: Putative binding modes of compound **38** (ZINC000006040794). Panels **(A)** and **(B)** depict the pose and 2D interaction scheme obtained from docking to PDB ID: 5TGZ using Glide SP, panels **(C)** and **(D)**—docking to PDB ID: 5TGZ using Glide SP with constraints on forming an H-bond with Ser383, while panels **(E)** and **(F)**—docking to PDB ID: 5XR8 using Glide XP. Ligands are depicted in green stick representation, amino acids that are crucial for ligand binding—grey stick representation. Purple arrow—H-bond; green line—π–π interaction. Parts of TM6 and TM7 not shown to increase readability.

Table S8: CB1 agonists with K_i < 100 nM toward human CB1 used as active test compounds in validation of the second iteration docking and MM–GBSA.

**Name/ID**	**K_i (nM)**	**Reference**
ACEA	5.3	(1)
Ajulemic acid	5.7	(2)
AM-11542	0.11	(3)
AM-411	6.8	(4)
AM-841	1.14	(3)
AMG-3	0.32	(5)
Anandamide	70	(6)
AZ-11713908	3.0	(7)
BAY 38-7271	2.91	(8)
BAY 59-3074	48.3	(9)
CP-55,244	1	(10)
CP-55,940	5.2	(11)
EG-2201	22.4	(12)
HU-210	0.06	(13)
JWH-018	9	(12)
JWH-147	11	(14)
JWH-307	7.7	(14)
MDMB-CHMCZCA	5.75	(12)
MDMB-FUBINACA	0.098	(12)
Noladin ether	25	(15)
PSB-SB-1201	22	(16)
UR-144	29	(17)
WIN-55,212-2	13.3	(18)
XLR-11	24	(17)
Δ-9-THC	22	(19)

Table S9: CB1 antagonists/inverse agonists with K_i < 100 nM toward human CB1 used as active test compounds in validation of the second iteration docking and MM–GBSA.

**Name/ID**	**K_i (nM)**	**Reference**
AM-251	7.49	(20)
AM-281	12	(21)
AM-6538	3.4	(22)
AM-6545	1.7	(23)
CHEMBL201602	0.6	(24)
CHEMBL2063237	0.14	(25)
CHEMBL2063246	0.13	(25)
CHEMBL349955	81.5	(20)
CHEMBL482741	1.3	(26)
CHEMBL495891	14.96	(27)
CHEMBL496293	0.91	(27)
CHEMBL498783	1.2	(27)
CHEMBL568221	3.8	(28)
Drinabant	11	(29)
Ibipinabant	7.8	(30)
JD-5037	0.35	(30)
MRI-1867	2.3	(31)
NESS-0327	0.00035	(32)
O-2050	2.5	(33)
Otenabant	0.7	(34)
PF-0514273	1	(26)
PipISB	1.5	(35)
Rimonabant	6	(36)
Surinabant	3.5	(37)
Taranabant	0.13	(38)

Table S10: Statistical parameters from the validation of docking and MM–GBSA protocols selected for the second iteration of VS.

**PDB ID**	**Method**	**$EF_{1\%}$**	**$EF_{2\%}$**	**$EF_{5\%}$**	**BEDROC α = 160.9**	**BEDROC α = 20.0**
5XR8	Glide SP	24.0	18.0	7.2	0.568	0.420
5XR8	Glide XP	32.0	20.0	9.6	0.551	0.523
5XR8	Glide SP, constraints	28.0	16.0	6.4	0.628	0.367
5XR8	Glide XP, constraints	32.0	18.0	8.0	0.672	0.426
5TGZ	Glide SP	12.0	14.0	8.8	0.374	0.443
5TGZ	MM–GBSA	32.0	16.0	6.4	0.639	0.415
5TGZ	Glide SP, constraints	32.0	20.0	9.6	0.580	0.487
5TGZ	MM–GBSA, constraints	32.0	16.0	8.0	0.683	0.423

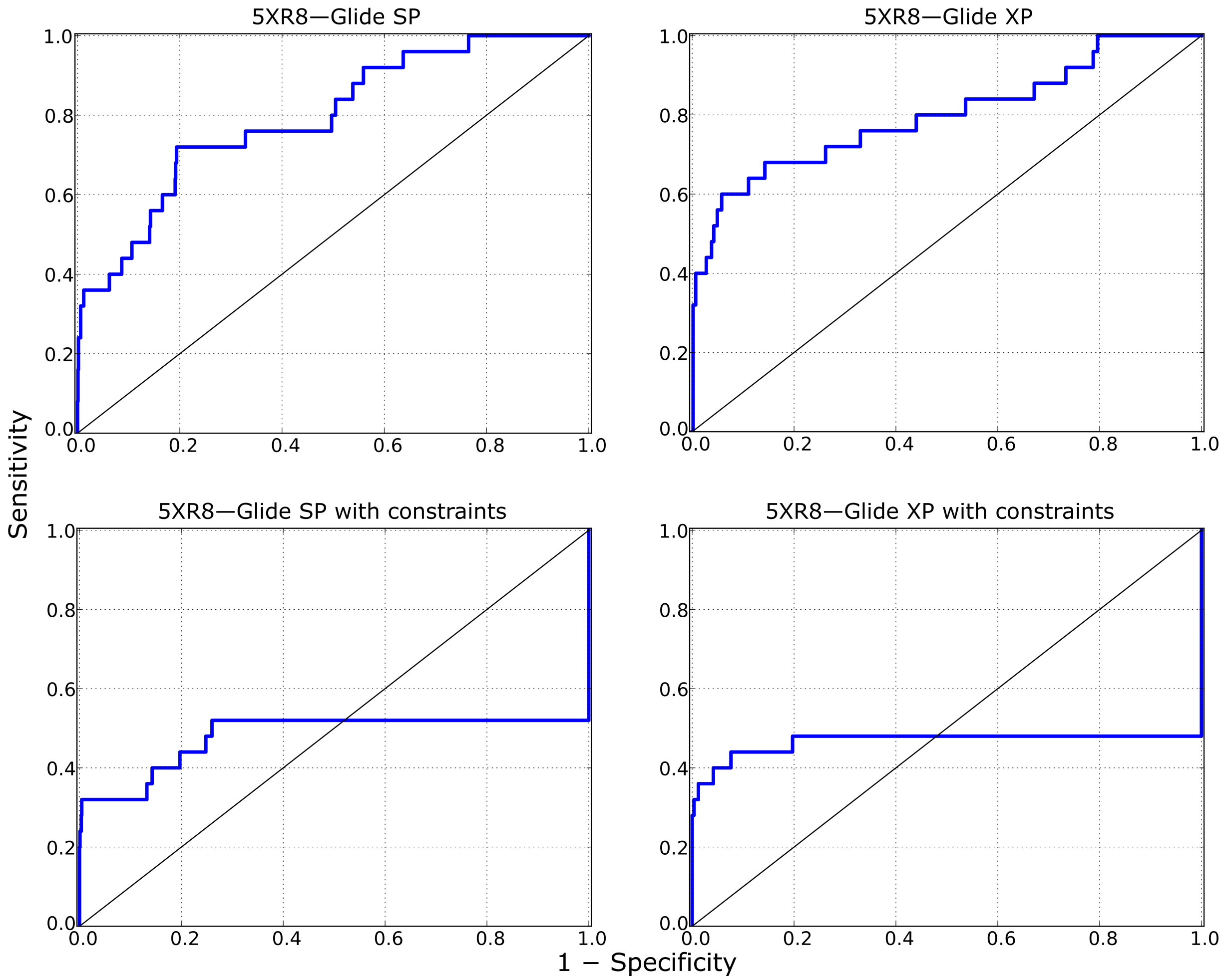

Figure S11: Receiver operating characteristic curves from the validation of docking with Glide to CB1 model based on PDB ID: 5XR8.

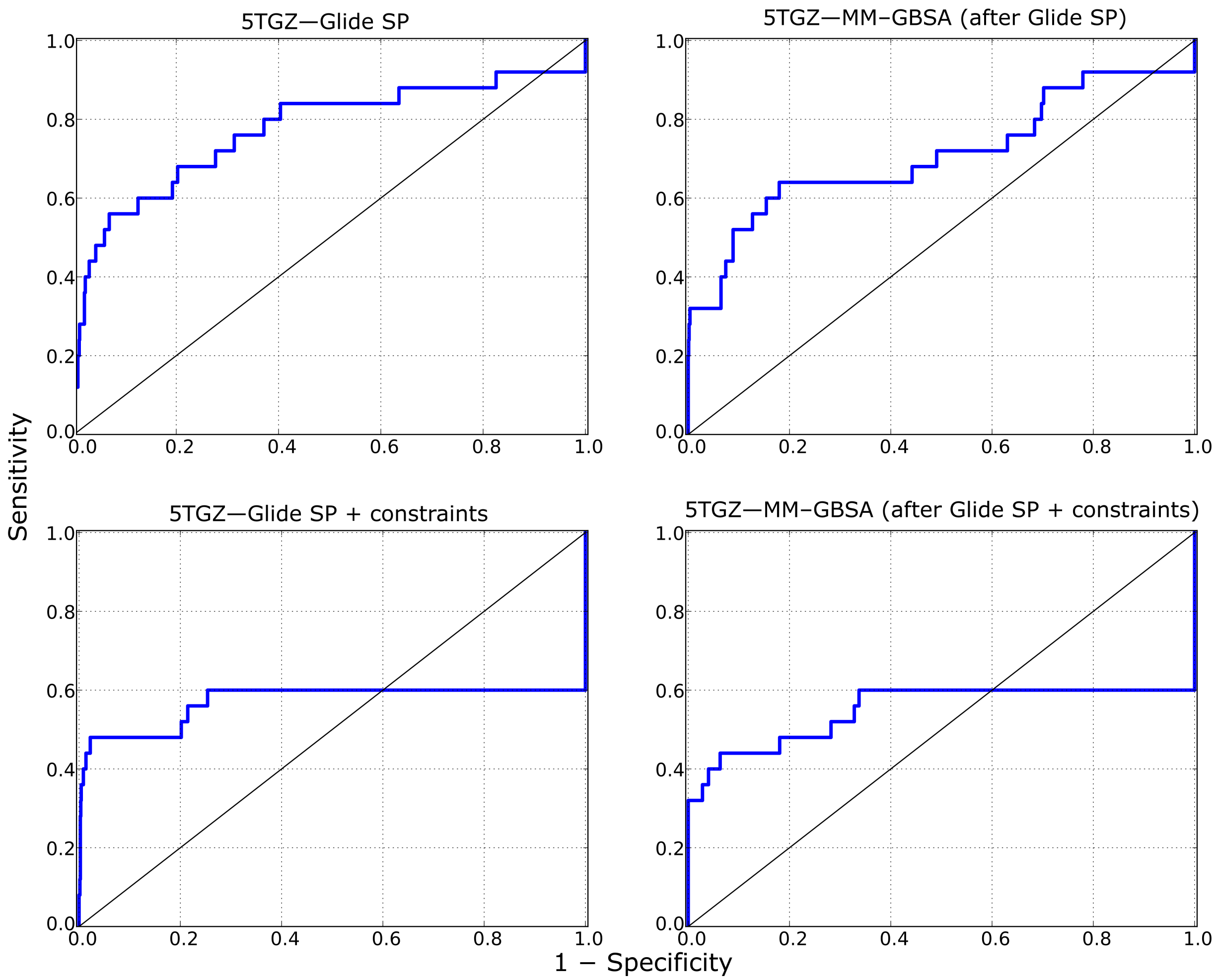

Figure S12: Receiver operating characteristic curves from the validation of docking with Glide to CB1 model based on PDB ID: 5TGZ.

1 Abbreviations Used

BEDROC, Boltzmann-enhanced discrimination of the receiver operating characteristic; CB1, cannabinoid receptor type 1; CI, confidence interval; DS, docking score; EF, enrichment factor; HBA, (number of) hydrogen bond acceptor(s); HBD, (number of) hydrogen bond donor(s); ICL3, intracellular loop 3; K_i, inhibition constant; MD, molecular dynamics; MM–GBSA, molecular mechanics–generalized Born surface area; MW, molecular weight; PDB, Protein Data Bank; PSA, polar surface area; QSAR, quantitative structure—activity relationship; RMSD, root-mean-square deviation; RMSE, root-mean-square error; SD, standard deviation; SEM, standard error of measurement; SP, standard precision; TM, transmembrane (helix); VS, virtual screening; XP, extra precision.

References

(1) Manera, C., Cascio, M. G., Benetti, V., Allarà, M., Tuccinardi, T., Martinelli, A., Saccomanni, G., Vivoli, E., Ghelardini, C., Di Marzo, V., and Ferrarini, P. L. (2007) New 1,8-Naphthyridine and Quinoline Derivatives as CB2 Selective Agonists. *Bioorg. Med. Chem. Lett. 17*, 6505–6510.

(2) Burstein, S. H. (2014) The Cannabinoid Acids, Analogs and Endogenous Counterparts. *Bioorg. Med. Chem. 22*, 2830–2843.

(3) Hua, T., Vemuri, K., Nikas, S. P., Laprairie, R. B., Wu, Y., Qu, L., Pu, M., Korde, A., Jiang, S., Ho, J.-H., Han, G. W., Ding, K., Li, X., Liu, H., Hanson, M. A., Zhao, S., Bohn, L. M., Makriyannis, A., Stevens, R. C., and Liu, Z.-J. (2017) Crystal Structures of Agonist-Bound Human Cannabinoid Receptor CB1. *Nature 547*, 468–471.

(4) Durdagi, S., Kapou, A., Kourouli, T., Andreou, T., Nikas, S. P., Nahmias, V. R., Papahatjis, D. P., Papadopoulos, M. G., and Mavromoustakos, T. (2007) The Application of 3D-QSAR Studies for Novel Cannabinoid Ligands Substituted at the C1' Position

of the Alkyl Side Chain on the Structural Requirements for Binding to Cannabinoid Receptors CB1 and CB2. *J. Med. Chem. 50*, 2875–2885.

(5) Papahatjis, D. P., Kourouli, T., Abadji, V., Goutopoulos, A., and Makriyannis, A. (1998) Pharmacophoric Requirements for Cannabinoid Side Chains: Multiple Bond and C1'-Substituted Δ8-Tetrahydrocannabinols. *J. Med. Chem. 41*, 1195–1200.

(6) Brizzi, A., Cascio, M. G., Frosini, M., Ligresti, A., Aiello, F., Biotti, I., Brizzi, V., Pertwee, R. G., Corelli, F., and Di Marzo, V. (2011) Resorcinol-sn-glycerol Derivatives: Novel 2-Arachidonoylglycerol Mimetics Endowed with High Affinity and Selectivity for Cannabinoid Type 1 Receptor. *J. Med. Chem. 54*, 8278–8288.

(7) Cheng, Y.-X., Pourashraf, M., Luo, X., Srivastava, S., Walpole, C., Salois, D., St-Onge, S., Payza, K., Lessard, E., Yu, X. H., and Tomaszewski, M. J. (2012) γ-Carbolines: A Novel Class of Cannabinoid Agonists with High Aqueous Solubility and Restricted CNS Penetration. *Bioorg. Med. Chem. Lett. 22*, 1619–1624.

(8) Mauler, F., Mittendorf, J., Horváth, E., and De Vry, J. (2002) Characterization of the Diarylether Sulfonylester (-)-(R)-3-(2-hydroxymethylindanyl-4-oxy)phenyl-4,4,4-trifluoro-1-sulfonate (BAY 38-7271) as a Potent Cannabinoid Receptor Agonist with Neuroprotective Properties. *J. Pharmacol. Exp. Ther. 302*, 359–368.

(9) De Vry, J., Denzer, D., Reissmueller, E., Eijckenboom, M., Heil, M., Meier, H., and Mauler, F. (2004) 3-[2-cyano-3-(trifluoromethyl)phenoxy]phenyl-4,4,4-trifluoro-1-butanesulfonate (BAY 59-3074): A Novel Cannabinoid Cb1/Cb2 Receptor Partial Agonist with Antihyperalgesic and Antiallodynic Effects. *J. Pharmacol. Exp. Ther. 310*, 620–632.

(10) Govaerts, S. J., Hermans, E., and Lambert, D. M. (2004) Comparison of Cannabinoid Ligands Affinities and Efficacies in Murine Tissues and in Transfected Cells Expressing Human Recombinant Cannabinoid Receptors. *Eur. J. Pharm. Sci. 23*, 233–243.

(11) Kanyonyo, M., Govaerts, S. J., Hermans, E., Poupaert, J. H., and Lambert, D. M. (1999) 3-Alkyl-(5,5-diphenyl)imidazolidinediones as New Cannabinoid Receptor Ligands. *Bioorg. Med. Chem. Lett. 9*, 2233–2236.

(12) Schoeder, C. T., Hess, C., Madea, B., Meiler, J., and Müller, C. E. (2018) Pharmacological Evaluation of New Constituents of "Spice": Synthetic Cannabinoids Based on Indole, Indazole, Benzimidazole and Carbazole Scaffolds. *Forensic Toxicol. 36*, 385–403.

(13) Pertwee, R. G. (1997) Pharmacology of Cannabinoid CB1 and CB2 Receptors. *Pharmacol. Ther. 74*, 129–180.

(14) Huffman, J. W., Padgett, L. W., Isherwood, M. L., Wiley, J. L., and Martin, B. R. (2006) 1-Alkyl-2-aryl-4-(1-naphthoyl) pyrroles: New High Affinity Ligands for the Cannabinoid CB1 and CB2 Receptors. *Bioorg. Med. Chem. Lett. 16*, 5432–5435.

(15) Martin-Couce, L., Martin-Fontecha, M., Capolicchio, S., Lopez-Rodriguez, M. L., and Ortega-Gutierrez, S. (2011) Development of Endocannabinoid-Based Chemical Probes for the Study of Cannabinoid Receptors. *J. Med. Chem. 54*, 5265–5269.

(16) Rempel, V., Volz, N., Hinz, S., Karcz, T., Meliciani, I., Nieger, M., Wenzel, W., Brase, S., and Muller, C. E. (2012) 7-Alkyl-3-benzylcoumarins: A Versatile Scaffold for the Development of Potent and Selective Cannabinoid Receptor Agonists and Antagonists. *J. Med. Chem. 55*, 7967–7977.

(17) Banister, S. D., Stuart, J., Kevin, R. C., Edington, A., Longworth, M., Wilkinson, S. M., Beinat, C., Buchanan, A. S., Hibbs, D. E., Glass, M., Connor, M., McGregor, I. S., and Kassiou, M. (2015) Effects of Bioisosteric Fluorine in Synthetic Cannabinoid Designer Drugs JWH-018, AM-2201, UR-144, XLR-11, PB-22, 5F-PB-22, APICA, and STS-135. *ACS Chem. Neurosci. 6*, 1445–1458.

(18) Frost, J. M., Dart, M. J., Tietje, K. R., Garrison, T. R., Grayson, G. K., Daza, A. V., El-Kouhen, O. F., Miller, L. N., Li, L., Yao, B. B., Hsieh, G. C., Pai, M., Zhu, C. Z.,

Chandran, P., and Meyer, M. D. (2008) Indol-3-yl-tetramethylcyclopropyl Ketones: Effects of Indole Ring Substitution on CB2 Cannabinoid Receptor Activity. *J. Med. Chem.* *51*, 1904–1912.

(19) Rosati, O., Messina, F., Pelosi, A., Curini, M., Petrucci, V., Gertsch, J., and Chicca, A. (2014) One-pot Heterogeneous Synthesis of Δ3-Tetrahydrocannabinol Analogues and Xanthenes Showing Differential Binding to CB1 and CB2 Receptors. *Eur. J. Med. Chem.* *85*, 77–86.

(20) Lan, R., Liu, Q., Fan, P., Lin, S., Fernando, S. R., McCallion, D., Pertwee, R., and Makriyannis, A. (1999) Structure-Activity Relationships of Pyrazole Derivatives as Cannabinoid Receptor Antagonists. *J. Med. Chem.* *42*, 769–776.

(21) Lan, R., Lu, Q., Fan, P., Gatley, J., Volkow, N. D., Fernando, S. R., Pertwee, R., and Makriyannis, A. (1999) Design and Synthesis of the CB1 Selective Cannabinoid Antagonist AM281: A Potential Human SPECT Ligand. *AAPS PharmSci* *1*, 39–45.

(22) Hua, T., Vemuri, K., Pu, M., Qu, L., Han, G. W., Wu, Y., Zhao, S., Shui, W., Li, S., Korde, A., Laprairie, R. B., Stahl, E. L., Ho, J.-H., Zvonok, N., Zhou, H., Kufareva, I., Wu, B., Zhao, Q., Hanson, M. A., Bohn, L. M., Makriyannis, A., Stevens, R. C., and Liu, Z.-J. (2016) Crystal Structure of the Human Cannabinoid Receptor CB1. *Cell* *167*, 750–762.

(23) Randall, P., Vemuri, V., Segovia, K., Torres, E. F., Hosmer, S., Nunes, E., Santerre, J., Makriyannis, A., and Salamone, J. (2010) The Novel Cannabinoid CB1 Antagonist AM6545 Suppresses Food Intake and Food-Reinforced Behavior. *Pharmacol., Biochem. Behav.* *97*, 179–184.

(24) Carpino, P. A., Griffith, D. A., Sakya, S., Dow, R. L., Black, S. C., Hadcock, J. R., Iredale, P. A., Scott, D. O., Fichtner, M. W., Rose, C. R., Day, R., Dibrino, J., Butler, M., Debartolo, D. B., Dutcher, D., Gautreau, D., Lizano, J. S., O'connor, R. E.,

Sands, M. A., Kelly-Sullivan, D., and Ward, K. M. (2006) New Bicyclic Cannabinoid Receptor-1 (CB1-R) Antagonists. *Bioorg. Med. Chem. Lett. 16*, 731–736.

(25) Dow, R. L., Carpino, P. A., Gautreau, D., Hadcock, J. R., Iredale, P. A., Kelly-Sullivan, D., Lizano, J. S., O'Connor, R. E., Schneider, S. R., Scott, D. O., and Ward, K. M. (2012) Design of a Potent CB1 Receptor Antagonist Series: Potential Scaffold for Peripherally-Targeted Agents. *ACS Med. Chem. Lett. 3*, 397–401.

(26) Dow, R. L., Carpino, P. A., Hadcock, J. R., Black, S. C., Iredale, P. A., DaSilva-Jardine, P., Schneider, S. R., Paight, E. S., Griffith, D. A., Scott, D. O., O'Connor, R. E., and Nduaka, C. I. (2009) Discovery of 2-(2-Chlorophenyl)-3-(4-chlorophenyl)-7-(2,2-difluoropropyl)-6,7-dihydro-2H-pyrazolo[3, 4-f][1,4]oxazepin-8(5H)-one (PF-514273), a Novel, Bicyclic Lactam-Based Cannabinoid-1 Receptor Antagonist for the Treatment of Obesity. *J. Med. Chem. 52*, 2652–2655.

(27) Wustrow, D. J., Maynard, G. D., Yuan, J., Zhao, H., Mao, J., Guo, Q., Kershaw, M., Hammer, J., Brodbeck, R. M., Near, K. E., Zhou, D., Beers, D. S., Chenard, B. L., Krause, J. E., and Hutchison, A. J. (2008) Aminopyrazine CB1 Receptor Inverse Agonists. *Bioorg. Med. Chem. Lett. 18*, 3376–3381.

(28) Dow, R. L., Hadcock, J. R., Scott, D. O., Schneider, S. R., Paight, E. S., Iredale, P. A., Carpino, P. A., Griffith, D. A., Hammond, M., and DaSilva-Jardine, P. (2009) Bioisosteric Replacement of the Hydrazide Pharmacophore of the Cannabinoid-1 Receptor Antagonist SR141716A. Part I: Potent, Orally-Active 1,4-Disubstituted Imidazoles. *Bioorg. Med. Chem. Lett. 19*, 5351–5354.

(29) Black, M. D., Stevens, R. J., Rogacki, N., Featherstone, R. E., Senyah, Y., Giardino, O., Borowsky, B., Stemmelin, J., Cohen, C., Pichat, P., Arad, M., Barak, S., De Levie, A., Weiner, I., Griebel, G., and Varty, G. B. (2011) AVE1625, a Cannabinoid CB1 Receptor Antagonist, as a Co-Treatment with Antipsychotics for Schizophrenia: Improvement

in Cognitive Function and Reduction of Antipsychotic-Side Effects in Rodents. *Psychopharmacology 215*, 149–163.

(30) Tam, J., Cinar, R., Liu, J., Godlewski, G., Wesley, D., Jourdan, T., Szanda, G., Mukhopadhyay, B., Chedester, L., Liow, J.-S., Innis, R. B., Cheng, K., Rice, K. C., Deschamps, J. R., Chorvat, R. J., McElroy, J. F., and Kunos, G. (2012) Peripheral Cannabinoid-1 Receptor Inverse Agonism Reduces Obesity by Reversing Leptin Resistance. *Cell Metab. 16*, 167–179.

(31) Cinar, R., Iyer, M. R., Liu, Z., Cao, Z., Jourdan, T., Erdelyi, K., Godlewski, G., Szanda, G., Liu, J., Park, J. K., Mukhopadhyay, B., Rosenberg, A. Z., Liow, J.-S., Lorenz, R. G., Pacher, P., Innis, R. B., and Kunos, G. (2016) Hybrid Inhibitor of Peripheral Cannabinoid-1 Receptors and Inducible Nitric Oxide Synthase Mitigates Liver Fibrosis. *JCI Insight 1*, e87336.

(32) Ruiu, S., Pinna, G. A., Marchese, G., Mussinu, J.-M., Saba, P., Tambaro, S., Casti, P., Vargiu, R., and Pani, L. (2003) Synthesis and Characterization of NESS 0327: A Novel Putative Antagonist of the CB1 Cannabinoid Receptor. *J. Pharmacol. Exp. Ther. 306*, 363–370.

(33) Wiley, J. L., Breivogel, C. S., Mahadevan, A., Pertwee, R. G., Cascio, M. G., Bolognini, D., Huffman, J. W., Walentiny, D. M., Vann, R. E., Razdan, R. K., and Martin, B. R. (2011) Structural and Pharmacological Analysis of O-2050, a Putative Neutral Cannabinoid CB1 Receptor Antagonist. *Eur. J. Pharmacol. 651*, 96–105.

(34) Griffith, D. A., Hadcock, J. R., Black, S. C., Iredale, P. A., Carpino, P. A., DaSilva-Jardine, P., Day, R., DiBrino, J., Dow, R. L., Landis, M. S., O'Connor, R. E., and Scott, D. O. (2009) Discovery of 1-[9-(4-chlorophenyl)-8-(2-chlorophenyl)-9H-purin-6-yl]-4-ethylaminopiperidine-4-carboxylic Acid Amide Hydrochloride (CP-945,598), a

Novel, Potent, and Selective Cannabinoid Type 1 Receptor Antagonist. *J. Med. Chem.* *52*, 234–237.

(35) Donohue, S. R., Halldin, C., Schou, M., Hong, J., Phebus, L., Chernet, E., Hitchcock, S. A., Gardinier, K. M., Ruley, K. M., Krushinski, J. H., Schaus, J., and Pike, V. W. (2008) Radiolabeling of a High Potency Cannabinoid Subtype-1 Receptor Ligand, N-(4-fluoro-benzyl)-4-(3-(piperidin-1-yl)-indole-1-sulfonyl)benzamide (PipISB), with carbon-11 or fluorine-18. *J. Label. Compd. Radiopharm.* *51*, 146–152.

(36) Alig, L., Alsenz, J., Andjelkovic, M., Bendels, S., Bénardeau, A., Bleicher, K., Bourson, A., David-Pierson, P., Guba, W., Hildbrand, S., Kube, D., Lübbers, T., Mayweg, A. V., Narquizian, R., Neidhart, W., Nettekoven, M., Plancher, J.-M., Rocha, C., Rogers-Evans, M., Röver, S., Schneider, G., Taylor, S., and Waldmeier, P. (2008) Benzodioxoles: Novel Cannabinoid-1 Receptor Inverse Agonists for the Treatment of Obesity. *J. Med. Chem.* *51*, 2115–2127.

(37) Lambert, D. M., and Fowler, C. J. (2005) The Endocannabinoid System: Drug Targets, Lead Compounds, and Potential Therapeutic Applications. *J. Med. Chem.* *48*, 5059–5087.

(38) Shao, Z., Yin, J., Chapman, K., Grzemska, M., Clark, L., Wang, J., and Rosenbaum, D. M. (2016) High-Resolution Crystal Structure of the Human CB1 Cannabinoid Receptor. *Nature* *540*, 602–606.
