# Supplementary material for: Identification of CB1 Ligands among Drugs, Phytochemicals and Natural-Like Compounds: Virtual Screening and In Vitro Verification
Source: ACS Chem Neurosci. 2022 Oct 5;13(20):2991–3007. doi: 10.1021/acschemneuro.2c00502 (PMC9585589; doi:10.1021/acschemneuro.2c00502)
Supplement: Supplementary file 3 — cn2c00502_si_003.zip [file cn2c00502_si_003.zip › Purity_identity_files/First iteration/Biopurify/BP0078-COA-PRF20030644.pdf]

成都普瑞法科技开发有限公司
Chengdu Biopurify Phytochemicals Ltd.

文件编码（DOC NO.）：PRF-COA-BP007801

产品分析证书
Certificate of Analysis

中文名称：牡荆素葡萄糖苷

English Name：Vitexin -4''-O-glucoside
别名(Alias):

产品编码(Cat. No.): ! "
CAS Number: 178468-00-3
分子式(M. F.): $C_{27}H_{30}O_{15}$
分子量(M. W.): 594.522

批号(Batch No.): PRF20030644
报告日期(Report date): 2020-03-06

Structure

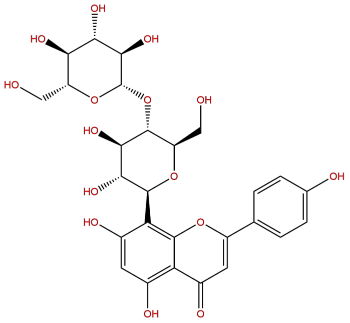

检验结果（Analytical result）：

检验项目（Test Item）	检验指标（Specifications）	检验结果（Results）
外观Appearance	Yellow powder	Yellow powder
干燥失重Loss on drying	＜3.0%	2.1%
纯度Purity (HPLC-DAD,340nm)*	≥98.0%	99.37%
质谱Mass	594.5±1	Conforms
核磁NMR	Comply with the structure	Conforms

*** 色谱图见附件（Please find HPLC chromatogram attached.）**

检测方法（Test Method）： **Column:** Thermo BDS Hypersil C18 4.6*100mm,2.4μm; **Column temperature**: 30℃; **Detection Mode:** UV340nm;

Flow Rate: 1.0ml/min; **Sample dissolution:** Methanol; **Mobile Phase:** A,Acetonitrile **B**, 0.1% Phosphoric acid in water;**Gradient elution**: A,20%-35%,15min.

贮存条件（Storage）:2~8℃, protected from light, keep package airproofed when not in use.

复测期（Retest date）:Three years (2023-03-05) under conditions list above.

QC: Zhang Ling
Date: 2020-03-06

QA: MengPan
Date: 2020-03-06

备注(Remarks):The sample solutions should be prepared and used on the same day, it is the best preparing the solutions immediately before use. If the solutons have to be made up in advance, it should be made as aliquots in tightly sealed vials at less than -20℃. Generally, these might be useable for up to two weeks. The quality of the product is only guaranteed to comply with the attached COA.

In case of quality issue, please contact us within 30 days after receipt of the product.

Tel：+86-28-82633397 Fax：+86-28-82633165
http://www.phytopurify.com Email：sales@biopurify.com biopurify@gmail.com

Chengdu Biopurify

SAMPLE INFORMATION

Sample Name:	Vitexin4Oglucoside	Acquired By:	prf01
Sample Type:	未知	Sample Set Name	
Vial:	111	Acq. Method Set:	Vitexin4″Oglucoside
Injection #:	1	Processing Methoc	sample
Injection Volume:	10.00 ul	Channel Name:	340.0 纳米
Run Time:	30.0 Minutes	Proc. Chnl. Descr.:	PDA 340.0 纳米 (190-600) 纳米
Date Acquired:	2020/3/6 16:22:49 CST		
Date Processed:	2020/3/11 13:35:29 CST		

Auto-Scaled Chromatogram

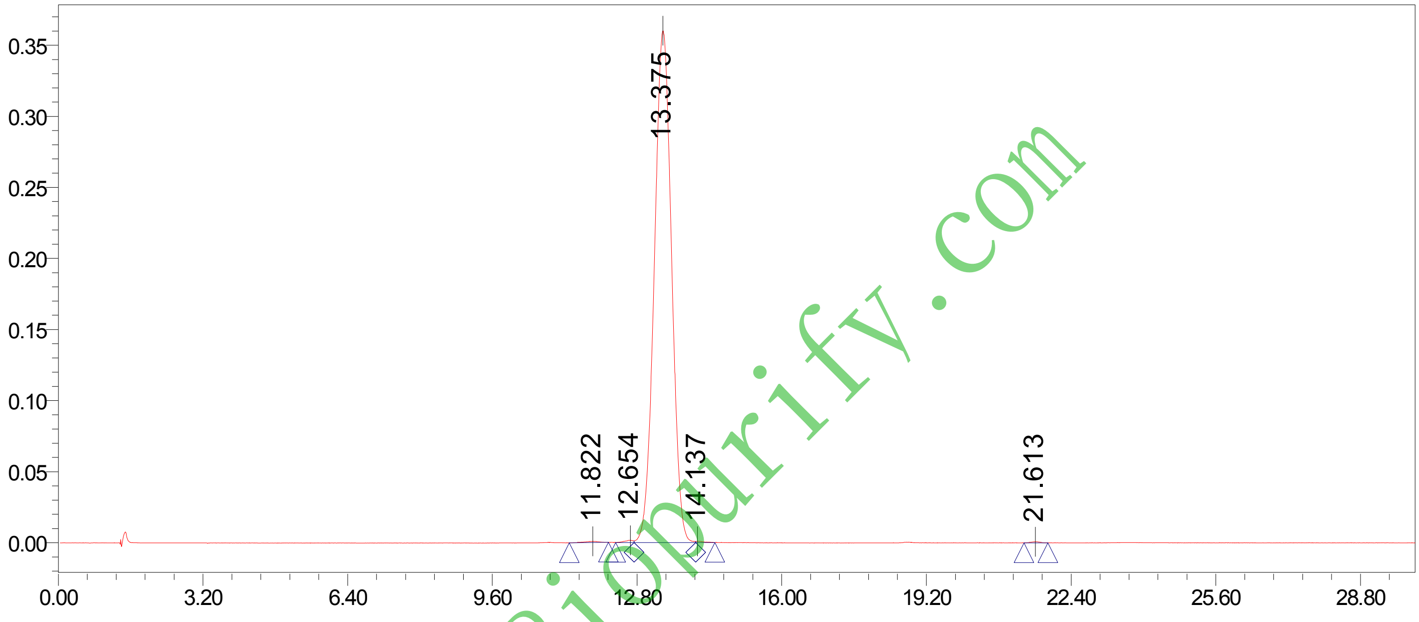

www.Biopurify.com

Peak Results

	RT	Area	% Area	高度 (微伏)	USP 分离度	USP 理论塔板数
1	11.822	18936	0.20	806		4177.14
2	12.654	18601	0.19	1466	1.42	
3	13.375	9509934	99.37	359982	1.11	5810.62
4	14.137	10906	0.11	813	1.07	
5	21.613	11452	0.12	792	13.07	52095.73
