# Supplementary material for: Identification of CB1 Ligands among Drugs, Phytochemicals and Natural-Like Compounds: Virtual Screening and In Vitro Verification
Source: ACS Chem Neurosci. 2022 Oct 5;13(20):2991–3007. doi: 10.1021/acschemneuro.2c00502 (PMC9585589; doi:10.1021/acschemneuro.2c00502)
Supplement: Supplementary file 3 — cn2c00502_si_003.zip [file cn2c00502_si_003.zip › Purity_identity_files/First iteration/Biopurify/BP0393-COA-PRF9101742.pdf]

成都普瑞法科技开发有限公司
Chengdu Biopurify Phytochemicals Ltd.

文件编码（DOC NO.）：PRF-COA-BP039301

产品分析证书
Certificate of Analysis

中文名称：柯里拉京

English Name：Corilagin
别名(Alias):

产品编码(Cat. No.):BP0393
CAS Number: 23094-69-1
分子式(M. F.): C27H22O18
分子量(M. W.): 634.455

批号(Batch No.): PRF9101742
报告日期(Report date): 2018-10-17

Structure

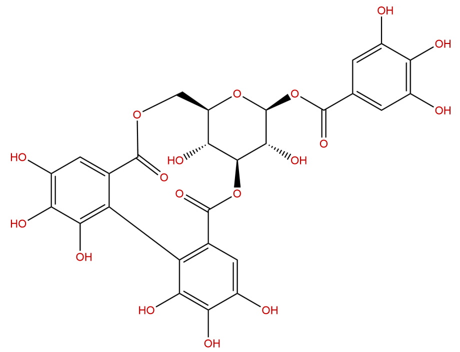

检验结果（Analytical result）:

**检验项目（Test Item）**	**检验指标（Specifications）**	**检验结果（Results）**
外观Appearance	Off-white powder	Off-white powder
干燥失重Loss on drying	＜3.0%	1.34%
纯度Purity (HPLC-DAD,266nm)*	≥98.0%	99.67%
质谱Mass	634.4±1	Conforms
核磁NMR	Comply with the structure	Conforms

*** 色谱图见附件（Please find HPLC chromatogram attached.）**

检测方法（Test Method）：Column: Agilent 5TC-C18,4.6*150mm,3um; **Column temperature**: 30℃; **Detection Mode:** UV266nm

Flow Rate: 1.0ml/min; **Sample dissolution:** Methanol; **Mobile Phase: A,**Acetonitrile **B,** 0.1% Phosphoric acid in water;**Gradient elution: A**, 10%-20%,20min

贮存条件（Storage）:2~8℃, protected from light, keep package airproofed when not in use.

复测期（Retest date）:Three years (2021-10-16) under conditions list above.

QC: Zhang Ling
Date: 2018-10-17

QA: MengPan
Date: 2018-10-17

备注(Remarks):The sample solutions should be prepared and used up on the same day, it is the best preparing the solutions immediately before use. If the solutons have to be made up in advance, it should be made as aliquots in tightly sealed vials at less than -20℃. Generally, these might be useable for up to two weeks. The quality of the product is only guaranteed to comply with the attached COA.
In case of quality issue, please contact us within 30 days after receipt of the product.

成都普瑞法科技开发有限公司 质检专用章

Tel：+86-28-82633397 Fax：+86-28-82633165
http://www.phytopurify.com Email：sales@biopurify.com biopurify@gmail.com

Chengdu Biopurify Phytochemicals Ltd HPLC Report

进样信息

进样名称:	**Corilagin PRF9101742**	*运行时间 (min):*	**30.00**
瓶号:	**BA3**	*进样量:*	**10.00**
进样类型:	未知	*通道:*	**UV_VIS_1**
校正级别:		*波长:*	**266.0**
仪器方法:	**Corilagin**	*带宽:*	**8**
处理方法:	**Corilagin**	*稀释因子:*	**1.0000**
进样日期/时间:	**2018/10/17 11:14**	*样品重量:*	**1.0000**

色谱图

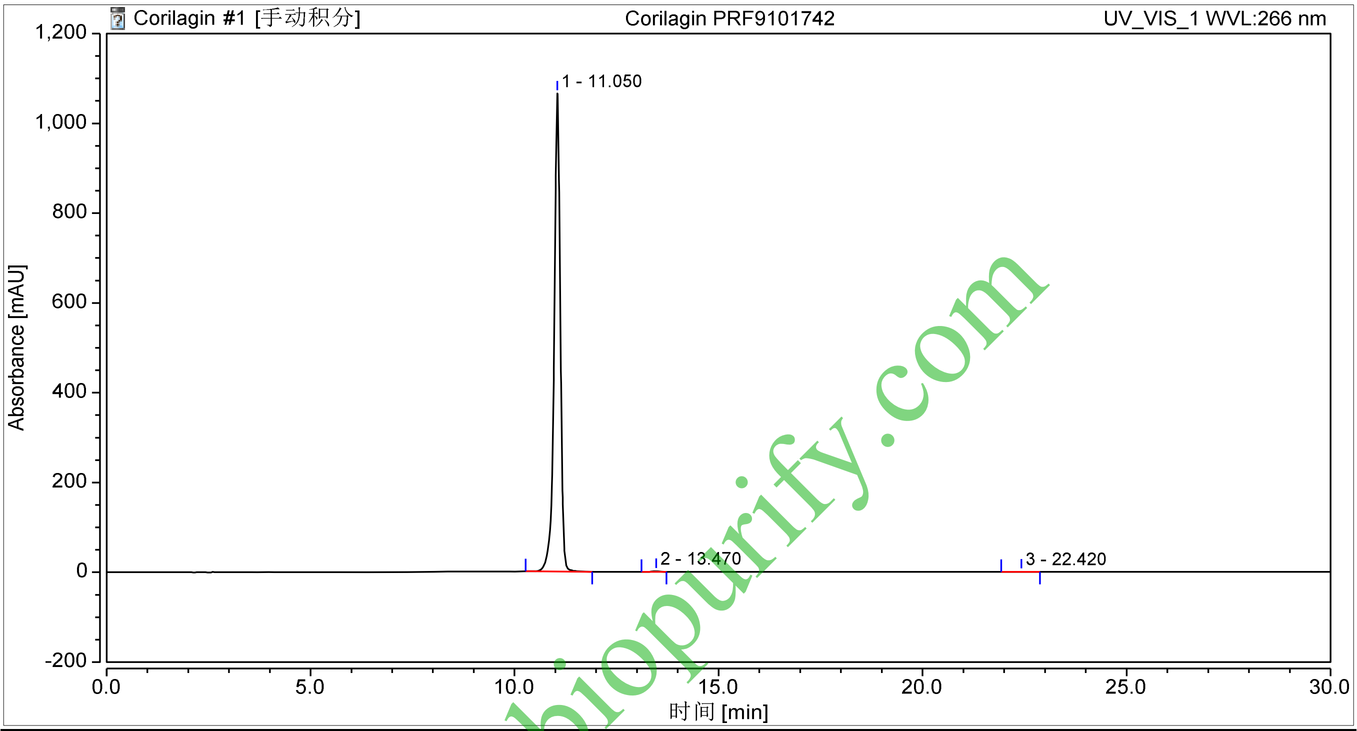

www.biopurify.com

积分结果

序号	保留时间 min	峰面积 mAU*min	峰高 mAU	相对峰面积 %	塔板数 (USP)	分离度 (USP)	不对称度
1	11.050	188.422	1064.974	99.67	27794	9.01	0.87
2	13.470	0.301	1.717	0.16	39145	21.73	0.88
3	22.420	0.326	0.913	0.17	26433	n.a.	0.98
总和:		**189.049**	**1067.605**	**100.00**	**93372.00**		
