# Supplementary material for: Identification of CB1 Ligands among Drugs, Phytochemicals and Natural-Like Compounds: Virtual Screening and In Vitro Verification
Source: ACS Chem Neurosci. 2022 Oct 5;13(20):2991–3007. doi: 10.1021/acschemneuro.2c00502 (PMC9585589; doi:10.1021/acschemneuro.2c00502)
Supplement: Supplementary file 3 — cn2c00502_si_003.zip [file cn2c00502_si_003.zip › Purity_identity_files/First iteration/Biopurify/BP0434-COA-PRF20032322.pdf]

成都普瑞法科技开发有限公司
Chengdu Biopurify Phytochemicals Ltd.

文件编码（DOC NO.）：PRF-COA-BP043401

产品分析证书
Certificate of Analysis

中文名称：环黄芪醇

English Name：Cyclogalegenol
别名(Alias): Astramembrangenin; Cyclosieversigenin; Cyclosiversigenin

产品编码(Cat. No.): BP0434
CAS Number: 78574-94-4
分子式(M. F.): C30H50O5
分子量(M. W.): 490.725

批号(Batch No.): PRF20032322
报告日期(Report date): 2020-03-23

Structure

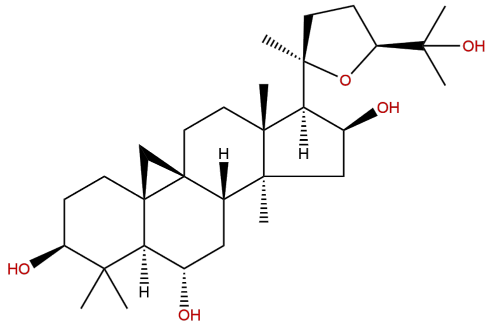

检验结果（Analytical result）：

检验项目（Test Item）	检验指标（Specifications）	检验结果（Results）
外观Appearance	Off-white powder	Off-white powder
干燥失重Loss on drying	＜3.0%	2.1%
纯度Purity (HPLC-ELSD)*	≥98.0%	99.84%
质谱Mass	490.7±1	Conforms
核磁NMR	Comply with the structure	Conforms

*** 色谱图见附件（Please find HPLC chromatogram attached.）**

检测方法（Test Method）： **Column:** Thermo BDS Hypersil C18 4.6*100mm,2.4μm; **Column temperature**: 30℃; **Detection Mode:** ELSD;**Flow Rate:** 1.0ml/min; **Sample dissolution:** Methanol; **Mobile Phase: A**,Acetonitrile **B,** Water;**Gradient elution:** A, 40%-50%,15min.

贮存条件（Storage）:2~8℃, protected from light, keep package airproofed when not in use.

复测期（Retest date）:Three years (2023-03-22) under conditions list above.

QC: Zhang Ling
Date: 2020-03-23

QA: Meng Pan
Date: 2020-03-23

备注(Remarks):The sample solutions should be prepared and used on the same day, it is the best preparing the solutions immediately before use. If the solutons have to be made up in advance, it should be made as aliquots in tightly sealed vials at less than -20℃. Generally, these might be useable for up to two weeks. The quality of the product is only guaranteed to comply with the attached COA.

Chengdu Biopurify Phytochemicals Ltd. 成都普瑞法科技开发有限公司 质检专用章 Quality Approval Seal

In case of quality issue, please contact us within 30 days after receipt of the product.

Tel：+86-28-82633397 Fax：+86-28-82633165
http://www.phytopurify.com Email：sales@biopurify.com biopurify@gmail.com

Empower2 software

Chengdu Biopurify

SAMPLE INFORMATION

Sample Name:	Cycloastragenol PRF20032322	Acquired By:	prf01
Sample Type:	未知	Sample Set Name:	
Vial:	27	Acq. Method Set:	Cycloastragenol
Injection #:	1	Processing Method:	sample
Injection Volume:	10.00 ul	Channel Name:	SATIN
Run Time:	25.0 Minutes	Proc. Chnl. Descr.:	
Date Acquired:	2020-3-23 14:52:58 CST		
Date Processed:	2020-3-23 15:27:12 CST		

Auto-Scaled Chromatogram

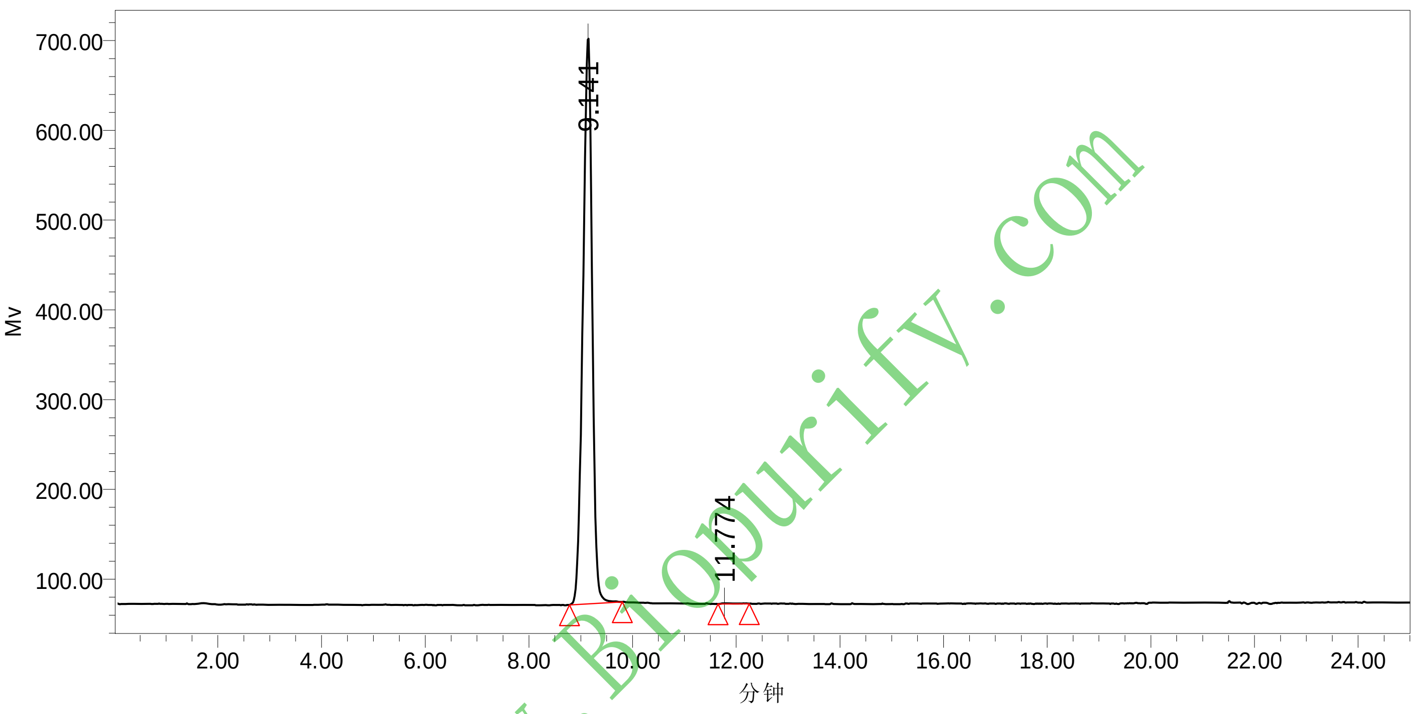

www.Biopurify.com

Peak Results

	Name	RT	Area	% Area	USP Plate Count	USP Resolution
1		9.141	7808578	99.84	12354.28	
2		11.774	12877	0.16	24258.07	6.85
