# Supplementary material for: Identification of CB1 Ligands among Drugs, Phytochemicals and Natural-Like Compounds: Virtual Screening and In Vitro Verification
Source: ACS Chem Neurosci. 2022 Oct 5;13(20):2991–3007. doi: 10.1021/acschemneuro.2c00502 (PMC9585589; doi:10.1021/acschemneuro.2c00502)
Supplement: Supplementary file 3 — cn2c00502_si_003.zip [file cn2c00502_si_003.zip › Purity_identity_files/First iteration/Biopurify/BP0642-COA-PRF9110505.pdf]

# 产品分析证书
## Certificate of Analysis

**中文名称：银杏双黄酮**

**English Name**：Ginkgetin
**别名(Alias):**

**产品编码(Cat. No.):**BP0642
**CAS Number: 481-46-9**
**分子式(M. F.):** C32H22O10
**分子量(M. W.)**: 566.518

**批号(Batch No.):** PRF9110505
**报告日期(Report date):** 2018-11-05

**Structure**

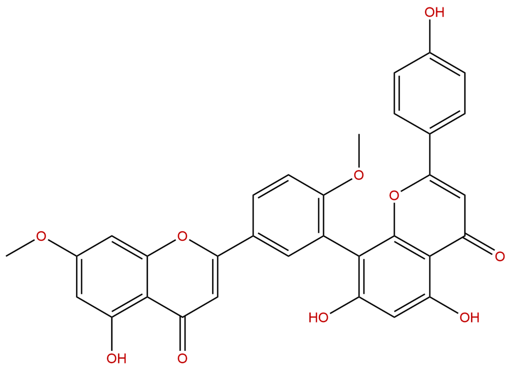

**检验结果（Analytical result）：**

| 检验项目（Test Item） | 检验指标（Specifications） | 检验结果（Results） |
|---|---|---|
| 外观Appearance | Off-white powder | Off-white powder |
| 干燥失重Loss on drying | ＜3.0% | 1.8% |
| 纯度Purity (HPLC-DAD,323nm)* | ≥98.0% | 98.53% |
| 质谱Mass | 566.5±1 | Conforms |
| 核磁NMR | Comply with the structure | Conforms |

*** 色谱图见附件（Please find HPLC chromatogram attached.）**

**检测方法（Test Method）： Column:**Welth Ultimate XB-C18 4.6*150mm,3.0um; **Column temperature**: 30℃; **Detection Mode:** UV323nm

**Flow Rate:** 1.0ml/min; **Sample dissolution:** Methanol; **Mobile Phase: A,**AcetonitrileB**,** 0.1% Phosphoric acid in water;**Gradient elution:** A, 48%-58%,15min

**贮存条件（Storage）**:2~8℃, protected from light, keep package airproofed when not in use.

**复测期（Retest date）**:Three years (2021-11-04) under conditions list above.

QC: Zhang Ling

Date: 2018-11-05

QA: Meng Fan

Date: 2018-11-05

**备注(Remarks)**:The sample solutions should be prepared and used in the same day, it is the best preparing the solutions immediately before use. If the solutons have to be made up in advance, it should be made as aliquots in tightly sealed vials at less than -20℃. Generally, these might be useable for up to two weeks. The quality of the product is only guaranteed to comply with the attached COA.
In case of quality issue, please contact us within 30 days after receipt of the product.

成都普瑞法科技开发有限公司 质检专用章 Chengdu Biopurify Phytochemicals Quality Approval Seal

Tel：+86-28-82633397 Fax：+86-28-82633165
http://www.phytopurify.com Email： sales@biopurify.com biopurify@gmail.com

## SAMPLE INFORMATION

| | | | |
|---|---|---|---|
| Sample Name: | Ginkgetin | Acquired By: | System |
| Sample Type: | Unknown | Sample Set Name: | |
| Vial: | 77 | Acq. Method Set: | 银杏 02 |
| Injection #: | 1 | Processing Method | Samples |
| Injection Volume: | 10 ul | Channel Name: | 323.0nm |
| Run Time: | 20.0 Minutes | Proc. Chnl. Descr.: | PDA 323.0 nm |
| Date Acquired: | 2018-11-3 14:35:13 CST | | |
| Date Processed: | 2018-11-5 13:09:55 CST | | |

### Auto-Scaled Chromatogram

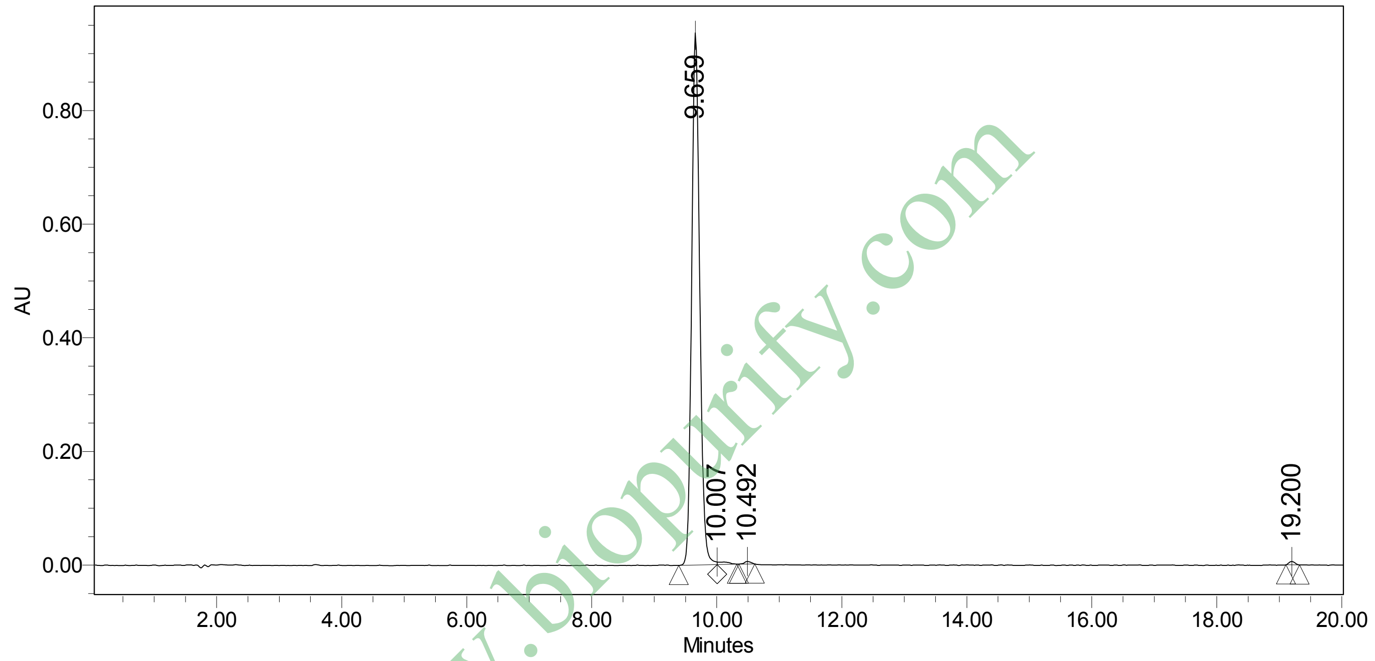


www.biopurify.com

### Peak Results

| | Name | RT | Area | % Area | USP Plate Count | USP Resolution |
|---|---|---|---|---|---|---|
| 1 | | 9.659 | 8377630 | 98.53 | 26890.81 | |
| 2 | | 10.007 | 52847 | 0.62 | | 1.46 |
| 3 | | 10.492 | 32952 | 0.39 | 44284.51 | 2.20 |
| 4 | | 19.200 | 39345 | 0.46 | 186524.89 | 45.59 |
