# Supplementary material for: Identification of CB1 Ligands among Drugs, Phytochemicals and Natural-Like Compounds: Virtual Screening and In Vitro Verification
Source: ACS Chem Neurosci. 2022 Oct 5;13(20):2991–3007. doi: 10.1021/acschemneuro.2c00502 (PMC9585589; doi:10.1021/acschemneuro.2c00502)
Supplement: Supplementary file 3 — cn2c00502_si_003.zip [file cn2c00502_si_003.zip › Purity_identity_files/First iteration/Biopurify/BP0858-COA-PRF20080305.pdf]

成都普瑞法科技开发有限公司
Chengdu Biopurify Phytochemicals Ltd.

文件编码（DOC NO.）：PRF-COA-BP085801

产品分析证书
Certificate of Analysis

中文名称：莲心碱

English Name： Liensinine
别名(Alias): Liensinine diperchlorate, Cas 5088-90-4

产品编码(Cat. No.):BP0858
CAS Number: 2586-96-1
分子式(M. F.): $C_{37}H_{42}N_2O_6$
分子量(M. W.): 610.751

批号(Batch No.): PRF20080305
报告日期(Report date): 2020-08-05

Structure

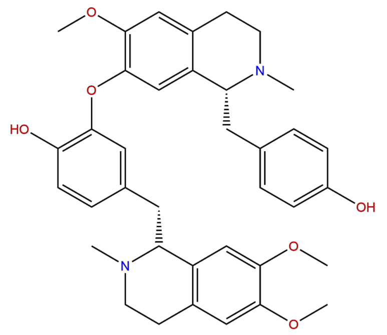

检验结果（Analytical result）：

**检验项目（Test Item）**	**检验指标（Specifications）**	**检验结果（Results）**
外观Appearance	White cotton-like solid	White cotton-like solid
纯度Purity (HPLC-DAD, 283nm)*	≥98.0%	99.19%
质谱Mass	610.7±1	Conforms
核磁NMR	Comply with the structure	Conforms
水分Water Content	＜3.0%	1.32%

* 色谱图见附件（**Please find HPLC chromatogram attached.**）

检测方法（**Test Method**）： **Column:** Heshin DK-H C18,4.6*250mm,5um; **Column temperature**: 30℃; **Detection Mode:** UV283nm; **Flow Rate:** 1.0ml/min; **Sample dissolution:**10% Methanol; **Mobile Phase: A,** Acetonitrile **B,** 50mmol/L Triethylamine in water (Phosphoric acid adjusted to pH 2); **Gradient elution:A,** 10%-20%,15min-20%, 10min.

贮存条件（**Storage**）:2-8℃, protected from light, keep package airproofed when not in use.

复测期（**Retest date**）:Three years (2023-08-03) under conditions list above.

QC: Zhang Ling

Date: 2020-08-04

QA: Meng Pan

Date: 2020-08-04

备注(Remarks):The sample solutions should be prepared and used on the same day. It is the best preparing the solutions immediately before use. If the solutons have to be made up in advance, it should be made as aliquots in tightly sealed vials at less than -20℃. Generally, these might be useable for up to two weeks. The quality of the product is only guaranteed to comply with the attached COA.

In case of quality issue, please contact us within 30 days after receipt of the product.

Tel：+86-28-82633397 Fax：+86-28-82633165
http://www.phytopurify.com Email：sales@biopurify.com biopurify@gmail.com

SAMPLE INFORMATION

Sample Name:	Liensinine	Acquired By:	PRF01
Sample Type:	Unknown	Sample Set Name	
Vial:	40	Acq. Method Set:	Liensinine
Injection #:	1	Processing Method	sample
Injection Volume:	10.00 ul	Channel Name:	PDA Ch1 283nm@4.8nm
Run Time:	25.0 Minutes	Proc. Chnl. Descr.:	PDA Ch1 283nm@4.8nm
Date Acquired:	8/4/2020 10:15:19 AM CST		
Date Processed:	8/5/2020 8:53:37 AM CST		

Auto-Scaled Chromatogram

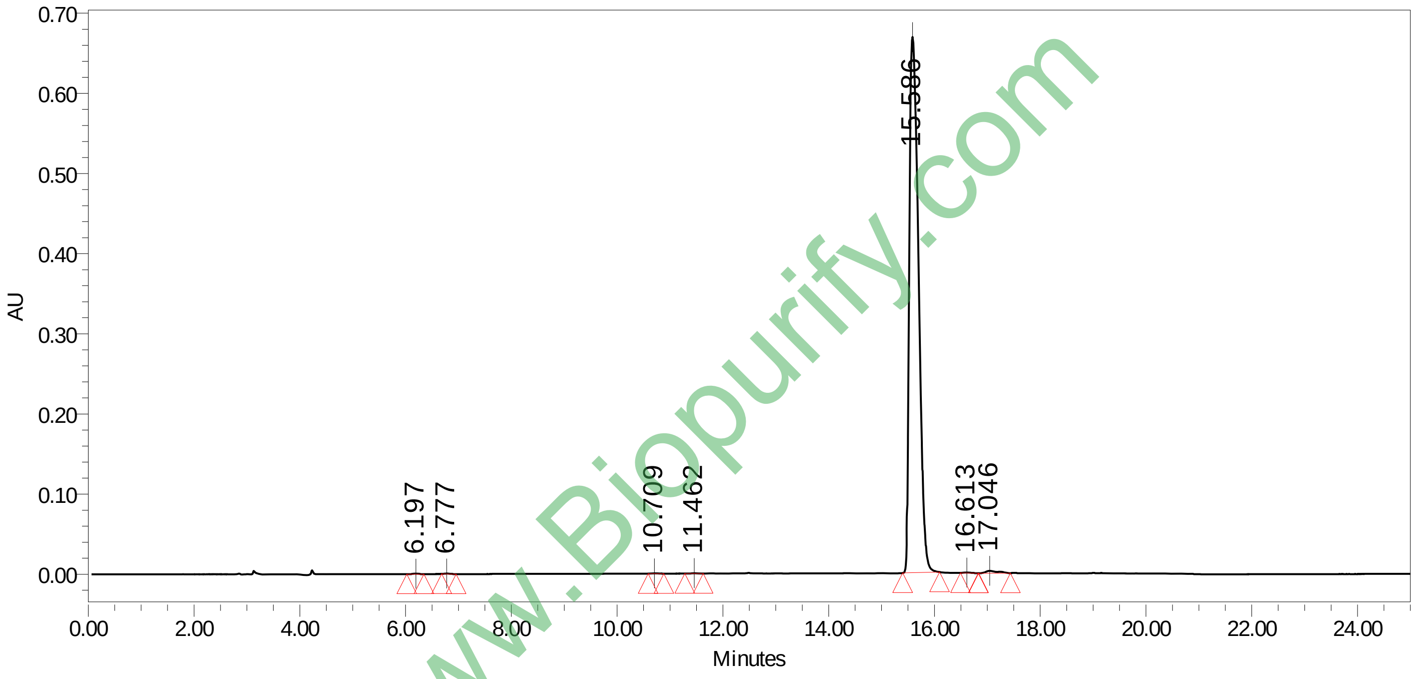

www.Biopurify.com

Peak Results

	Name	RT	Area	% Area	Height (µV)	USP Plate Count	USP Resolution
1		6.197	3903	0.05	562	22288.07	
2		6.777	3675	0.05	542	23204.47	3.15
3		10.709	3767	0.05	461	31341.08	19.81
4		11.462	3861	0.05	474	36582.54	3.47
5		15.586	8044771	99.19	668404	39016.90	15.48
6		16.613	5835	0.07	670	73627.51	3.68
7		17.046	44903	0.55	2857	32704.08	0.70
