# Supplementary material for: Identification of CB1 Ligands among Drugs, Phytochemicals and Natural-Like Compounds: Virtual Screening and In Vitro Verification
Source: ACS Chem Neurosci. 2022 Oct 5;13(20):2991–3007. doi: 10.1021/acschemneuro.2c00502 (PMC9585589; doi:10.1021/acschemneuro.2c00502)
Supplement: Supplementary file 3 — cn2c00502_si_003.zip [file cn2c00502_si_003.zip › Purity_identity_files/First iteration/Biopurify/BP1087-COA-PRF20061543.pdf]

成都普瑞法科技开发有限公司
Chengdu Biopurify Phytochemicals Ltd.

文件编码（DOC NO.）：**PRF-COA-BP108701**

产品分析证书
Certificate of Analysis

中文名称：连翘苷

English Name：Phillyrin
别名(Alias): Forsythin; Phillyroside; Chionanthin

产品编码(Cat. No.):BP1087
CAS Number: 487-41-2
分子式(M. F.): C27H34O11
分子量(M. W.): 534.558

批号(Batch No.): PRF20061543
报告日期(Report date): 2020-06-16

Structure

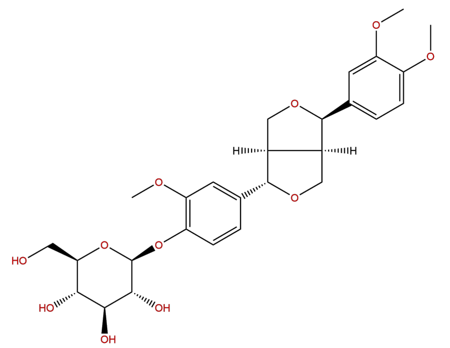

检验结果（Analytical result）：

检验项目（Test Item）	检验指标（Specifications）	检验结果（Results）
外观Appearance	Off-white powder	Off-white powder
干燥失重Loss on drying	＜3.0%	1.2%
纯度Purity (HPLC-DAD,277nm)*	≥98.0%	98.57%
质谱Mass	534.5±1	Conforms
核磁NMR	Comply with the structure	Conforms

*** 色谱图见附件（Please find HPLC chromatogram attached.）**

检测方法（Test Method）： Column: Welth Ultimate XB-C18 4.6*150mm,3.0um; **Column temperature**: 30℃; **Detection Mode:** UV277nm; **Flow Rate:** 1.0ml/min;

Sample dissolution: Methanol; **Mobile Phase**: A,Acetonitrile **B**, 0.1% Phosphoric acid in water; **Gradient elution:** A,20%-30%,15min.

贮存条件（Storage）:2~8℃, protected from light, keep package airproofed when not in use.

复测期（Retest date）:Three years (2023-06-15) under conditions list above.

QC: Zhang Ling　　　　　　　　QA: MengPan

Date: 2020-06-16　　　　　　　　Date: 2020-06-16

Chengdu Biopurify Phytochemicals Ltd. 成都普瑞法科技开发有限公司 质检专用章 Quality Approval Seal

备注(Remarks):The sample solutions should be prepared and used on the same day, it is the best preparing the solutions immediately before use. If the solutons have to be made up in advance, it should be made as aliquots in tightly sealed vials at less than -20℃. Generally, these might be useable for up to two weeks. The quality of the product is only guaranteed to comply with the attached COA.

In case of quality issue, please contact us within 30 days after receipt of the product.

Tel：+86-28-82633397　Fax：+86-28-82633165

http://www.phytopurify.com　Email：sales@biopurify.com　biopurify@gmail.com

SAMPLE INFORMATION

Sample Name:	Phillyrin	Acquired By:	PRF01
Sample Type:	未知	Sample Set Name	
Vial:	40	Acq. Method Set:	Phillyrin
Injection #:	1	Processing Methoc	sample
Injection Volume:	10.00 ul	Channel Name:	277.0 纳米
Run Time:	25.0 Minutes	Proc. Chnl. Descr.:	PDA 277.0 纳米 (190-600) 纳米
Date Acquired:	2020/6/16 9:46:21 CST		
Date Processed:	2020/6/16 10:27:36 CST		

Auto-Scaled Chromatogram

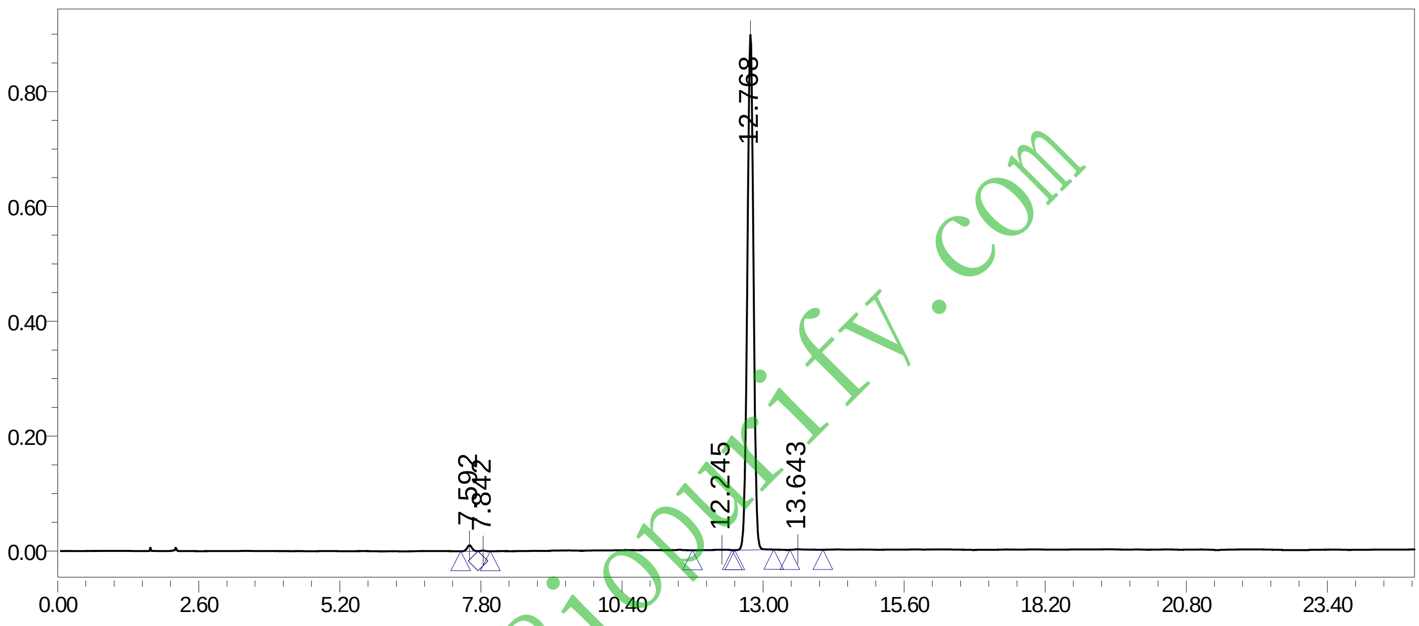

www.Biopurify.com

Peak Results

	RT	Area	% Area	Height	Plate Count	Resolution
1	7.592	66766	0.93	10419	31615.97	
2	7.842	7212	0.10	1051	41079.92	1.31
3	12.245	13725	0.19	597	517.79	9.32
4	12.768	7059395	98.57	896863	60303.46	1.11
5	13.643	14784	0.21	1266	60182.25	3.81
