# Supplementary material for: Identification of CB1 Ligands among Drugs, Phytochemicals and Natural-Like Compounds: Virtual Screening and In Vitro Verification
Source: ACS Chem Neurosci. 2022 Oct 5;13(20):2991–3007. doi: 10.1021/acschemneuro.2c00502 (PMC9585589; doi:10.1021/acschemneuro.2c00502)
Supplement: Supplementary file 3 — cn2c00502_si_003.zip [file cn2c00502_si_003.zip › Purity_identity_files/First iteration/Biopurify/BP1305-COA-PRF10110641.pdf]

成都普瑞法科技开发有限公司
Chengdu Biopurify Phytochemicals Ltd.

产品分析证书
Certificate of Analysis

中文名称：水飞蓟宾

English Name：Silybin(Mixture A&B)
别名(**Alias**): Silybin(Mixture A&B)

产品编码(**Cat. No.**):BP1305
CAS Number: 22888-70-6
分子式(**M. F.**): C25H22O10
分子量(**M. W.**): 482.441

批号(**Batch No.**): PRF10110641
报告日期(**Report date**): 2019-11-06

Structure

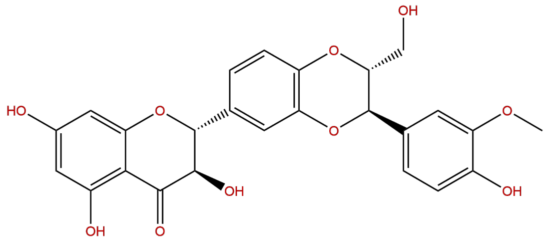

检验结果（**Analytical result**）：

检验项目（Test Item）	检验指标（Specifications）	检验结果（Results）
外观Appearance	Off-white powder	Off-white powder
纯度Purity (HPLC-DAD,287nm)*	≥98.0%	98.95%
质谱Mass	482.4±1	Conforms
核磁NMR	Comply with the structure	Conforms
水分Water Content	＜3.0%	1.46%

* 色谱图见附件（**Please find HPLC chromatogram attached.**）

检测方法（Test Method）： **Column:** Agilent 5HC-C18,4.6*250mm,5um; **Column temperature**: 30℃; **Detection Mode:** UV287nm; **Flow Rate:** 1.0ml/min;

Sample dissolution: Methanol; **Mobile Phase: A** Methanol **B,** Water；**Isocratic elution:** A,45%.

贮存条件（**Storage**）:2~8℃, protected from light, keep package airproofed when not in use.

复测期（**Retest date**）:Three years (2022-11-05) under conditions list above.

QC: Zhang Ling
Date: 2019-11-06

QA: MengPan
Date: 2019-11-06

备注(**Remarks**):The sample solutions should be prepared and used on the same day. It is the best preparing the solutions immediately before use. If the solutons have to be made up in advance, it should be made as aliquots in tightly sealed vials at less than -20℃. Generally, these might be useable for up to two weeks. The quality of the product is only guaranteed to comply with the attached COA.

In case of quality issue, please contact us within 30 days after receipt of the product.

质检专用章 Quality Approval Seal

Tel：+86-28-82633397 Fax：+86-28-82633165
http://www.phytopurify.com Email：sales@biopurify.com biopurify@gmail.com

SAMPLE INFORMATION

Sample Name:	Silybin PRF10110641	Acquired By:	prf01
Sample Type:	未知	Sample Set Name:	
Vial:	41	Acq. Method Set:	Silybin
Injection #:	1	Processing Method:	sample
Injection Volume:	10.00 ul	Channel Name:	287.0 纳米
Run Time:	25.0 Minutes	Proc. Chnl. Descr.:	PDA 287.0 纳米
Date Acquired:	2019-11-6 9:58:34 CST		
Date Processed:	2019-11-6 10:28:42 CST		

Auto-Scaled Chromatogram

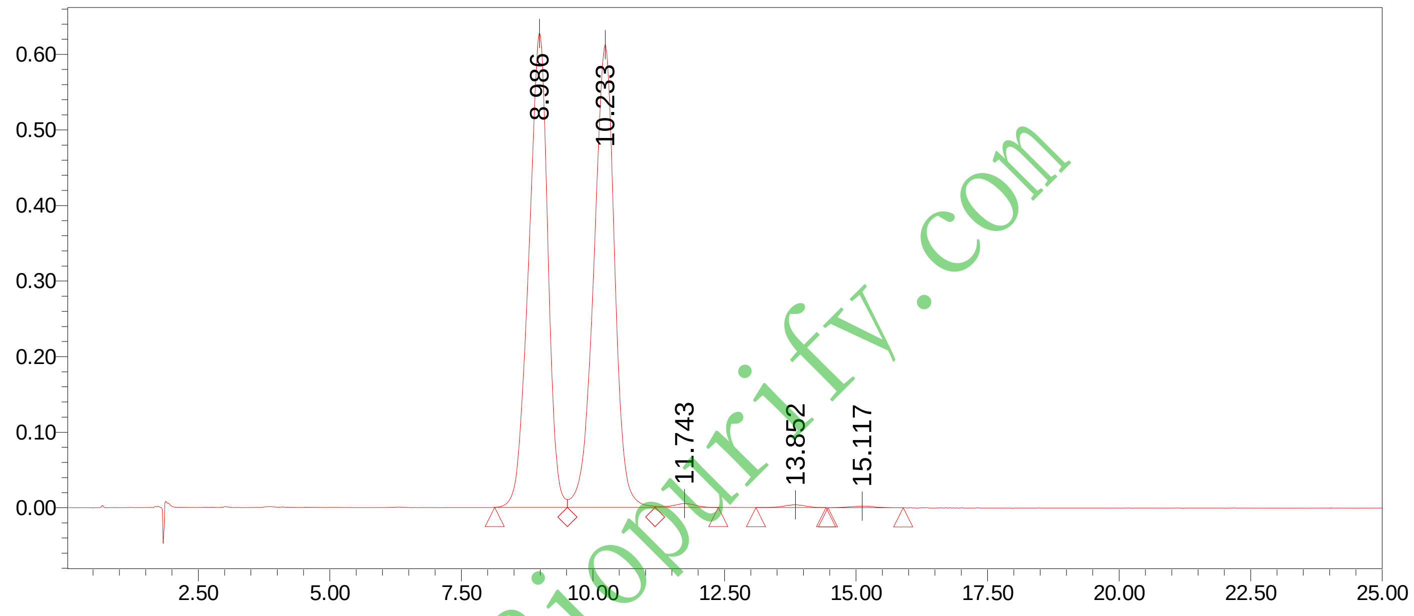

www.Biopurify.com

Peak Results

	RT	Area	% Area	高度 (微伏)	USP 分离度	USP 理论塔板数
1	8.986	15848930	47.98	627654		2901.30
2	10.233	16834507	50.97	612226	1.73	3237.51
3	11.743	156060	0.47	5061	1.89	3486.70
4	13.852	111729	0.34	3747	2.61	5912.78
5	15.117	77814	0.24	2413	1.47	4911.41
