# Supplementary material for: Identification of CB1 Ligands among Drugs, Phytochemicals and Natural-Like Compounds: Virtual Screening and In Vitro Verification
Source: ACS Chem Neurosci. 2022 Oct 5;13(20):2991–3007. doi: 10.1021/acschemneuro.2c00502 (PMC9585589; doi:10.1021/acschemneuro.2c00502)
Supplement: Supplementary file 3 — cn2c00502_si_003.zip [file cn2c00502_si_003.zip › Purity_identity_files/First iteration/Biopurify/BP1393-COA-PRF9101502.pdf]

成都普瑞法科技开发有限公司
Chengdu Biopurify Phytochemicals Ltd.

文件编码（DOC NO.）：PRF-COA-BP139301

产品分析证书
Certificate of Analysis

中文名称：银椴苷

English Name：Tiliroside
别名**(Alias):** 6″-O-trans-p-Coumaroylastragalin

产品编码**(Cat. No.):**BP1393
CAS Number: 20316-62-5
分子式**(M. F.):** C30H26O13
分子量**(M. W.)**: 594.525

批号**(Batch No.):** PRF9101502
报告日期**(Report date):** 2018-10-15

Structure

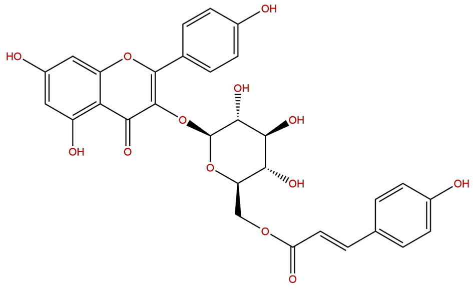

检验结果（**Analytical result**）：

检验项目（**Test Item**）	检验指标（**Specifications**）	检验结果（**Results**）
外观Appearance	Light yellow powder	Light yellow powder
纯度Purity (HPLC-DAD,315nm)*	≥98.0%	99.11%
质谱Mass	594.5±1	Conforms
核磁NMR	Comply with the structure	Conforms
水分Water Content	＜5.0%	3.78%

* 色谱图见附件（**Please find HPLC chromatography attached.**）

检测方法（Test Method）： **Column**: Thermo BDS Hypersil C18 4.6*100mm， 2.4μm; **Column temperature**: 30℃; **Detection Mode**:UV315nm； **Flow Rate**: 1.0ml/min; **Sample dissolution**: Methanol; **Mobile Phase**: **A**,Acetonitrile **B**, 0.1% Phosphoric acid in water;**Gradient elution**:A,20%-35%,15min.

贮存条件（Storage）:2~8℃, protected from light, keep package airproofed when not in use.

复测期（Retest date）:Three years (2021-10-14) under conditions list above.

QC: Zhang Ling

Date: 2018-10-15

QA: MengFan

Date: 2018-10-15

备注(Remarks):The sample solutions should be prepared and used on the same day. It is the best preparing the solutions immediately before use. If the solutons have to be made up in advance, it should be made as aliquots in tightly sealed vials at less than -20℃. Generally, these might be useable for up to two weeks. The quality of the product is only guaranteed to comply with the attached COA.

In case of quality issue, please contact us within 30 days after receipt of the product.

Chengdu Biopurify Phytochemicals 成都普瑞法科技开发有限公司 质检专用章 Quality Approval Seal

Tel：+86-28-82633397 Fax：+86-28-82633165
http://www.phytopurify.com Email：sales@biopurify.com biopurify@gmail.com
