# Supplementary material for: Identification of CB1 Ligands among Drugs, Phytochemicals and Natural-Like Compounds: Virtual Screening and In Vitro Verification
Source: ACS Chem Neurosci. 2022 Oct 5;13(20):2991–3007. doi: 10.1021/acschemneuro.2c00502 (PMC9585589; doi:10.1021/acschemneuro.2c00502)
Supplement: Supplementary file 3 — cn2c00502_si_003.zip [file cn2c00502_si_003.zip › Purity_identity_files/First iteration/Biopurify/BP1475-COA-PRF9101901.pdf]

成都普瑞法科技开发有限公司
Chengdu Biopurify Phytochemicals Ltd.

产品分析证书
Certificate of Analysis

中文名称：20(s)-原人参三醇

English Name：(20S)-Protopanaxatriol
别名(Alias):

产品编码(Cat. No.):BP1475
CAS Number: 34080-08-5
分子式(M. F.): C30H52O4
分子量(M. W.): 476.742

批号(Batch No.): PRF9101901
报告日期(Report date): 2018-10-19

Structure

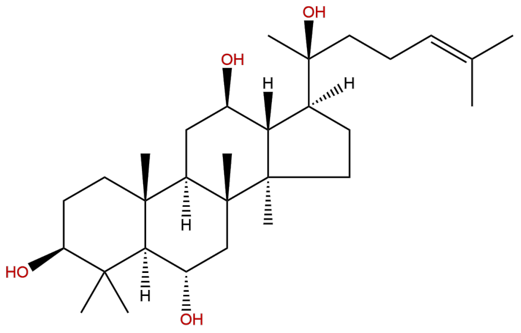

检验结果（Analytical result）：

检验项目（Test Item）	检验指标（Specifications）	检验结果（Results）
外观Appearance	Off-white powder	Off-white powder
干燥失重Loss on drying	＜3.0%	2.1%
纯度Purity (HPLC-ELSD)*	≥98.0%	99.94%
质谱Mass	476.7±1	Conforms
核磁NMR	Comply with the structure	Conforms

*** 色谱图见附件（Please find HPLC chromatography attached.）**

检测方法（Test Method）： **Column:** Agilent 5TC-C18,4.6*150mm,3um; **Column temperature**: 30℃; **Detection Mode:** ELSD; **Flow Rate:** 1.0ml/min;

Sample dissolution: Methanol; **Mobile Phase: A**, Acetonitrile **B,** Water;**Gradient elution:** A, 46%-56%,15min.

贮存条件（Storage）:2~8℃, protected from light, keep package airproofed when not in use.

复测期（Retest date）:Three years (2021-10-18) under conditions list above.

QC: Zhang Ling

Date: 2018-10-19

QA: MengPan

Date: 2018-10-19

备注(Remarks):The sample solutions should be prepared and used on the same day, it is the best preparing the solutions immediately before use. If the solutons have to be made up in advance, it should be made as aliquots in tightly sealed vials at less than -20℃. Generally, these might be useable for up to two weeks. The quality of the product is only guaranteed to comply with the attached COA.

In case of quality issue, please contact us within 30 days after receipt of the product.

Tel：+86-28-82633397 Fax：+86-28-82633165
http://www.phytopurify.com Email：sales@biopurify.com biopurify@gmail.com

SAMPLE INFORMATION

Sample Name:	20(S)-Protopanaxatriol	Acquired By:	System
Sample Type:	Unknown	Sample Set Name:	
Vial:	84	Acq. Method Set:	20
Injection #:	1	Processing Method	Samples
Injection Volume:	10.00 ul	Channel Name:	SATIN
Run Time:	25.0 Minutes	Proc. Chnl. Descr.:	
Date Acquired:	2018-10-19 15:07:49 CST		
Date Processed:	2018-10-19 15:39:51 CST		

Auto-Scaled Chromatogram

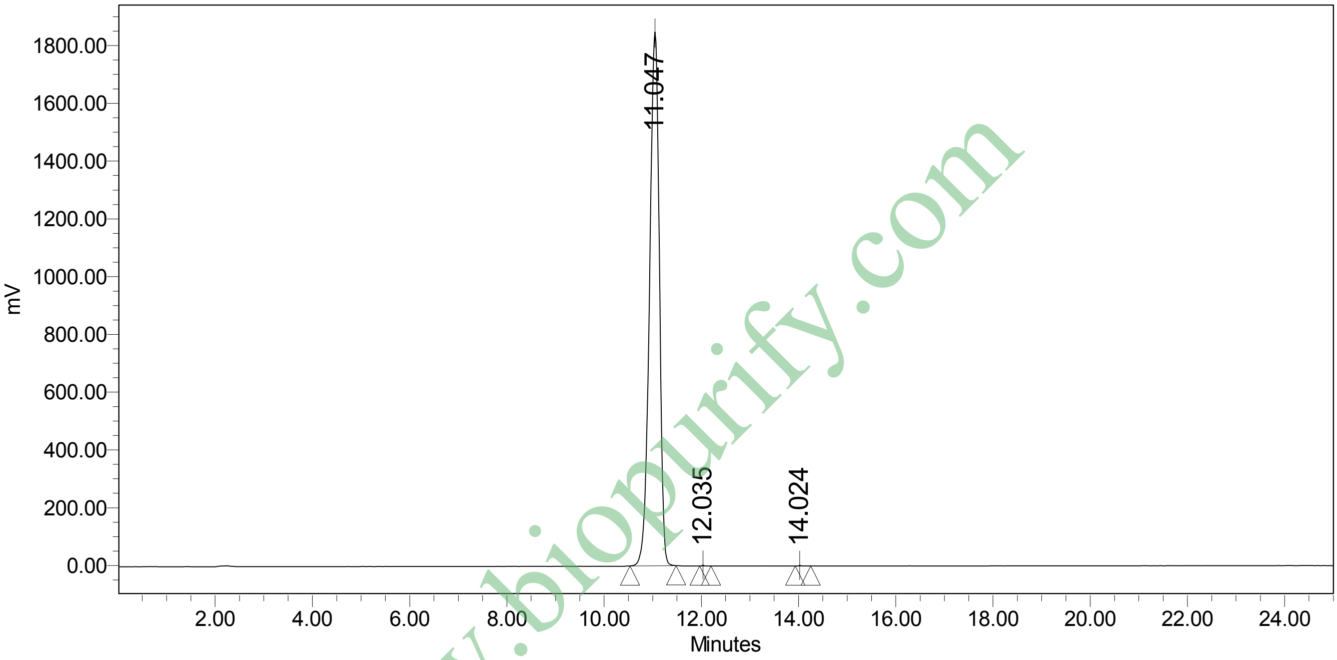

www.biopurify.com

Peak Results

	Name	RT	Area	% Area	USP Plate Count	USP Resolution
1		11.047	25738078	99.94	14495.02	
2		12.035	9310	0.04	550380.64	4.47
3		14.024	6889	0.03	851190.34	30.45
