# Supplementary material for: Identification of CB1 Ligands among Drugs, Phytochemicals and Natural-Like Compounds: Virtual Screening and In Vitro Verification
Source: ACS Chem Neurosci. 2022 Oct 5;13(20):2991–3007. doi: 10.1021/acschemneuro.2c00502 (PMC9585589; doi:10.1021/acschemneuro.2c00502)
Supplement: Supplementary file 3 — cn2c00502_si_003.zip [file cn2c00502_si_003.zip › Purity_identity_files/First iteration/Molport/AG001EU9_CoA.pdf]

CERTIFICATE OF ANALYSIS

Chemical Name: 2H-Benzimidazol-2-one,1,3-dihydro-1-[2-[4-[3-(trifluoromethyl)phenyl]-1-piperazinyl]ethyl]-,monohydrochloride

Chemical Structure:

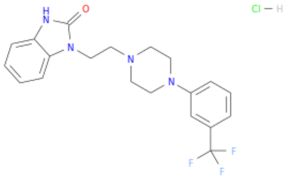

**Batch Number:**	AGN20-132583-1
**CAS Registry No.:**	147359-76-0
**Product ID:**	AG001EU9
**Manufacture Date:**	2020-09-21
**Storage Temperature:**	Room Temperature,Inert atmosphere
**Formula:**	C20H22ClF3N4O
**Molecular Weight:**	426.8631
**Quantity:**	250mg

Analysis Data:

**Test:**	**Specification:**	**Result:**
Appearance	White powder	Conforms
HNMR	Consistent with the structure	Conforms
Purity	95+%	Conforms

Conclusion: The above product meets the specifications of Angene.

Chase

Jessie

QC: Chase   Date: 2020-09-21   QA: Jessie   Date:2020-09-21

Angene International Limited
