# Supplementary material for: Identification of CB1 Ligands among Drugs, Phytochemicals and Natural-Like Compounds: Virtual Screening and In Vitro Verification
Source: ACS Chem Neurosci. 2022 Oct 5;13(20):2991–3007. doi: 10.1021/acschemneuro.2c00502 (PMC9585589; doi:10.1021/acschemneuro.2c00502)
Supplement: Supplementary file 3 — cn2c00502_si_003.zip [file cn2c00502_si_003.zip › Purity_identity_files/First iteration/Molport/AG001PAX_CoA.pdf]

CERTIFICATE OF ANALYSIS

Chemical Name: Benzenesulfonamide,4-(1,1-dimethylethyl)-N-[6-(2-hydroxyethoxy)-5-(2-methoxyphenoxy)[2,2'-bipyrimidin]-4-yl]-, monohydrate

Chemical Structure:

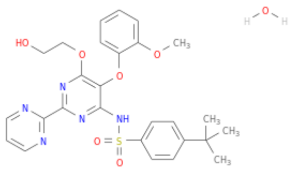

**Batch Number:**	AGN20-132589-2
**CAS Registry No.:**	157212-55-0
**Product ID:**	AG001PAX
**Manufacture Date:**	2020-09-21
**Storage Temperature:**	2-8℃
**Formula:**	C27H31N5O7S
**Molecular Weight:**	569.6293
**Quantity:**	100mg

Analysis Data:

Test:	Specification:	Result:
Appearance	White powder	Conforms
HNMR	Consistent with the structure	Conforms
Purity	98+%	Conforms

Conclusion: The above product meets the specifications of Angene.

Chase

Jessie

QC: Chase Date: 2020-09-21

QA: Jessie Date:2020-09-21

Angene International Limited
