# Supplementary material for: Identification of CB1 Ligands among Drugs, Phytochemicals and Natural-Like Compounds: Virtual Screening and In Vitro Verification
Source: ACS Chem Neurosci. 2022 Oct 5;13(20):2991–3007. doi: 10.1021/acschemneuro.2c00502 (PMC9585589; doi:10.1021/acschemneuro.2c00502)
Supplement: Supplementary file 3 — cn2c00502_si_003.zip [file cn2c00502_si_003.zip › Purity_identity_files/First iteration/Molport/AG0035KL_CoA.pdf]

CERTIFICATE OF ANALYSIS

Chemical Name: 5-Chloro-N-[[(S)-3-(4-(3-oxomorpholin-4-yl)phenyl)-2-oxo-1,3-oxazolidin-5-yl]methyl]-thiophene-2-carboxamide

Chemical Structure:

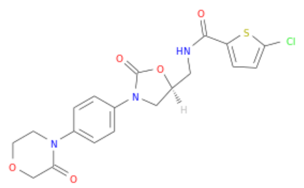

**Batch Number:**	2013-565
**CAS Registry No.:**	366789-02-8
**Product ID:**	AG0035KL
**Manufacture Date:**	2019-09-28
**Storage Temperature:**	2-8℃
**Formula:**	C19H18ClN3O5S
**Molecular Weight:**	435.8813
**Quantity:**	50mg

Analysis Data:

**Test:**	**Specification:**	**Result:**
Appearance		Conforms
HNMR	Consistent with the structure	Conforms
Purity	99%	Conforms

Conclusion: The above product meets the specifications of Angene.

Chase Jessie

QC: Chase Date: 2019-09-28 QA: Jessie Date:2019-09-28

Angene International Limited
