# Supplementary material for: Identification of CB1 Ligands among Drugs, Phytochemicals and Natural-Like Compounds: Virtual Screening and In Vitro Verification
Source: ACS Chem Neurosci. 2022 Oct 5;13(20):2991–3007. doi: 10.1021/acschemneuro.2c00502 (PMC9585589; doi:10.1021/acschemneuro.2c00502)
Supplement: Supplementary file 3 — cn2c00502_si_003.zip [file cn2c00502_si_003.zip › Purity_identity_files/First iteration/Molport/AG00DDUB__CoA.pdf]

CERTIFICATE OF ANALYSIS

Chemical Name: 5-O-[2-[benzyl(methyl)amino]ethyl] 3-O-methyl (4S)-2,6-dimethyl-4-(3-nitrophenyl)-1,4-dihydropyridine-3,5-dicarboxylate;hydrochloride

Chemical Structure:

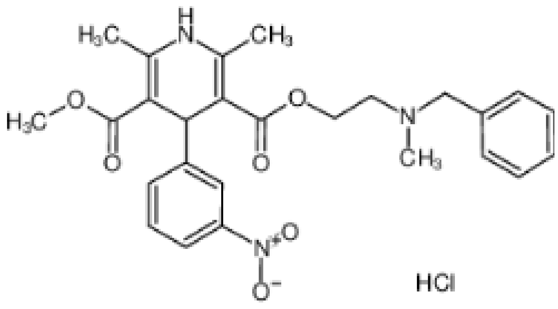

**Batch Number:**	AGN20-132579-2
**CAS Registry No.:**	54527-84-3
**Product ID:**	AG00DDUB
**Manufacture Date:**	2020-09-20
**Storage Temperature:**	2-8 ℃ ,Light sensitive,Inert atmosphere
**Formula:**	C26H30ClN3O6
**Molecular Weight:**	515.9859
**Quantity:**	100mg

Analysis Data:

Test:	Specification:	Result:
Appearance	solid	Conforms
HNMR	Consistent with the structure	Conforms
Purity	98%	Conforms

Conclusion: The above product meets the specifications of Angene.

Chase   Jessie

QC: Chase   Date: 2020-09-20   QA: Jessie   Date:2020-09-20

Angene International Limited
