# Supplementary material for: Identification of CB1 Ligands among Drugs, Phytochemicals and Natural-Like Compounds: Virtual Screening and In Vitro Verification
Source: ACS Chem Neurosci. 2022 Oct 5;13(20):2991–3007. doi: 10.1021/acschemneuro.2c00502 (PMC9585589; doi:10.1021/acschemneuro.2c00502)
Supplement: Supplementary file 3 — cn2c00502_si_003.zip [file cn2c00502_si_003.zip › Purity_identity_files/First iteration/Molport/AG00F9YQ_CoA.pdf]

CERTIFICATE OF ANALYSIS

Chemical Name: Urea,N-[(4-fluorophenyl)methyl]-N-(1-methyl-4-piperidinyl)-N'-[[4-(2-methylpropoxy)phenyl]methyl]-

Chemical Structure:

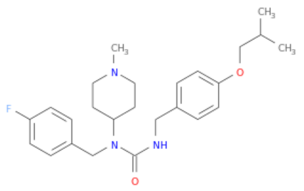

**Batch Number:**	AGN20-132571-2
**CAS Registry No.:**	706779-91-1
**Product ID:**	AG00F9YQ
**Manufacture Date:**	2020-09-20
**Storage Temperature:**	-20 ℃
**Formula:**	C25H34FN3O2
**Molecular Weight:**	427.5548
**Quantity:**	10mg

Analysis Data:

**Test:**	**Specification:**	**Result:**
Appearance	White powder	Conforms
HNMR	Consistent with the structure	Conforms
Purity	98%	Conforms

Conclusion: The above product meets the specifications of Angene.

Chase　　　　Jessie

QC: Chase　　Date: 2020-09-20　　QA: Jessie　　Date:2020-09-20

Angene International Limited
