# Supplementary material for: Identification of CB1 Ligands among Drugs, Phytochemicals and Natural-Like Compounds: Virtual Screening and In Vitro Verification
Source: ACS Chem Neurosci. 2022 Oct 5;13(20):2991–3007. doi: 10.1021/acschemneuro.2c00502 (PMC9585589; doi:10.1021/acschemneuro.2c00502)
Supplement: Supplementary file 3 — cn2c00502_si_003.zip [file cn2c00502_si_003.zip › Purity_identity_files/First iteration/Molport/AG00I57D_CoA.pdf]

Angene

CERTIFICATE OF ANALYSIS

Chemical Name: 1,2-Cyclopentanediol,3-[7-[[(1R,2S)-2-(3,4-difluorophenyl)cyclopropyl]amino]-5-(propylthio)-3H-1,2,3-triazolo[4,5-d]pyrimidin-3-yl]-5-(2-hydroxyethoxy)-,(1S,2S,3R,5S)-

Chemical Structure:

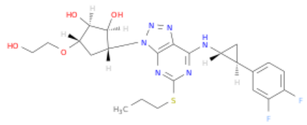

**Batch Number:**	AK20-99754-9-1
**CAS Registry No.:**	274693-27-5
**Product ID:**	AG00I57D
**Manufacture Date:**	2020-08-05
**Storage Temperature:**	-20 ℃ ,Light sensitive,Inert atmosphere
**Formula:**	C23H28F2N6O4S
**Molecular Weight:**	522.5680
**Quantity:**	100mg

Analysis Data:

**Test:**	**Specification:**	**Result:**
Appearance	White powder	Conforms
HNMR	Consistent with the structure	Conforms
Purity	98+%	Conforms

Conclusion: The above product meets the specifications of Angene.

Chase   Jessie

QC: Chase   Date: 2020-08-05   QA: Jessie   Date:2020-08-05
