# Supplementary material for: Identification of CB1 Ligands among Drugs, Phytochemicals and Natural-Like Compounds: Virtual Screening and In Vitro Verification
Source: ACS Chem Neurosci. 2022 Oct 5;13(20):2991–3007. doi: 10.1021/acschemneuro.2c00502 (PMC9585589; doi:10.1021/acschemneuro.2c00502)

AK Scientific HPLC

AK Scientific, Inc.
30023 Ahern Ave
Union City, CA 94587
USA
sales@aksci.com

<Sample Information>

Sample ID	: JL29738		
Method Filename	: AKHPLC_column2_ALL.lcm		
Batch Filename	: 08-07-2019_122020_934 AM_1292020_1432 PM_612020_845 AM_642021_1634 PM_		
Vial #	: 1-99		
Injection Volume	: 4 uL		
Date Acquired	: 7/12/2022 1:50:03 PM	Acquired by	: operator
Date Processed	: 7/12/2022 2:00:07 PM	Processed by	: operator

<Chromatogram>

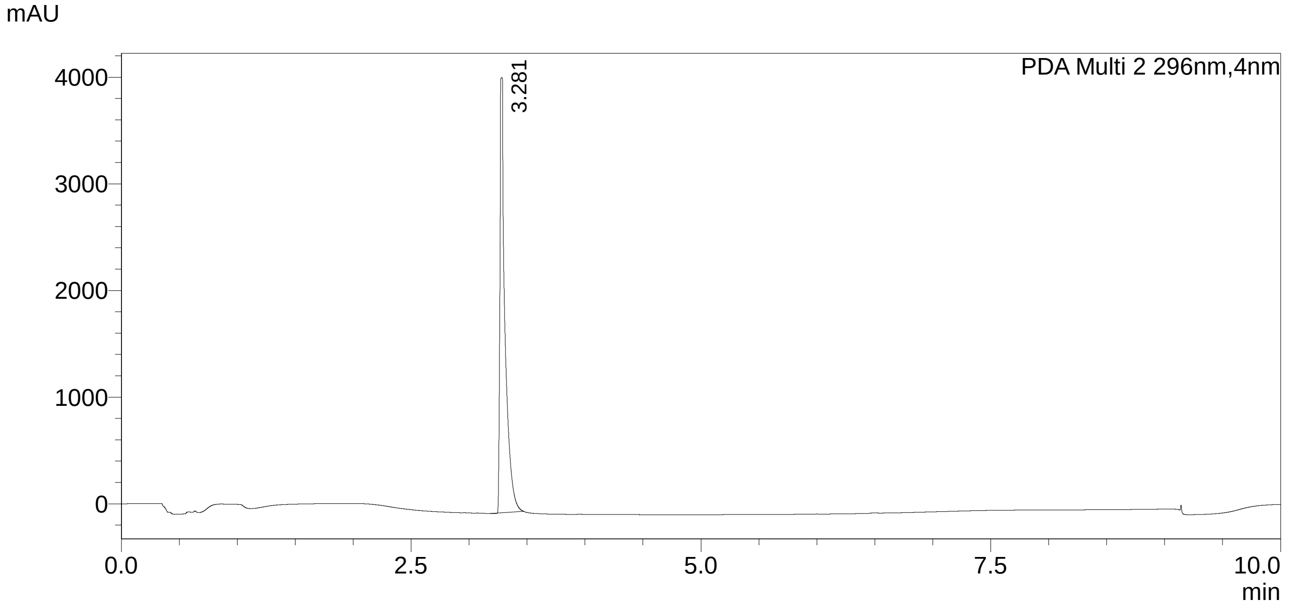

Peak Table

PDA Ch2 296nm

Peak#	Ret. Time	Area	Height	Area%
1	3.281	12600443	4078598	100.000
Total		12600443	4078598	100.000

AK Scientific LCMS

AK Scientific, Inc.
30023 Ahern Ave
Union City, CA 94587
USA
sales@aksci.com

<Sample Information>

Sample ID	: JL29738		
Method Filename	: AKHPLC_column2_ALL.lcm		
Batch Filename	: 08-07-2019_122020_934 AM_1292020_1432 PM_612020_845 AM_642021_1634 PM_		
Vial #	: 1-99		
Injection Volume	: 4 uL		
Date Acquired	: 7/12/2022 1:50:03 PM	Acquired by	: operator
Date Processed	: 7/12/2022 2:00:07 PM	Processed by	: operator

MS Chromatogram

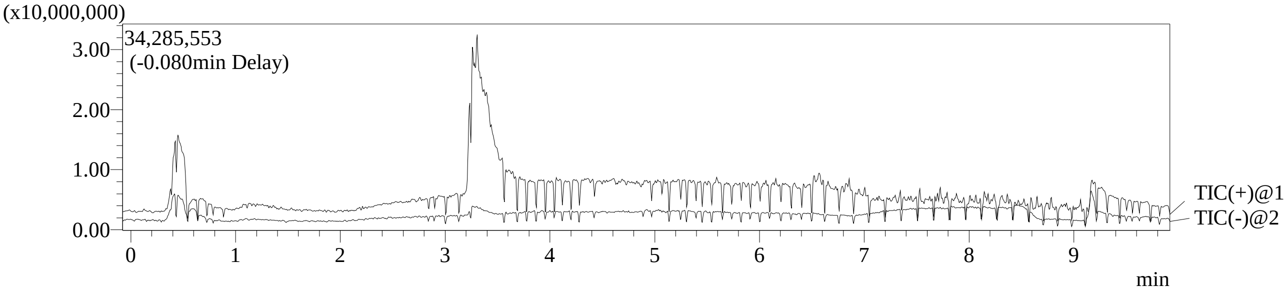

MS Spectrum

Peak #: 1 Ret. Time: 3.170-3.735
Base Peak: 427 Polarity: Positive

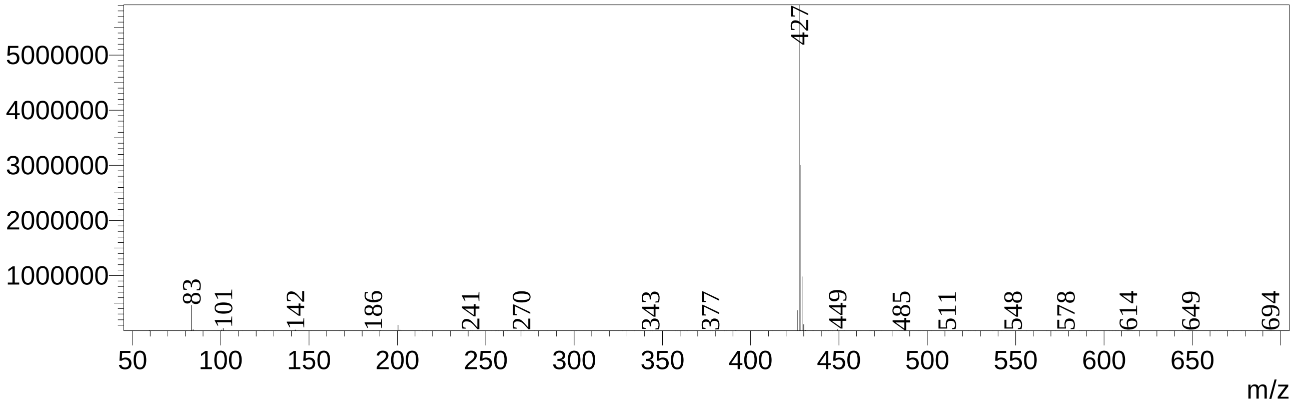

Supplement: Supplementary file 3 — cn2c00502_si_003.zip [file cn2c00502_si_003.zip › Purity_identity_files/First iteration/Molport/B599_JL29738_LCMS_HPLC (296nm).pdf]
