# Supplementary material for: Identification of CB1 Ligands among Drugs, Phytochemicals and Natural-Like Compounds: Virtual Screening and In Vitro Verification
Source: ACS Chem Neurosci. 2022 Oct 5;13(20):2991–3007. doi: 10.1021/acschemneuro.2c00502 (PMC9585589; doi:10.1021/acschemneuro.2c00502)

Travoprost

**Cat. No.:**	HY-B0584		
**CAS No.:**	157283-68-6		
**Molecular Formula:**	$C_{26}H_{35}F_3O_6$		
**Molecular Weight:**	500.55		
**Target:**	Prostaglandin Receptor		
**Pathway:**	GPCR/G Protein		
**Storage:**	Pure form	-20°C	3 years
	In solvent	-80°C	6 months
		-20°C	1 month

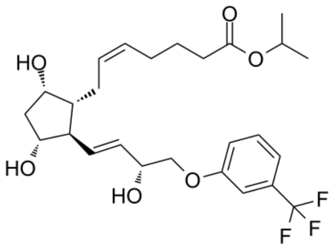

SOLVENT & SOLUBILITY

In Vitro

DMSO : ≥ 41.67 mg/mL (83.25 mM)

* "≥" means soluble, but saturation unknown.

Preparing Stock Solutions	Concentration / Solvent / Mass	1 mg	5 mg	10 mg
	1 mM	1.9978 mL	9.9890 mL	19.9780 mL
	5 mM	0.3996 mL	1.9978 mL	3.9956 mL
	10 mM	0.1998 mL	0.9989 mL	1.9978 mL

Please refer to the solubility information to select the appropriate solvent.

In Vivo

1. Add each solvent one by one: **10% DMSO >> 40% PEG300 >> 5% Tween-80 >> 45% saline**
   Solubility: ≥ 2.08 mg/mL (4.16 mM); Clear solution
2. Add each solvent one by one: **10% DMSO >> 90% (20% SBE-β-CD in saline)**
   Solubility: ≥ 2.08 mg/mL (4.16 mM); Clear solution
3. Add each solvent one by one: **10% DMSO >> 90% corn oil**
   Solubility: ≥ 2.08 mg/mL (4.16 mM); Clear solution

BIOLOGICAL ACTIVITY

Description

Travoprost is used to treat glaucoma and ocular hypertension.Target: OthersTravoprost ophthalmic solution is a topical medication used for controlling the progression of glaucoma or ocular hypertension, by reducing intraocular pressure. It is a synthetic prostaglandin analog (or more specifically, an analog of prostaglandin F2α) that works by increasing the outflow of aqueous fluid from the eyes. From Wikipedia.

CUSTOMER VALIDATION

- **Front Pharmacol**. 2019 May 24;10:549.

See more customer validations on www.MedChemExpress.com

REFERENCES

[1]. http://en.wikipedia.org/wiki/Travoprost

Caution: Product has not been fully validated for medical applications. For research use only.

Tel: 609-228-6898 Fax: 609-228-5909 E-mail: tech@MedChemExpress.com
Address: 1 Deer Park Dr, Suite Q, Monmouth Junction, NJ 08852, USA

Certificate of Analysis

**Product Name:**	Travoprost
**Cat. No.:**	HY-B0584
**CAS No.:**	157283-68-6
**Batch No.:**	42692
**Chemical Name:**	5-Heptenoic acid, 7-[(1R,2R,3R,5S)-3,5-dihydroxy-2-[(1E,3R)-3-hydroxy-4-[3-(trifluoromethyl)phenoxy]-1-buten-1-yl]cyclopentyl]-, 1-methylethyl ester, (5Z)-

PHYSICAL AND CHEMICAL PROPERTIES

**Molecular Formula:**	$C_{26}H_{35}F_3O_6$		
**Molecular Weight:**	500.55		
**Storage:**	Pure form	-20°C	3 years
	In solvent	-80°C	6 months
		-20°C	1 month

Chemical Structure:

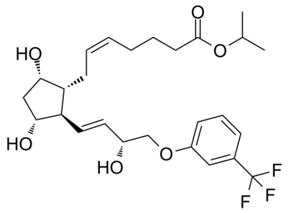

ANALYTICAL DATA

**Appearance:**	Colorless to light yellow (Oil)
**^{1}H NMR Spectrum:**	Consistent with structure
**LCMS:**	Consistent with structure
**Purity (LCMS):**	99.84%
**Conclusion:**	The product has been tested and complies with the given specifications.

Caution: Product has not been fully validated for medical applications. For research use only.

Tel: 609-228-6898 Fax: 609-228-5909 E-mail: tech@MedChemExpress.com
Address: 1 Deer Park Dr, Suite Q, Monmouth Junction, NJ 08852, USA

Safety Data Sheet

Revision Date: Feb-27-2020
Print Date: Feb-27-2020

1. PRODUCT AND COMPANY IDENTIFICATION

1.1 Product identifier

Product name :	Travoprost
Catalog No. :	HY-B0584
CAS No. :	157283-68-6

1.2 Relevant identified uses of the substance or mixture and uses advised against

Identified uses :	Laboratory chemicals, manufacture of substances.

1.3 Details of the supplier of the safety data sheet

Company:	MedChemExpress USA
Tel:	609-228-6898
Fax:	609-228-5909
E-mail:	sales@medchemexpress.com

1.4 Emergency telephone number

Emergency Phone #:	609-228-6898

2. HAZARDS IDENTIFICATION

2.1 Classification of the substance or mixture

GHS Classification in accordance with 29 CFR 1910 (OSHA HCS)

Acute toxicity, Oral (Category 4) , H302

Acute aquatic toxicity (Category 1) , H400

Chronic aquatic toxicity (Category 1) , H410

2.2 GHS Label elements, including precautionary statements

Pictogram

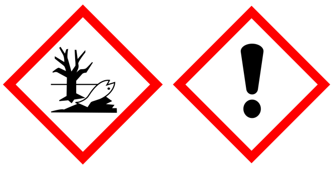

Signal word Danger

Hazard statement(s)

H302 Harmful if swallowed.

H410 Very toxic to aquatic life with long lasting effects.

Precautionary statement(s)

P264 Wash skin thoroughly after handling.

P270 Do not eat, drink or smoke when using this product.

P273 Avoid release to the environment.

P301 + P312 IF SWALLOWED: Call a POISON CENTER or doctor/ physician if you feel unwell.

P330 Rinse mouth.

P391 Collect spillage.

P501 Dispose of contents/ container to an approved waste disposal plant.

2.3 Other hazards

None.

3. COMPOSITION/INFORMATION ON INGREDIENTS

3.1 Substances

Synonyms:	Fluprostenol isopropyl ester;AL6221;Flu-Ipr
Formula:	C26H35F3O6
Molecular Weight:	500.55
CAS No. :	157283-68-6

4. FIRST AID MEASURES

4.1 Description of first aid measures

Eye contact

Remove any contact lenses, locate eye-wash station, and flush eyes immediately with large amounts of water. Separate eyelids with fingers to ensure adequate flushing. Promptly call a physician.

Skin contact

Rinse skin thoroughly with large amounts of water. Remove contaminated clothing and shoes and call a physician.

Inhalation

Immediately relocate self or casualty to fresh air. If breathing is difficult, give cardiopulmonary resuscitation (CPR). Avoid mouth-to-mouth resuscitation.

Ingestion

Wash out mouth with water; Do NOT induce vomiting; call a physician.

4.2 Most important symptoms and effects, both acute and delayed

The most important known symptoms and effects are described in the labelling (see section 2.2).

4.3 Indication of any immediate medical attention and special treatment needed

Treat symptomatically.

5. FIRE FIGHTING MEASURES

5.1 Extinguishing media

Suitable extinguishing media

Use water spray, dry chemical, foam, and carbon dioxide fire extinguisher.

5.2 Special hazards arising from the substance or mixture

During combustion, may emit irritant fumes.

5.3 Advice for firefighters

Wear self-contained breathing apparatus and protective clothing.

6. ACCIDENTAL RELEASE MEASURES

6.1 Personal precautions, protective equipment and emergency procedures

Use full personal protective equipment. Avoid breathing vapors, mist, dust or gas. Ensure adequate ventilation. Evacuate personnel to safe areas.

Refer to protective measures listed in sections 8.

6.2 Environmental precautions

Try to prevent further leakage or spillage. Keep the product away from drains or water courses.

6.3 Methods and materials for containment and cleaning up

Absorb solutions with finely-powdered liquid-binding material (diatomite, universal binders); Decontaminate surfaces and equipment by scrubbing with alcohol; Dispose of contaminated material according to Section 13.

7. HANDLING AND STORAGE

7.1 Precautions for safe handling

Avoid inhalation, contact with eyes and skin. Avoid dust and aerosol formation. Use only in areas with appropriate exhaust ventilation.

7.2 Conditions for safe storage, including any incompatibilities

Keep container tightly sealed in cool, well-ventilated area. Keep away from direct sunlight and sources of ignition.

Recommended storage temperature:	Pure form	-20°C	3 years
	In solvent	-80°C	6 months
		-20°C	1 month

Shipping at room temperature if less than 2 weeks.

7.3 Specific end use(s)

No data available.

8. EXPOSURE CONTROLS/PERSONAL PROTECTION

8.1 Control parameters

Components with workplace control parameters

This product contains no substances with occupational exposure limit values.

8.2 Exposure controls

Engineering controls

Ensure adequate ventilation. Provide accessible safety shower and eye wash station.

Personal protective equipment

**Eye protection**	Safety goggles with side-shields.
**Hand protection**	Protective gloves.
**Skin and body protection**	Impervious clothing.
**Respiratory protection**	Suitable respirator.
**Environmental exposure controls**	Keep the product away from drains, water courses or the soil. Clean spillages in a safe way as soon as possible.

9. PHYSICAL AND CHEMICAL PROPERTIES

9.1 Information on basic physical and chemical properties

**Appearance**	Oil
**Odor**	No data available
**Odor threshold**	No data available
**pH**	No data available
**Melting/freezing point**	No data available
**Boiling point/range**	No data available
**Flash point**	No data available
**Evaporation rate**	No data available
**Flammability (solid, gas)**	No data available
**Upper/lower flammability or explosive limits**	No data available
**Vapor pressure**	No data available

**Vapor density**	No data available
**Relative density**	No data available
**Water Solubility**	No data available
**Partition coefficient**	No data available
**Auto-ignition temperature**	No data available
**Decomposition temperature**	No data available
**Viscosity**	No data available
**Explosive properties**	No data available
**Oxidizing properties**	No data available

9.2 Other safety information

No data available.

10. STABILITY AND REACTIVITY

10.1 Reactivity

No data available.

10.2 Chemical stability

Stable under recommended storage conditions.

10.3 Possibility of hazardous reactions

No data available.

10.4 Conditions to avoid

No data available.

10.5 Incompatible materials

Strong acids/alkalis, strong oxidising/reducing agents.

10.6 Hazardous decomposition products

Under fire conditions, may decompose and emit toxic fumes.

Other decomposition products - no data available.

11.TOXICOLOGICAL INFORMATION

11.1 Information on toxicological effects

Acute toxicity

Classified based on available data. For more details, see section 2

Skin corrosion/irritation

Classified based on available data. For more details, see section 2

Serious eye damage/irritation

Classified based on available data. For more details, see section 2

Respiratory or skin sensitization

Classified based on available data. For more details, see section 2

Germ cell mutagenicity

Classified based on available data. For more details, see section 2

Carcinogenicity

IARC: No component of this product present at a level equal to or greater than 0.1% is identified as probable, possible or confirmed human carcinogen by IARC.

ACGIH: No component of this product present at a level equal to or greater than 0.1% is identified as a potential or confirmed carcinogen by ACGIH.

NTP: No component of this product present at a level equal to or greater than 0.1% is identified as a anticipated or confirmed carcinogen by NTP.

OSHA: No component of this product present at a level equal to or greater than 0.1% is identified as a potential or confirmed carcinogen by OSHA.

Reproductive toxicity

Classified based on available data. For more details, see section 2

Specific target organ toxicity - single exposure

Classified based on available data. For more details, see section 2

Specific target organ toxicity - repeated exposure

Classified based on available data. For more details, see section 2

Aspiration hazard

Classified based on available data. For more details, see section 2

Additional information

This information is based on our current knowledge. However the chemical, physical, and toxicological properties have not been completely investigated.

12. ECOLOGICAL INFORMATION

12.1 Toxicity

No data available.

12.2 Persistence and degradability

No data available.

12.3 Bioaccumlative potential

No data available.

12.4 Mobility in soil

No data available.

12.5 Results of PBT and vPvB assessment

PBT/vPvB assessment unavailable as chemical safety assessment not required or not conducted.

12.6 Other adverse effects

No data available.

13. DISPOSAL CONSIDERATIONS

13.1 Waste treatment methods

Product

Dispose substance in accordance with prevailing country, federal, state and local regulations.

Contaminated packaging

Conduct recycling or disposal in accordance with prevailing country, federal, state and local regulations.

14. TRANSPORT INFORMATION

DOT (US)

This substance is considered to be non-hazardous for transport.

IMDG

UN number: 3077

Class: 9

Packing group: III

EMS-No: F-A, S-F

Proper shipping name: ENVIRONMENTALLY HAZARDOUS SUBSTANCE, SOLID, N.O.S.

Marine pollutant: Marine pollutant

IATA

UN number: 3077

Class: 9

Packing group: III

Proper shipping name: Environmentally hazardous substance, solid, n.o.s.

15. REGULATORY INFORMATION

SARA 302 Components:

No chemicals in this material are subject to the reporting requirements of SARA Title III, Section 302.

SARA 313 Components:

This material does not contain any chemical components with known CAS numbers that exceed the threshold (De Minimis) reporting levels established by SARA Title III, Section 313.

SARA 311/312 Hazards:

No SARA Hazards.

Massachusetts Right To Know Components:

No components are subject to the Massachusetts Right to Know Act.

Pennsylvania Right To Know Components:

No components are subject to the Pennsylvania Right to Know Act.

New Jersey Right To Know Components:

No components are subject to the New Jersey Right to Know Act.

California Prop. 65 Components:

This product does not contain any chemicals known to State of California to cause cancer, birth defects, or anyother reproductive harm.

16. OTHER INFORMATION

Copyright 2020 MedChemExpress. The above information is correct to the best of our present knowledge but does not purport to be all inclusive and should be used only as a guide. The product is for research use only and for experienced personnel. It must only be handled by suitably qualified experienced scientists in appropriately equipped and authorized facilities. The burden of safe use of this material rests entirely with the user. MedChemExpress disclaims all liability for any damage resulting from handling or from contact with this product.

Tel: 609-228-6898 Fax: 609-228-5909 E-mail: tech@MedChemExpress.com

Address: 1 Deer Park Dr, Suite Q, Monmouth Junction, NJ 08852, USA

Sample ID: BIZ2019-625-WYQ7, Catalog No: HY-B0584 Batch#42692, CDCL3

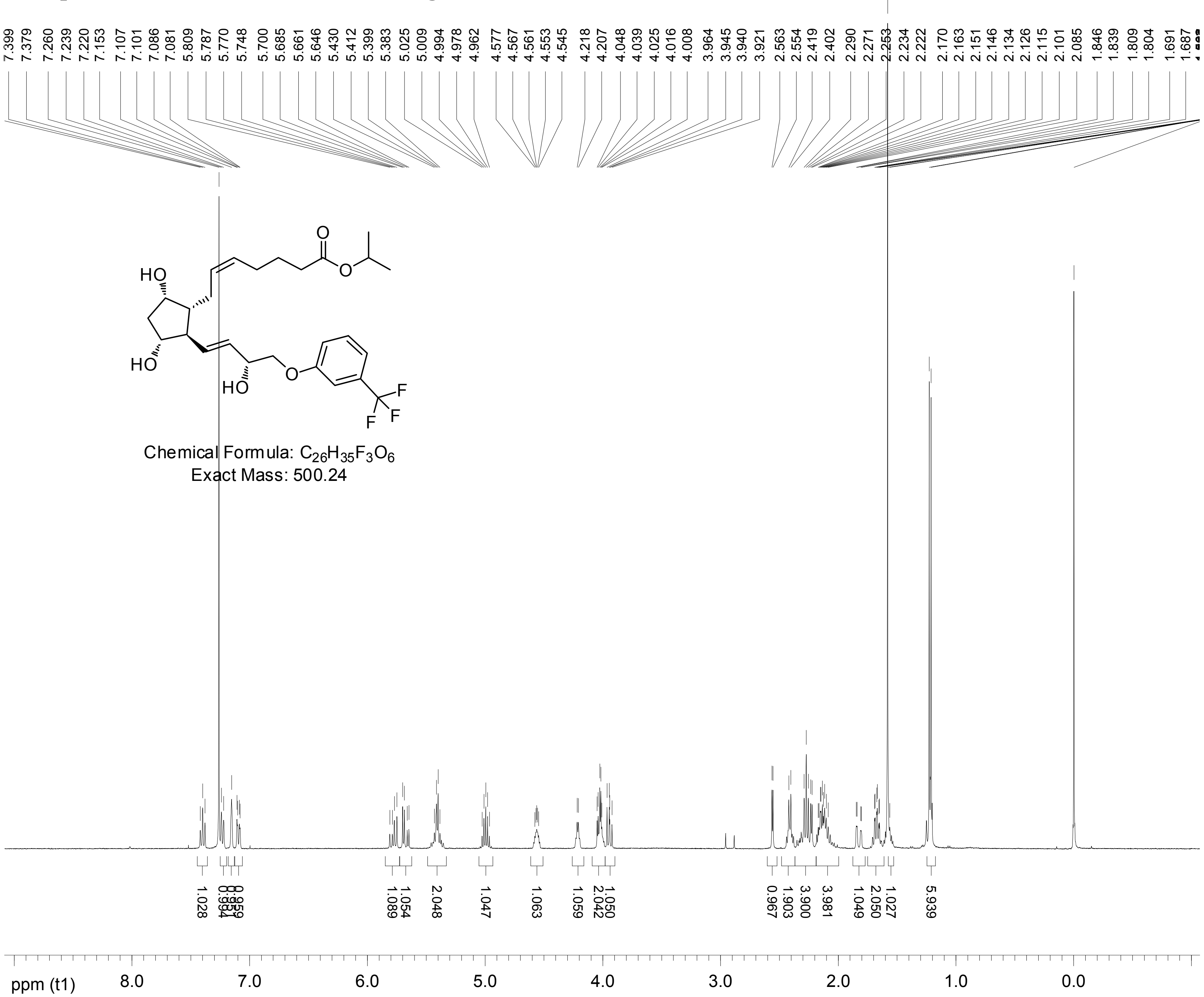

Date:
26 Jun 2019
Document's Title:
Catalog No: HY-B0584 Batch#42692

Spectrum Title:
BIZ2019-625-WYQ7-CDCL3-190625

Frequency (MHz):
(f1) 400.130
Original Points Count:
(f1) 32768
Actual Points Count:
(f1) 32768
Acquisition Time (sec):
(f1) 3.7224
Spectral Width (ppm):
(f1) 22.000
Pulse Program:
Unknown

File ..\DATA\2019\20190626\615\BIZ2019-625-WYQ7.D Tgt Mass (CHM):
Injection Date : 26 Jun 19 12:26 pm +0800 Seq. Line : 36
Sample Name : BIZ2019-625-WYQ7 Location : P1-A-07
Acq. Operator : ZJ_1432 Inj : 1
Spec. Reported : MS Integration Inj Volume : 2 ul
Acq. Method : D:\Chem32\1\data\2019\20190626\615\1-POS-3MIN.M
Analysis Method : D:\CHEM32\1\DATA\2019\20190626\615\1-POS-3MIN.M
Catalog No : HY-B0584 Batch#42692
Method Info : Mobile Phase: A: water(0.01%TFA) B:ACN(0.01%TFA)
Gradient: 5% to 95%B within 1.3 min
Flow Rate :1.8ml/min
Column :SunFire C18, 4.6*50mm,3.5um A-RP-433
Oven Temperature : 45□

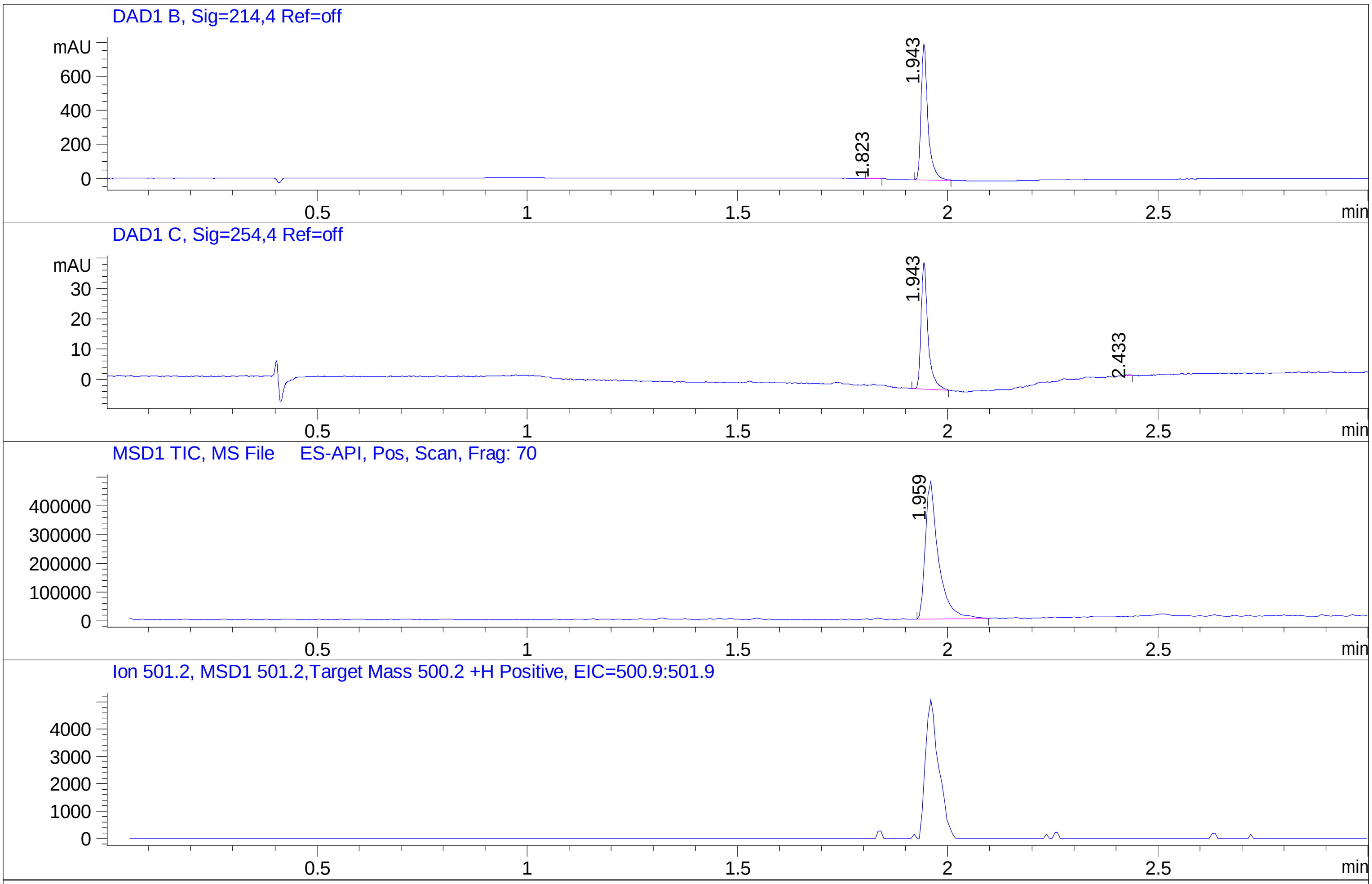

Integration Results for DAD1 B, Sig=214,4 Ref=off

RetTim	Width	Area	Height	Area%	MS(+)
1.82	0.02	1.46	1.41	0.16	ND
1.94	0.02	900.96	798.65	99.84	249

Integration Results for DAD1 C, Sig=254,4 Ref=off

RetTim	Width	Area	Height	Area%	MS(+)
1.94	0.02	47.57	41.80	99.60	249
2.43	0.01	0.19	0.23	0.40	253

Integration Results for MSD1 TIC, MS File

RetTim	Width	Area	Height	Area%	MS(+)
1.96	0.03	1040691.56	480689.25	100.00	249

Ret. Time: 1.96 <<<< POSITIVE SPECTRA >>>>

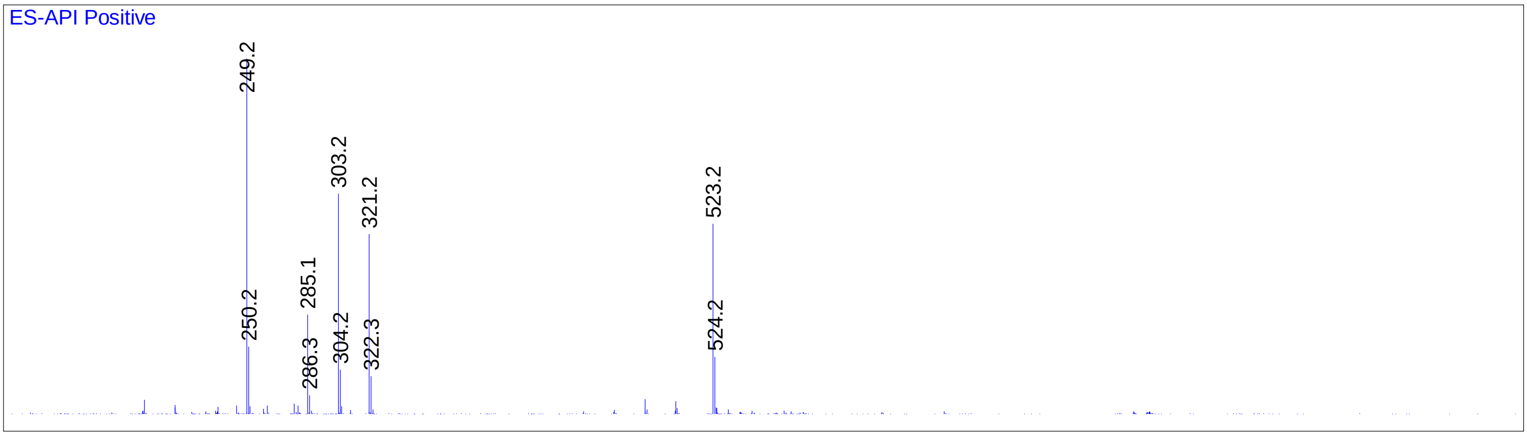

Supplement: Supplementary file 3 — cn2c00502_si_003.zip [file cn2c00502_si_003.zip › Purity_identity_files/First iteration/Molport/HY-B0584_LCMS_NMR.pdf]
