# Supplementary material for: Identification of CB1 Ligands among Drugs, Phytochemicals and Natural-Like Compounds: Virtual Screening and In Vitro Verification
Source: ACS Chem Neurosci. 2022 Oct 5;13(20):2991–3007. doi: 10.1021/acschemneuro.2c00502 (PMC9585589; doi:10.1021/acschemneuro.2c00502)
Supplement: Supplementary file 3 — cn2c00502_si_003.zip [file cn2c00502_si_003.zip › Purity_identity_files/First iteration/Molport/LHH28L5159275_AKSCI_COA.pdf]

Certificate of Analysis

AK Scientific

Iloperidone

CAS Number	133454-47-4
Catalog Number	B599
Lot Number	JL29738
Molecular Formula	$C_{24}H_{27}FN_2O_4$
Molecular Weight	426.48

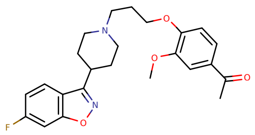

Physical and Spectral Data

Analytical Test	Results
**Appearance**	White to off-white powder
**NMR Analysis**	Consistent with structure
**Purity**	99% (HPLC)

Storage and Safety Information

**Long-term Storage**	Store in tight containers in a cool and dry place
**Retest Date**	Three years from the Release date
**Intended Use**	For laboratory research and development use only
**Safety**	See Safety Data Sheet

QC/QA Product Release Scientist: **Release Date: 1/22/2020**

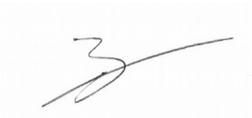

sales@aksci.com
www.aksci.com
510-429-8835

30023 Ahern Ave.
Union City, CA 94587

Certificate of Analysis

AK Scientific

Lurasidone

CAS Number	367514-87-2
Catalog Number	Y0344
Lot Number	TL36290
Molecular Formula	$C_{28}H_{36}N_4O_2S$
Molecular Weight	492.68

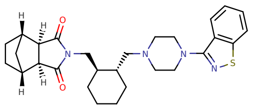

Physical and Spectral Data

Analytical Test	Results
**Appearance**	White powder
**NMR Analysis**	Consistent with structure
**Purity**	99% (HPLC)

Storage and Safety Information

**Long-term Storage**	Store in tight containers in a cool and dry place
**Retest Date**	Three years from the Release date
**Intended Use**	For laboratory research and development use only
**Safety**	See Safety Data Sheet

QC/QA Product Release Scientist: **Release Date: 8/23/2020**

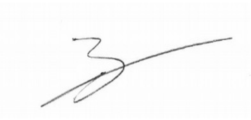

sales@aksci.com
www.aksci.com
510-429-8835

30023 Ahern Ave.
Union City, CA 94587
