# Supplementary material for: Identification of CB1 Ligands among Drugs, Phytochemicals and Natural-Like Compounds: Virtual Screening and In Vitro Verification
Source: ACS Chem Neurosci. 2022 Oct 5;13(20):2991–3007. doi: 10.1021/acschemneuro.2c00502 (PMC9585589; doi:10.1021/acschemneuro.2c00502)
Supplement: Supplementary file 3 — cn2c00502_si_003.zip [file cn2c00502_si_003.zip › Purity_identity_files/Second iteration/ChemSpace/CFN97979 HPLC.pdf]

Analysis Report

<Sample Information>

Sample Name : CFN97979
Instrument : SHIMADZU LC-20AT
Column : Wonda Cract ODS-2 (5 μm, 4.6×250 mm)
Column Temp. : 35°C
Mobile phase flow : 1.0 ml/min
Injection Volume : 5 uL
Detection Wave. : UV-270nm
Con. & Solvent : 0.4mg/ml (Methanol)

<Analysis condition>

50%→100% Acetonitrile in 0.05% Phosphoric acid H2O 10min
100%→100% Acetonitrile in 0.05% Phosphoric acid H2O 5min

<Chromatogram>

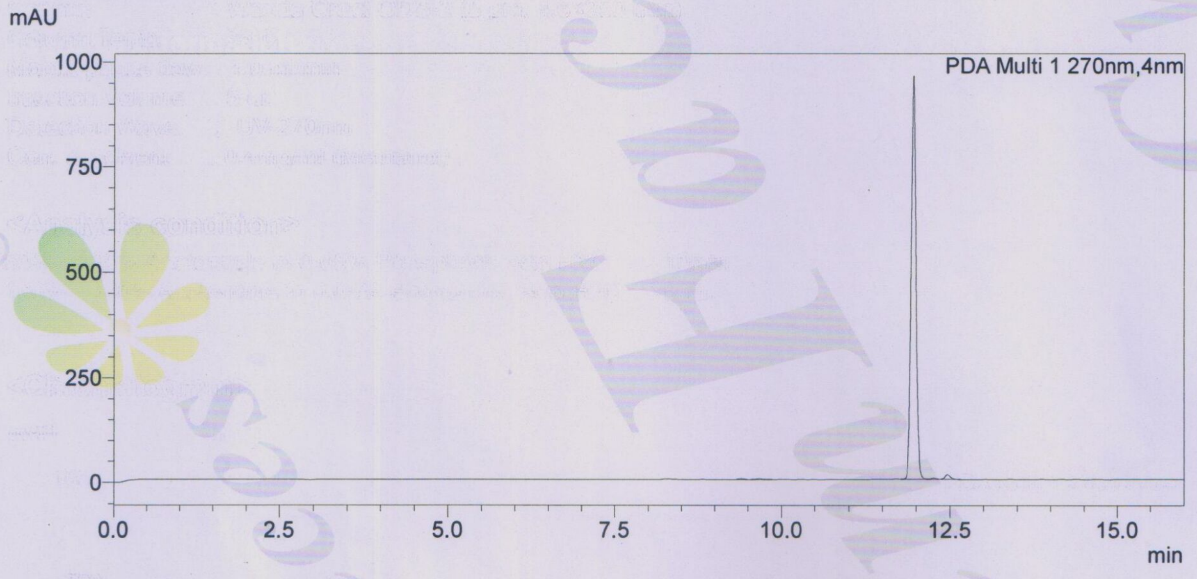

<Peak Table>

PDA Ch1 270nm

Peak#	Ret. Time	Area	Height	Conc.	Unit	Area%
1	9.661	26821	2083	0.000		0.544
2	11.958	4827473	956570	0.000		98.030
3	12.463	75195	10595	0.000		1.425
		4929489	969248			100.000
