# Supplementary material for: Identification of CB1 Ligands among Drugs, Phytochemicals and Natural-Like Compounds: Virtual Screening and In Vitro Verification
Source: ACS Chem Neurosci. 2022 Oct 5;13(20):2991–3007. doi: 10.1021/acschemneuro.2c00502 (PMC9585589; doi:10.1021/acschemneuro.2c00502)
Supplement: Supplementary file 3 — cn2c00502_si_003.zip [file cn2c00502_si_003.zip › Purity_identity_files/Second iteration/Molport/C487-0216.pdf]

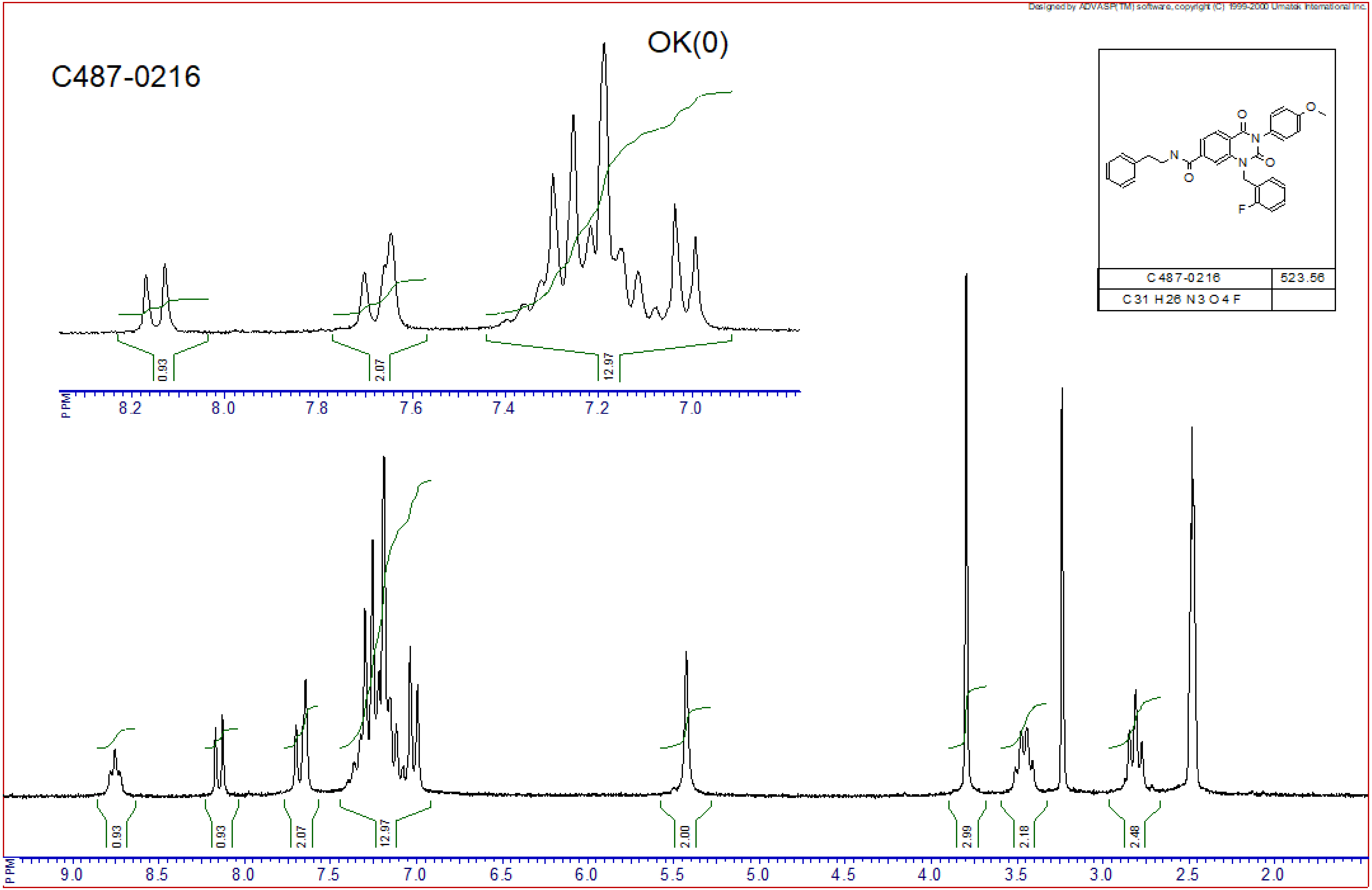

**File name: C487-0216**	Operator: MVI	SF: 199.9709 MHz	NSC: 0	PW: 5.00 usec, RG: 36	Grade:
Date: 29-Mar-2003	Solvent: DMSO	SW: 4500 Hz	TE: 313 K	AQ: 1.99 sec, RD: 0.00 sec	* C487-021629-Mar-2003
