# Supplementary material for: Identification of CB1 Ligands among Drugs, Phytochemicals and Natural-Like Compounds: Virtual Screening and In Vitro Verification
Source: ACS Chem Neurosci. 2022 Oct 5;13(20):2991–3007. doi: 10.1021/acschemneuro.2c00502 (PMC9585589; doi:10.1021/acschemneuro.2c00502)
Supplement: Supplementary file 3 — cn2c00502_si_003.zip [file cn2c00502_si_003.zip › Purity_identity_files/Second iteration/Molport/E456-2344.pdf]

E456-2344

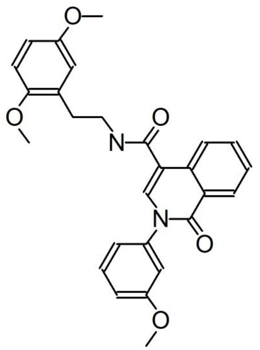

E456-2344	ML01	R311-0017_RAmi-0573
C27H26N2O5	458.51	

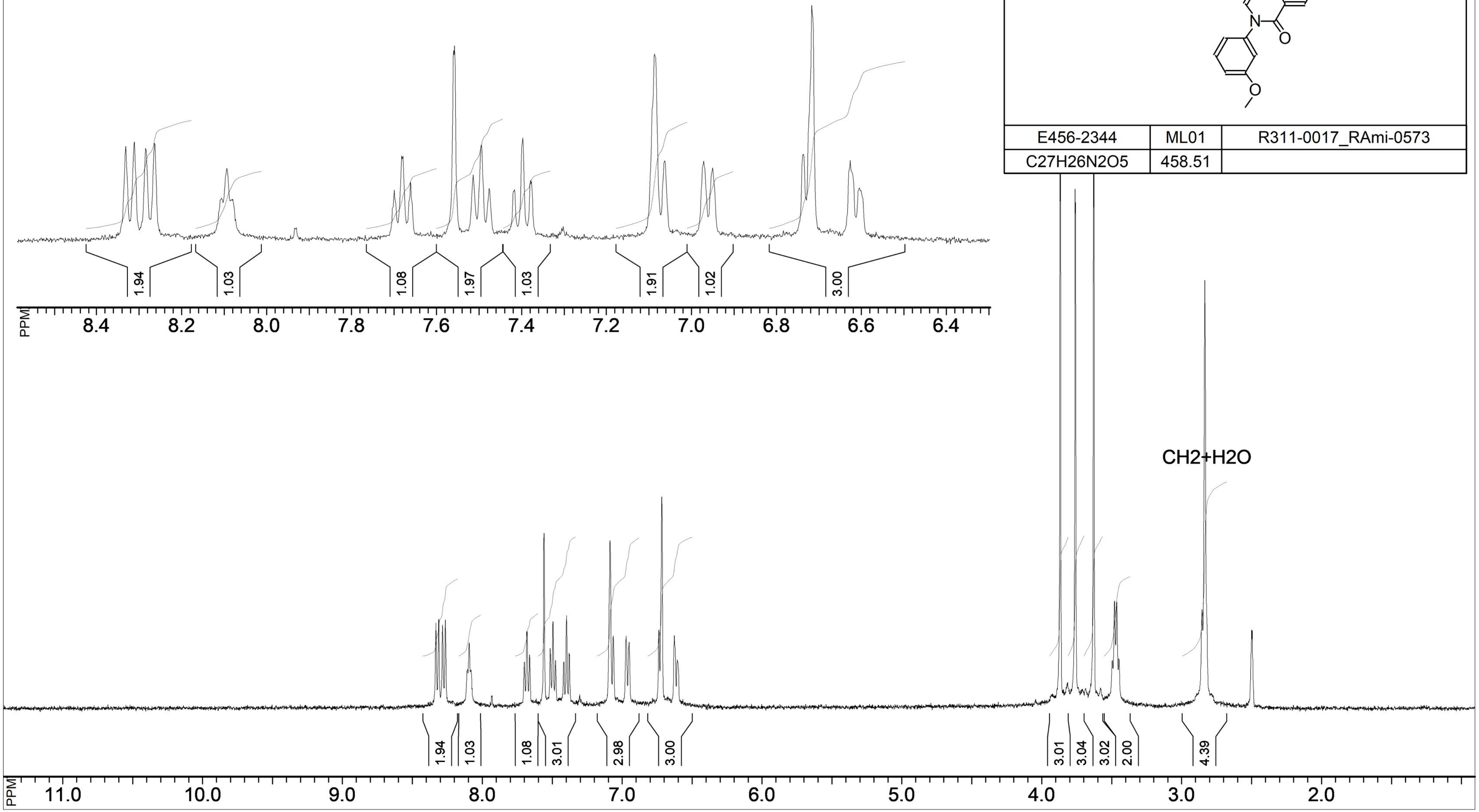

**File name: E456-2344**	Operator: PEB	SF: 399.9525 MHz	NSC: 0	PW: 12.00 usec, RG: 28, SI: 16384	**Grade: OK(00)**
Date: 08-Apr-2004	Solvent: DMSO-d6 + CCl4	SW: 7502 Hz	TE: 300 K	AQ: 1.09 sec, RD: 1.50 sec	*** E456- 2344. 08- Apr - 2004. 00***
