# Supplementary material for: Identification of CB1 Ligands among Drugs, Phytochemicals and Natural-Like Compounds: Virtual Screening and In Vitro Verification
Source: ACS Chem Neurosci. 2022 Oct 5;13(20):2991–3007. doi: 10.1021/acschemneuro.2c00502 (PMC9585589; doi:10.1021/acschemneuro.2c00502)
Supplement: Supplementary file 3 — cn2c00502_si_003.zip [file cn2c00502_si_003.zip › Purity_identity_files/Second iteration/Molport/HY-15856B_Batch#32405.pdf]

Flupentixol dihydrochloride

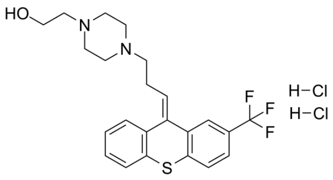

**Cat. No.:**	HY-15856B
**CAS No.:**	2413-38-9
**Molecular Formula:**	$C_{23}H_{27}Cl_2F_3N_2OS$
**Molecular Weight:**	507.44
**Target:**	Others
**Pathway:**	Others
**Storage:**	4°C, sealed storage, away from moisture and light<br>* In solvent : -80°C, 6 months; -20°C, 1 month (sealed storage, away from moisture and light)

SOLVENT & SOLUBILITY

In Vitro

H_2O : 100 mg/mL (197.07 mM; Need ultrasonic)
DMSO : 33.33 mg/mL (65.68 mM; Need ultrasonic)

Preparing Stock Solutions

Concentration / Solvent / Mass	1 mg	5 mg	10 mg
1 mM	1.9707 mL	9.8534 mL	19.7068 mL
5 mM	0.3941 mL	1.9707 mL	3.9414 mL
10 mM	0.1971 mL	0.9853 mL	1.9707 mL

Please refer to the solubility information to select the appropriate solvent.

In Vivo

1. Add each solvent one by one: 10% DMSO >> 40% PEG300 >> 5% Tween-80 >> 45% saline
   Solubility: ≥ 2.5 mg/mL (4.93 mM); Clear solution
2. Add each solvent one by one: 10% DMSO >> 90% (20% SBE-β-CD in saline)
   Solubility: ≥ 2.5 mg/mL (4.93 mM); Clear solution
3. Add each solvent one by one: 10% DMSO >> 90% corn oil
   Solubility: ≥ 2.5 mg/mL (4.93 mM); Clear solution

BIOLOGICAL ACTIVITY

Description

Flupentixol dihydrochloride, a thioxanthene drug, is used in therapy of schizophrenia as well as in anxiolytic and depressive disorders.

REFERENCES

[1]. Yonar D, et al. Effect of cis-(Z)-flupentixol on DPPC membranes in the presence and absence of cholesterol. Chem Phys Lipids. 2016 Jun;198:61-71.

Caution: Product has not been fully validated for medical applications. For research use only.

Tel: 609-228-6898 Fax: 609-228-5909 E-mail: tech@MedChemExpress.com
Address: 1 Deer Park Dr, Suite Q, Monmouth Junction, NJ 08852, USA

MCE MedChemExpress

Certificate of Analysis

Inhibitors • Screening Libraries • Proteins

Flupentixol dihydrochloride

Cat. No.: HY-15856B

CAS No.: 2413-38-9

Batch No.: 32405

Chemical Name: 1-Piperazineethanol, 4-[3-[2-(trifluoromethyl)-9H-thioxanthen-9-ylidene]propyl]-, hydrochloride (1:2)

PHYSICAL AND CHEMICAL PROPERTIES

Molecular Formula: $C_{23}H_{27}Cl_2F_3N_2OS$

Molecular Weight: 507.44

Storage: 4°C, sealed storage, away from moisture and light

* In solvent : -80°C, 6 months; -20°C, 1 month (sealed storage, away from moisture and light)

Chemical Structure:

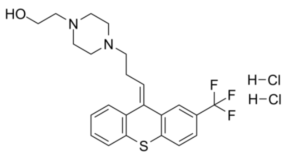

ANALYTICAL DATA

Appearance: White to off-white (Solid)

^{1}H NMR Spectrum: Consistent with structure

LCMS: Consistent with structure

Purity (LCMS): 99.73%

Conclusion: The product has been tested and complies with the given specifications.

Caution: Product has not been fully validated for medical applications. For research use only.

Tel: 609-228-6898 Fax: 609-228-5909 E-mail: tech@MedChemExpress.com
Address: 1 Deer Park Dr, Suite Q, Monmouth Junction, NJ 08852, USA

Revision Date: Aug.-31-2018
Print Date: May.-11-2022

1. PRODUCT AND COMPANY IDENTIFICATION

1.1 Product identifier

Product name : Flupentixol dihydrochloride

Catalog No. : HY-15856B

CAS No. : 2413-38-9

1.2 Relevant identified uses of the substance or mixture and uses advised against

Identified uses : Laboratory chemicals, manufacture of substances.

1.3 Details of the supplier of the safety data sheet

Company: MedChemExpress USA

Tel: 609-228-6898

Fax: 609-228-5909

E-mail: sales@medchemexpress.com

1.4 Emergency telephone number

Emergency Phone #: 609-228-6898

2. HAZARDS IDENTIFICATION

2.1 Classification of the substance or mixture

Not a hazardous substance or mixture.

2.2 GHS Label elements, including precautionary statements

Not a hazardous substance or mixture.

2.3 Other hazards

None.

3. COMPOSITION/INFORMATION ON INGREDIENTS

3.1 Substances

Synonyms: Flupenthixol dihydrochloride

Formula: $C_{23}H_{27}Cl_2F_3N_2OS$

Molecular Weight: 507.44

CAS No. : 2413-38-9

4. FIRST AID MEASURES

4.1 Description of first aid measures

Eye contact

Remove any contact lenses, locate eye-wash station, and flush eyes immediately with large amounts of water. Separate eyelids with fingers to ensure adequate flushing. Promptly call a physician.

Skin contact

Rinse skin thoroughly with large amounts of water. Remove contaminated clothing and shoes and call a physician.

Inhalation

Immediately relocate self or casualty to fresh air. If breathing is difficult, give cardiopulmonary resuscitation (CPR). Avoid mouth-to-mouth resuscitation.

Ingestion

Wash out mouth with water; Do NOT induce vomiting; call a physician.

4.2 Most important symptoms and effects, both acute and delayed

The most important known symptoms and effects are described in the labelling (see section 2.2).

4.3 Indication of any immediate medical attention and special treatment needed

Treat symptomatically.

5. FIRE FIGHTING MEASURES

5.1 Extinguishing media

Suitable extinguishing media

Use water spray, dry chemical, foam, and carbon dioxide fire extinguisher.

5.2 Special hazards arising from the substance or mixture

During combustion, may emit irritant fumes.

5.3 Advice for firefighters

Wear self-contained breathing apparatus and protective clothing.

6. ACCIDENTAL RELEASE MEASURES

6.1 Personal precautions, protective equipment and emergency procedures

Use full personal protective equipment. Avoid breathing vapors, mist, dust or gas. Ensure adequate ventilation. Evacuate personnel to safe areas.

Refer to protective measures listed in sections 8.

6.2 Environmental precautions

Try to prevent further leakage or spillage. Keep the product away from drains or water courses.

6.3 Methods and materials for containment and cleaning up

Absorb solutions with finely-powdered liquid-binding material (diatomite, universal binders); Decontaminate surfaces and equipment by scrubbing with alcohol; Dispose of contaminated material according to Section 13.

7. HANDLING AND STORAGE

7.1 Precautions for safe handling

Avoid inhalation, contact with eyes and skin. Avoid dust and aerosol formation. Use only in areas with appropriate exhaust ventilation.

7.2 Conditions for safe storage, including any incompatibilities

Keep container tightly sealed in cool, well-ventilated area. Keep away from direct sunlight and sources of ignition.

Recommended storage temperature:	4°C, sealed storage, away from moisture and light<br>* In solvent : -80°C, 6 months; -20°C, 1 month (sealed storage, away from moisture and light)

Shipping at room temperature if less than 2 weeks.

7.3 Specific end use(s)

No data available.

8. EXPOSURE CONTROLS/PERSONAL PROTECTION

8.1 Control parameters

Components with workplace control parameters

This product contains no substances with occupational exposure limit values.

8.2 Exposure controls

Engineering controls

Ensure adequate ventilation. Provide accessible safety shower and eye wash station.

Personal protective equipment

**Eye protection**	Safety goggles with side-shields.
**Hand protection**	Protective gloves.
**Skin and body protection**	Impervious clothing.
**Respiratory protection**	Suitable respirator.
**Environmental exposure controls**	Keep the product away from drains, water courses or the soil. Clean spillages in a safe way as soon as possible.

9. PHYSICAL AND CHEMICAL PROPERTIES

9.1 Information on basic physical and chemical properties

**Appearance**	Solid
**Odor**	No data available
**Odor threshold**	No data available
**pH**	No data available
**Melting/freezing point**	No data available
**Boiling point/range**	No data available
**Flash point**	No data available
**Evaporation rate**	No data available
**Flammability (solid, gas)**	No data available
**Upper/lower flammability or explosive limits**	No data available
**Vapor pressure**	No data available
**Vapor density**	No data available
**Relative density**	No data available
**Water Solubility**	No data available
**Partition coefficient**	No data available
**Auto-ignition temperature**	No data available
**Decomposition temperature**	No data available
**Viscosity**	No data available
**Explosive properties**	No data available
**Oxidizing properties**	No data available

9.2 Other safety information

No data available.

10. STABILITY AND REACTIVITY

10.1 Reactivity

No data available.

10.2 Chemical stability

Stable under recommended storage conditions.

10.3 Possibility of hazardous reactions

No data available.

10.4 Conditions to avoid

No data available.

10.5 Incompatible materials

Strong acids/alkalis, strong oxidising/reducing agents.

10.6 Hazardous decomposition products

Under fire conditions, may decompose and emit toxic fumes.

Other decomposition products - no data available.

11.TOXICOLOGICAL INFORMATION

11.1 Information on toxicological effects

Acute toxicity

Classified based on available data. For more details, see section 2

Skin corrosion/irritation

Classified based on available data. For more details, see section 2

Serious eye damage/irritation

Classified based on available data. For more details, see section 2

Respiratory or skin sensitization

Classified based on available data. For more details, see section 2

Germ cell mutagenicity

Classified based on available data. For more details, see section 2

Carcinogenicity

IARC: No component of this product present at a level equal to or greater than 0.1% is identified as probable, possible or confirmed human carcinogen by IARC.

ACGIH: No component of this product present at a level equal to or greater than 0.1% is identified as a potential or confirmed carcinogen by ACGIH.

NTP: No component of this product present at a level equal to or greater than 0.1% is identified as a anticipated or confirmed carcinogen by NTP.

OSHA: No component of this product present at a level equal to or greater than 0.1% is identified as a potential or confirmed carcinogen by OSHA.

Reproductive toxicity

Classified based on available data. For more details, see section 2

Specific target organ toxicity - single exposure

Classified based on available data. For more details, see section 2

Specific target organ toxicity - repeated exposure

Classified based on available data. For more details, see section 2

Aspiration hazard

Classified based on available data. For more details, see section 2

Additional information

This information is based on our current knowledge. However the chemical, physical, and toxicological properties have not been completely investigated.

12. ECOLOGICAL INFORMATION

12.1 Toxicity

No data available.

12.2 Persistence and degradability

No data available.

12.3 Bioaccumlative potential

No data available.

12.4 Mobility in soil

No data available.

12.5 Results of PBT and vPvB assessment

PBT/vPvB assessment unavailable as chemical safety assessment not required or not conducted.

12.6 Other adverse effects

No data available.

13. DISPOSAL CONSIDERATIONS

13.1 Waste treatment methods

Product

Dispose substance in accordance with prevailing country, federal, state and local regulations.

Contaminated packaging

Conduct recycling or disposal in accordance with prevailing country, federal, state and local regulations.

14. TRANSPORT INFORMATION

DOT (US)

Proper shipping name: Not dangerous goods

UN number: -

Class: -

Packing group: -

IMDG

Proper shipping name: Not dangerous goods

UN number: -

Class: -

Packing group: -

IATA

Proper shipping name: Not dangerous goods

UN number: -

Class: -

Packing group: -

15. REGULATORY INFORMATION

SARA 302 Components:

No chemicals in this material are subject to the reporting requirements of SARA Title III, Section 302.

SARA 313 Components:

This material does not contain any chemical components with known CAS numbers that exceed the threshold (De Minimis) reporting levels established by SARA Title III, Section 313.

SARA 311/312 Hazards:

No SARA Hazards.

Massachusetts Right To Know Components:

No components are subject to the Massachusetts Right to Know Act.

Pennsylvania Right To Know Components:

No components are subject to the Pennsylvania Right to Know Act.

New Jersey Right To Know Components:

No components are subject to the New Jersey Right to Know Act.

California Prop. 65 Components:

This product does not contain any chemicals known to State of California to cause cancer, birth defects, or anyother reproductive harm.

16. OTHER INFORMATION

Copyright 2022 MedChemExpress. The above information is correct to the best of our present knowledge but does not purport to be all inclusive and should be used only as a guide. The product is for research use only and for experienced personnel. It must only be handled by suitably qualified experienced scientists in appropriately equipped and authorized facilities. The burden of safe use of this material rests entirely with the user. MedChemExpress disclaims all liability for any damage resulting from handling or from contact with this product.

Tel: 609-228-6898 Fax: 609-228-5909 E-mail: tech@MedChemExpress.com

Address: 1 Deer Park Dr, Suite Q, Monmouth Junction, NJ 08852, USA

MS Report from Instrument: LCMS-02

File ..1\DATA\2018\20180827\371\BIZ2018-827-WYQ6.D Tgt Mass (CHM):
Injection Date : 27 Aug 18 5:09 pm +0800 Seq. Line : 16
Sample Name : BIZ2018-827-WYQ6 Location : P1-D-01
Acq. Operator : LQ_1052 Inj : 1
Spec. Reported : MS Integration Inj Volume : 1 ul
Acq. Method : D:\Chem32\1\data\2018\20180827\371\1-POS-3MIN-1.M
Analysis Method : D:\CHEM32\1\DATA\2018\20180827\371\1-POS-3MIN-1.M
Catalog No: HY-15856B Batch#32405 A-RP-314
Method Info : Mobile Phase: A: water(0.01%TFA) B:ACN(0.01%TFA)
Gradient: 5% to 95%B within 1.3 min
Flow Rate :1.8ml/min
Column :SunFire C18, 4.6*50mm,3.5um A-RP-314
Oven Temperature : 45□

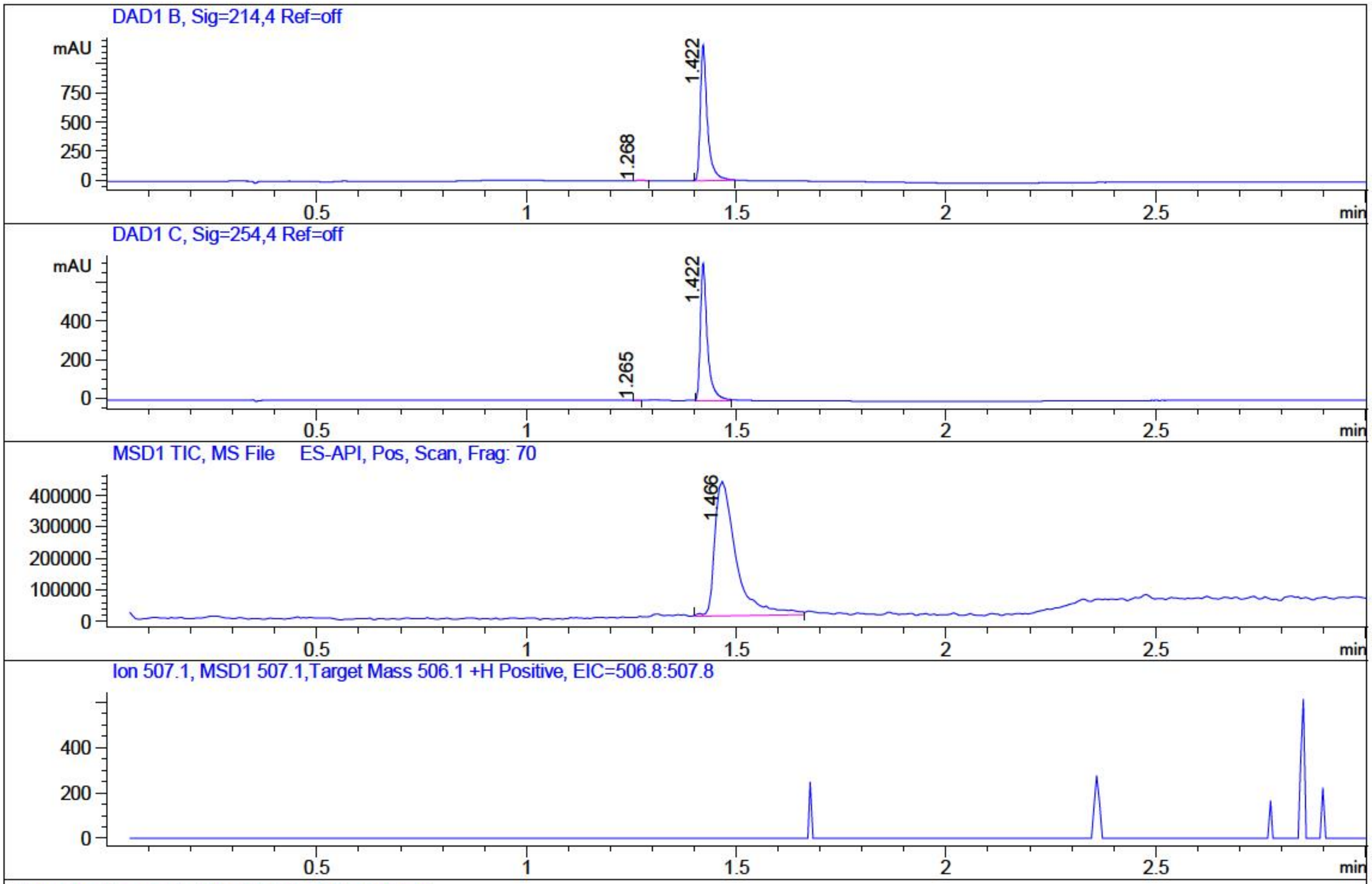

Integration Results for DAD1 B, Sig=214,4 Ref=off

RetTim	Width	Area	Height	Area%	MS(+)
1.27	0.02	3.75	3.16	0.27	ND
1.42	0.02	1366.13	1168.86	99.73	435

Integration Results for DAD1 C, Sig=254,4 Ref=off

RetTim	Width	Area	Height	Area%	MS(+)
1.26	0.01	0.31	0.52	0.04	ND
1.42	0.02	819.22	710.49	99.96	435

Integration Results for MSD1 TIC, MS File

RetTim	Width	Area	Height	Area%	MS(+)
1.47	0.05	1505929.88	427416.75	100.00	435

Ret. Time: 1.47 <<<< POSITIVE SPECTRA >>>>

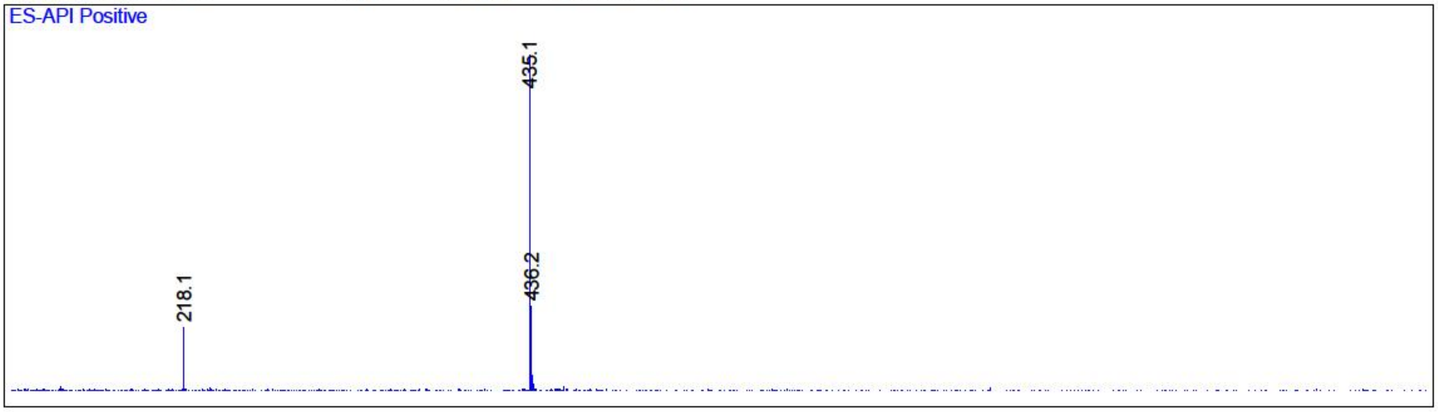

Sample ID: BIZ2018-827-WYQ6-1, Catalog No: HY-15856B Batch#32405, DMSO

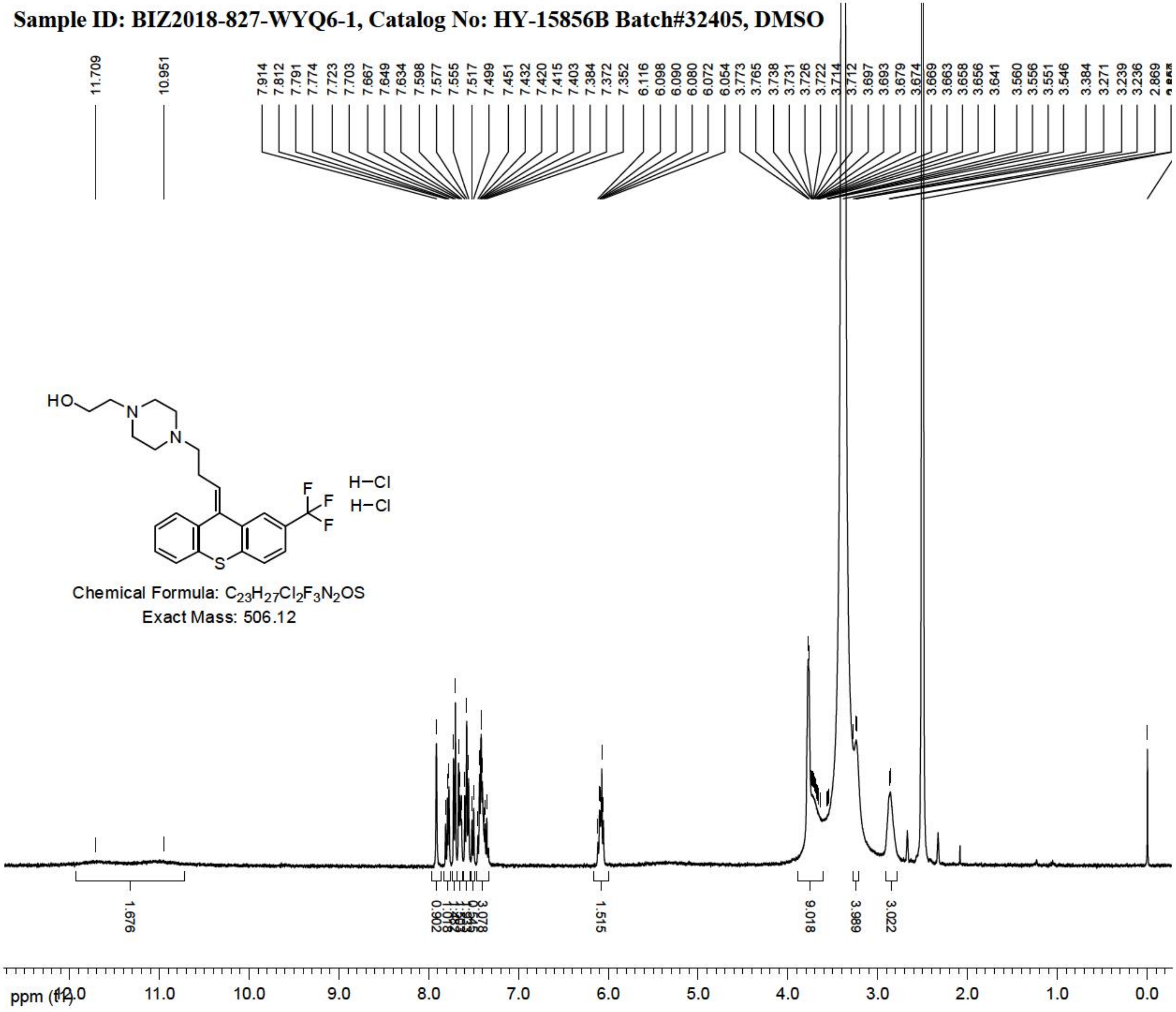

Chemical Formula: $C_{23}H_{27}Cl_2F_3N_2OS$

Exact Mass: 506.12

MCE® MedChem Express

Date:
31 Aug 2018

Document's Title:
Catalog No: HY-15856B Batch#32405

Spectrum Title:
biz2018-827-wyq6-1-dmso-180828

Frequency (MHz):
(f1) 400.130

Original Points Count:
(f1) 32768

Actual Points Count:
(f1) 32768

Acquisition Time (sec):
(f1) 3.2768

Spectral Width (ppm):
(f1) 24.992

Pulse Program:
Unknown
