# Supplementary material for: Identification of CB1 Ligands among Drugs, Phytochemicals and Natural-Like Compounds: Virtual Screening and In Vitro Verification
Source: ACS Chem Neurosci. 2022 Oct 5;13(20):2991–3007. doi: 10.1021/acschemneuro.2c00502 (PMC9585589; doi:10.1021/acschemneuro.2c00502)
Supplement: Supplementary file 3 — cn2c00502_si_003.zip [file cn2c00502_si_003.zip › Purity_identity_files/Second iteration/Molport/K784-2231.pdf]

# K784-2231

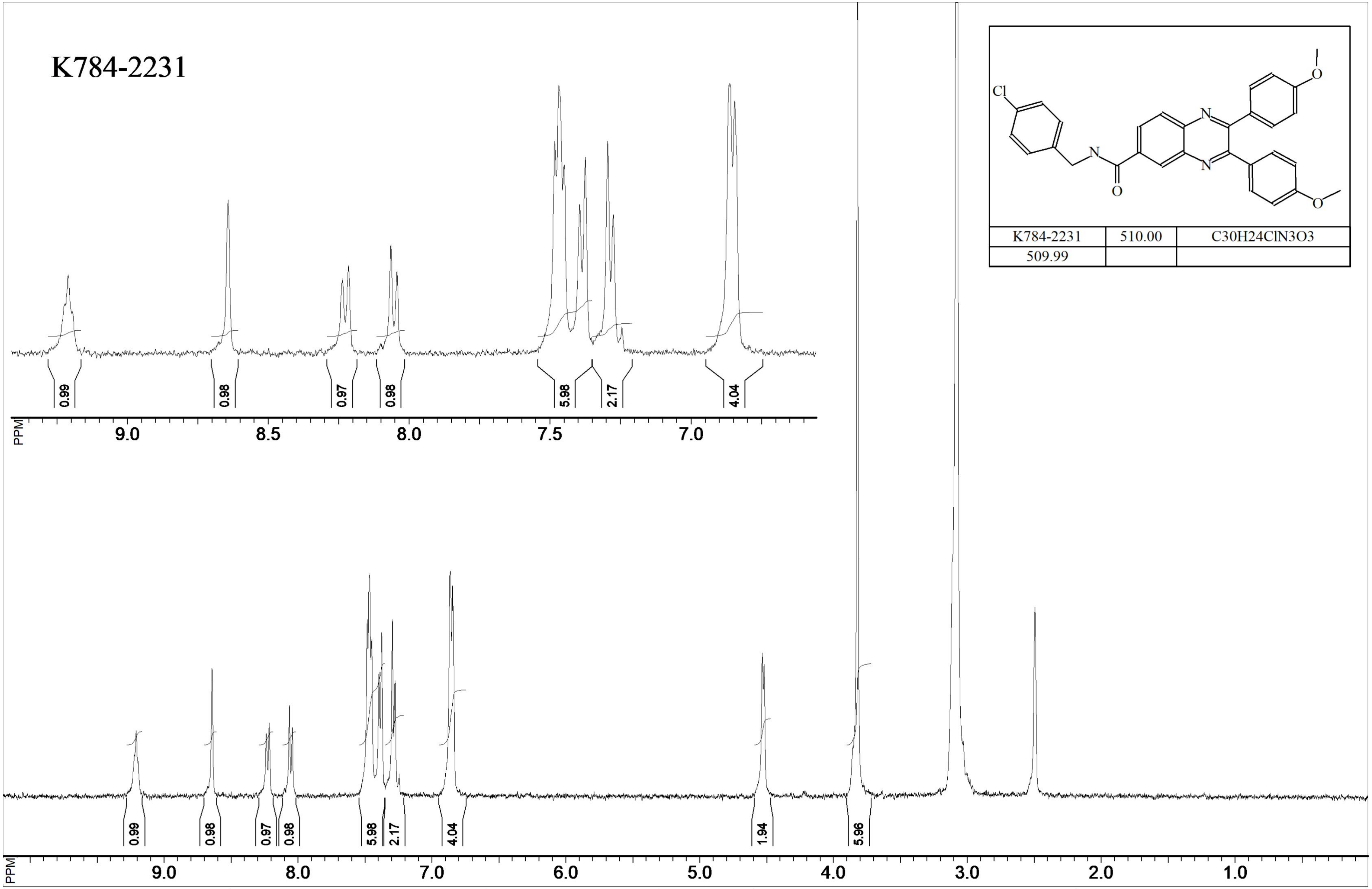


| | | |
|---|---|---|
| K784-2231 | 510.00 | C30H24ClN3O3 |
| 509.99 | | |

| | | | | | |
|---|---|---|---|---|---|
| **File name: K784-2231** | Operator: NVV, Expert: NVV | SF: 399.9525 MHz | NSC: 12 | PW: 10.00 usec, RG: 26, SI: 16384 | **Grade: OK(00)** |
| Date: 20-Jul-2001 | Solvent: DMSO-d6 + CCl4 | SW: 7502 Hz | TE: 300 K | AQ: 1.09 sec, RD: 1.50 sec | * K784- 2231. 20- Jul - 2001. 00 |
