# Supplementary material for: Identification of CB1 Ligands among Drugs, Phytochemicals and Natural-Like Compounds: Virtual Screening and In Vitro Verification
Source: ACS Chem Neurosci. 2022 Oct 5;13(20):2991–3007. doi: 10.1021/acschemneuro.2c00502 (PMC9585589; doi:10.1021/acschemneuro.2c00502)
Supplement: Supplementary file 3 — cn2c00502_si_003.zip [file cn2c00502_si_003.zip › Purity_identity_files/Second iteration/Molport/L881-0820.pdf]

L881-0820

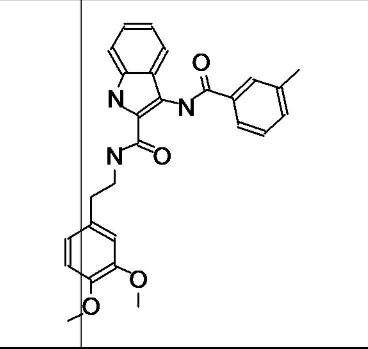

C27H27N3O4	457.52098
L881-0820	

Grade: OK(0)

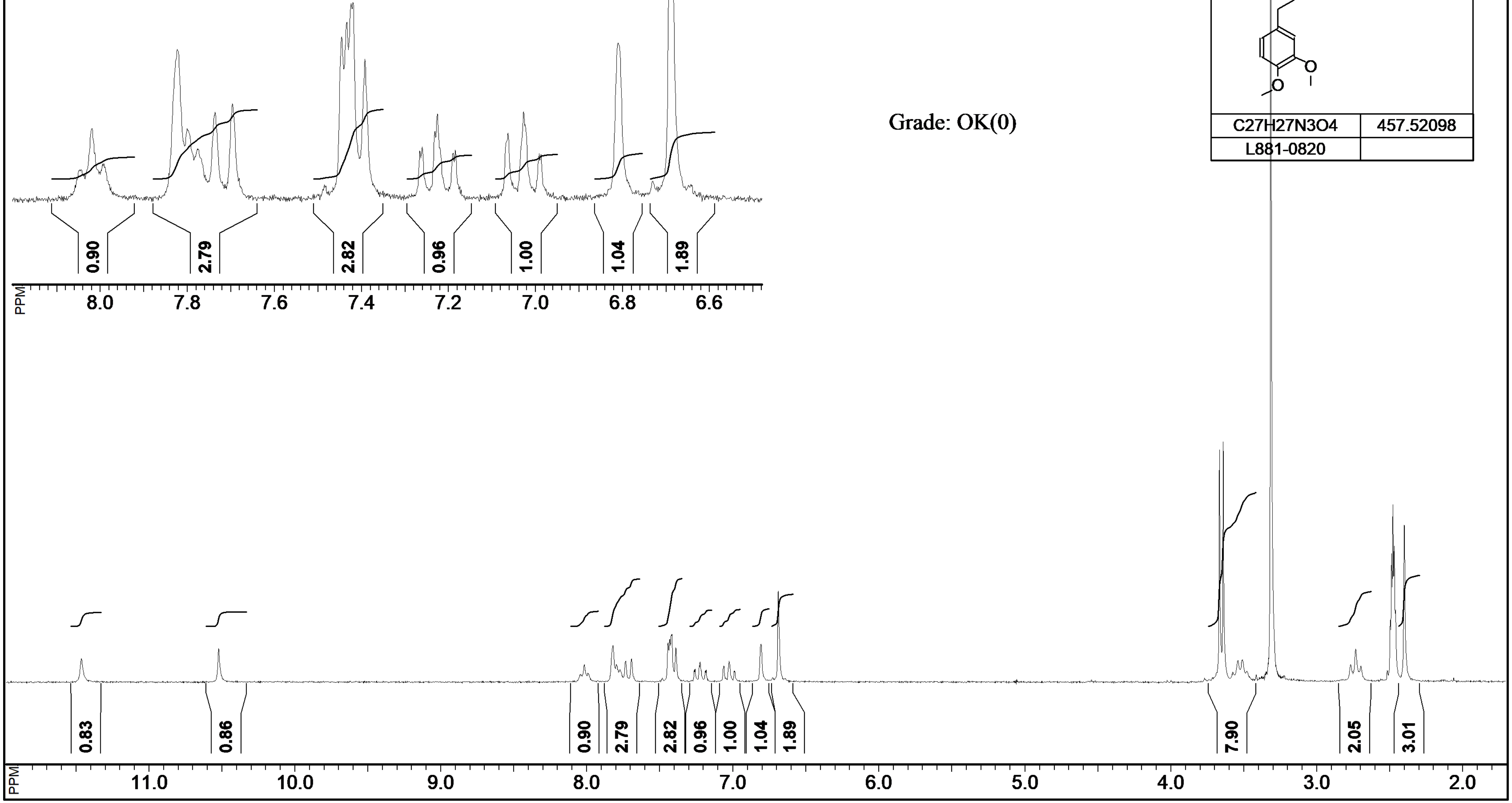

**File name: L881-0820**	Operator: MVI	SF: 199.9704 MHz	NSC: 0	PW: 4.00 usec, RG: 26	Grade: OK(0)
Date: 08-Sep-2007	Solvent: DMSO	SW: 5000 Hz	TE: 333 K	AQ: 2.00 sec, RD: 0.00 sec	L881-082008-Sep-2007
