# Supplementary material for: Identification of CB1 Ligands among Drugs, Phytochemicals and Natural-Like Compounds: Virtual Screening and In Vitro Verification
Source: ACS Chem Neurosci. 2022 Oct 5;13(20):2991–3007. doi: 10.1021/acschemneuro.2c00502 (PMC9585589; doi:10.1021/acschemneuro.2c00502)
Supplement: Supplementary file 3 — cn2c00502_si_003.zip [file cn2c00502_si_003.zip › Purity_identity_files/Second iteration/Molport/Spectra_IBScreen/STOCK1N-51151.pdf]

STRUCTURE

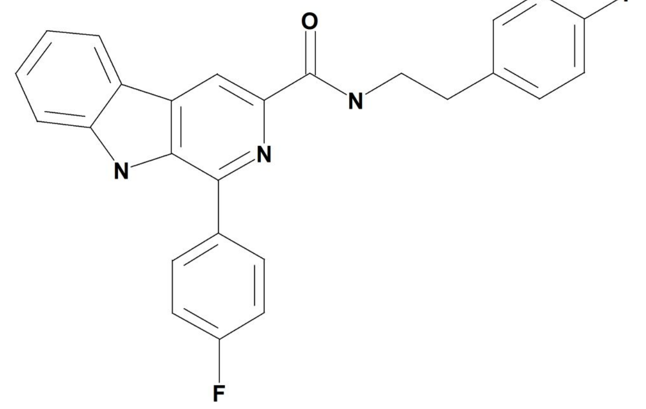


| ID1 | F: | MW: |
|---|---|---|
| **STOCK1N-51151** | $C_{26}H_{19}F_2N_3O$ | **427.46** |

| Com: | Saltdata: | ID1 | EXP16Exi003054 |
|---|---|---|---|

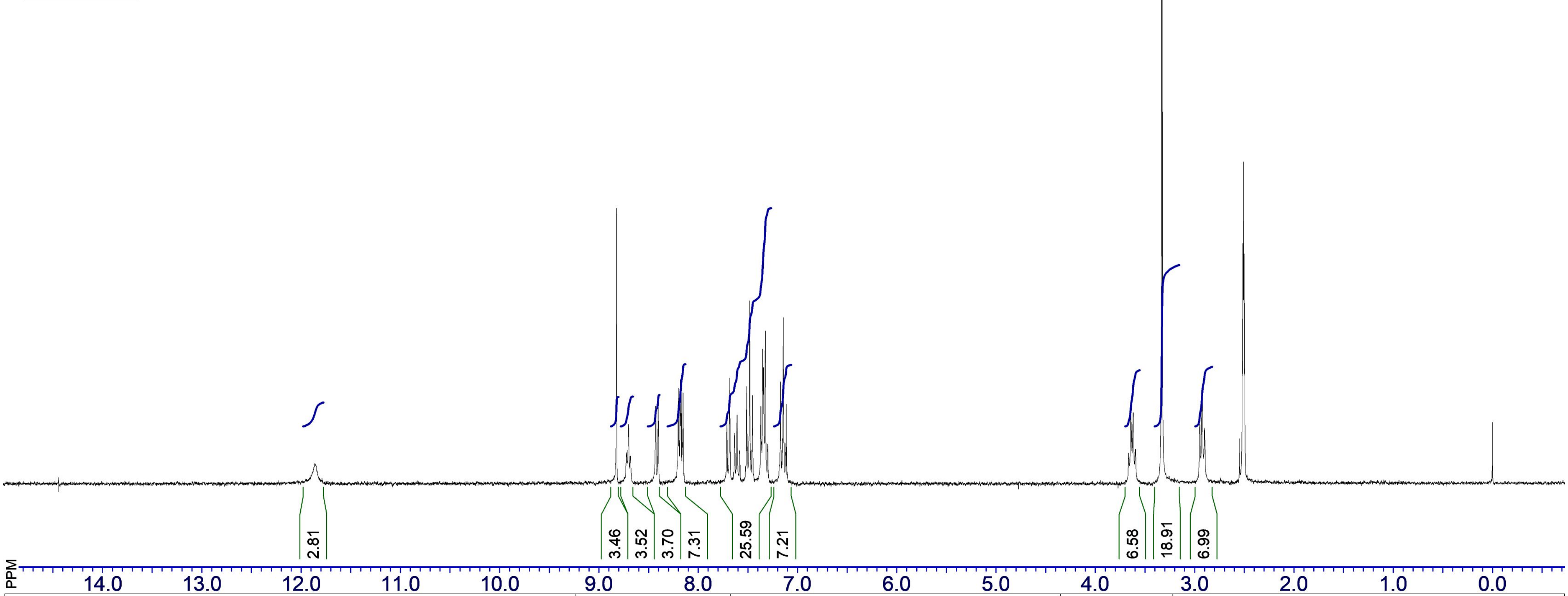


| **File name: EXP16Exi003054.nmr** | Owner: | SF: 299.9450 MHz | NS: -1 | SI: 16384, TD: 32768 |
|---|---|---|---|---|
| Date: | Solvent: | SW: 5099 | TE: 0 | SPE in DMSO-D6 |
