# Supplementary material for: Identification of CB1 Ligands among Drugs, Phytochemicals and Natural-Like Compounds: Virtual Screening and In Vitro Verification
Source: ACS Chem Neurosci. 2022 Oct 5;13(20):2991–3007. doi: 10.1021/acschemneuro.2c00502 (PMC9585589; doi:10.1021/acschemneuro.2c00502)
Supplement: Supplementary file 3 — cn2c00502_si_003.zip [file cn2c00502_si_003.zip › Purity_identity_files/Second iteration/Molport/Spectra_IBScreen/STOCK1N-84571.pdf]

STRUCTURE

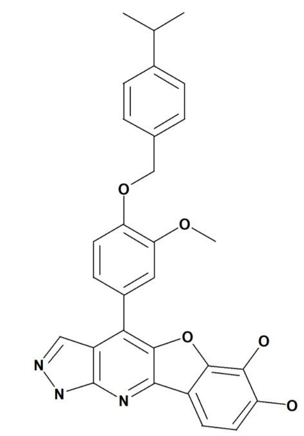

ID1	F:	MW:
**STOCK1N-84571**	$C_{29}H_{25}N_3O_5$	**495.54**

Com:	Saltdata:	ID1
		MAVAS-135405

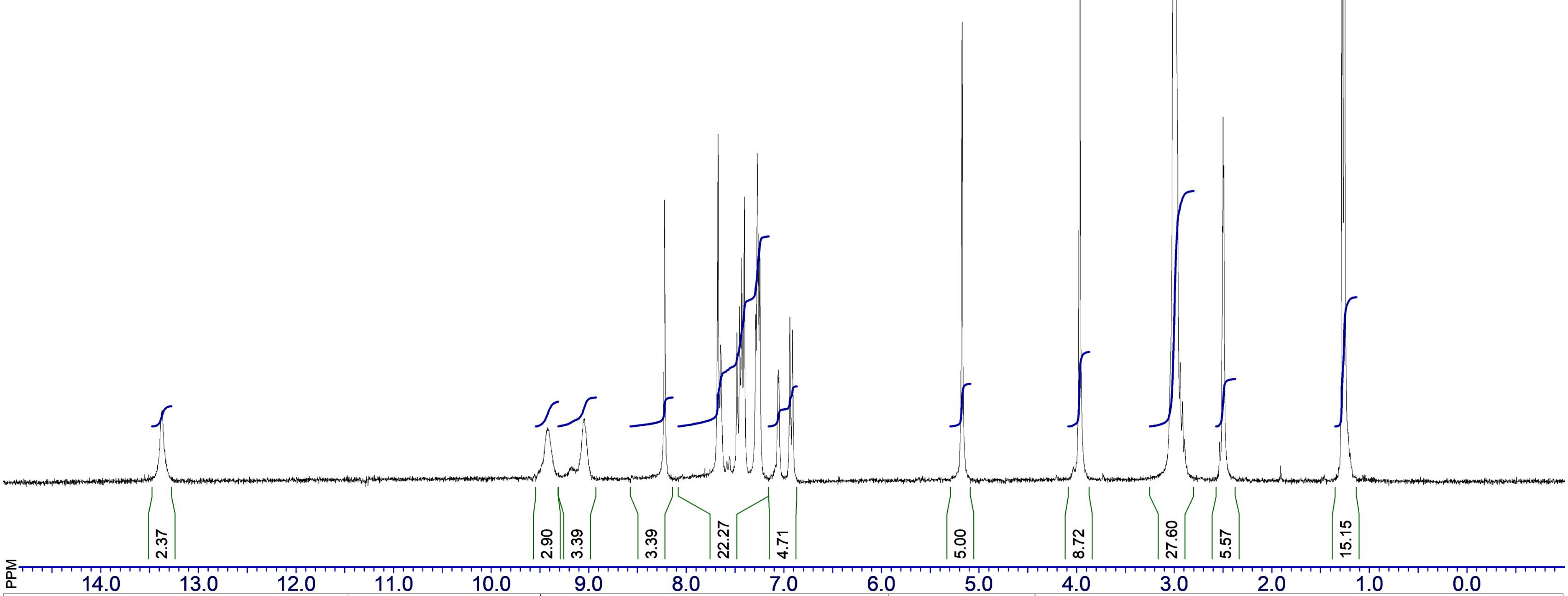

**File name: G52097**	Owner:	SF: 300.1416 MHz	NS: 16	SI: 8192, TD: 14336
Date: 21-Apr-2014	Solvent:	SW: 5376	TE: 318	/ksia g52097 mavas-135405
