# Supplementary material for: Identification of CB1 Ligands among Drugs, Phytochemicals and Natural-Like Compounds: Virtual Screening and In Vitro Verification
Source: ACS Chem Neurosci. 2022 Oct 5;13(20):2991–3007. doi: 10.1021/acschemneuro.2c00502 (PMC9585589; doi:10.1021/acschemneuro.2c00502)
Supplement: Supplementary file 3 — cn2c00502_si_003.zip [file cn2c00502_si_003.zip › Purity_identity_files/Second iteration/Molport/Spectra_IBScreen/STOCK1N-91882.pdf]

STRUCTURE

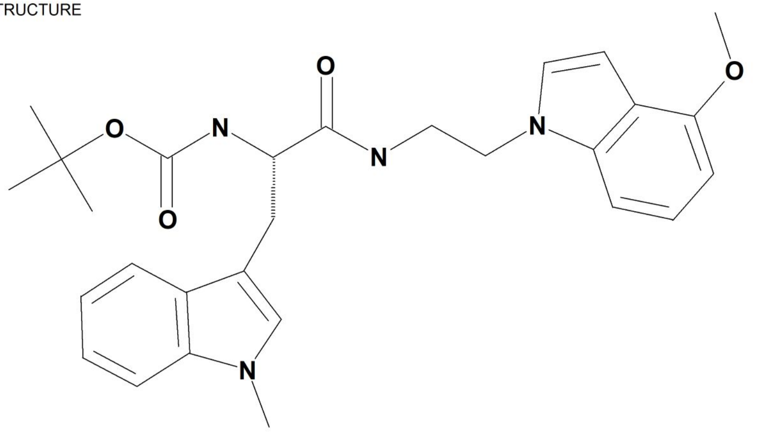

ID1	F:	MW:
**STOCK1N-91882**	$C_{28}H_{34}N_4O_4$	**490.61**

Com:	Saltdata:	ID1
		ExLab15-002501

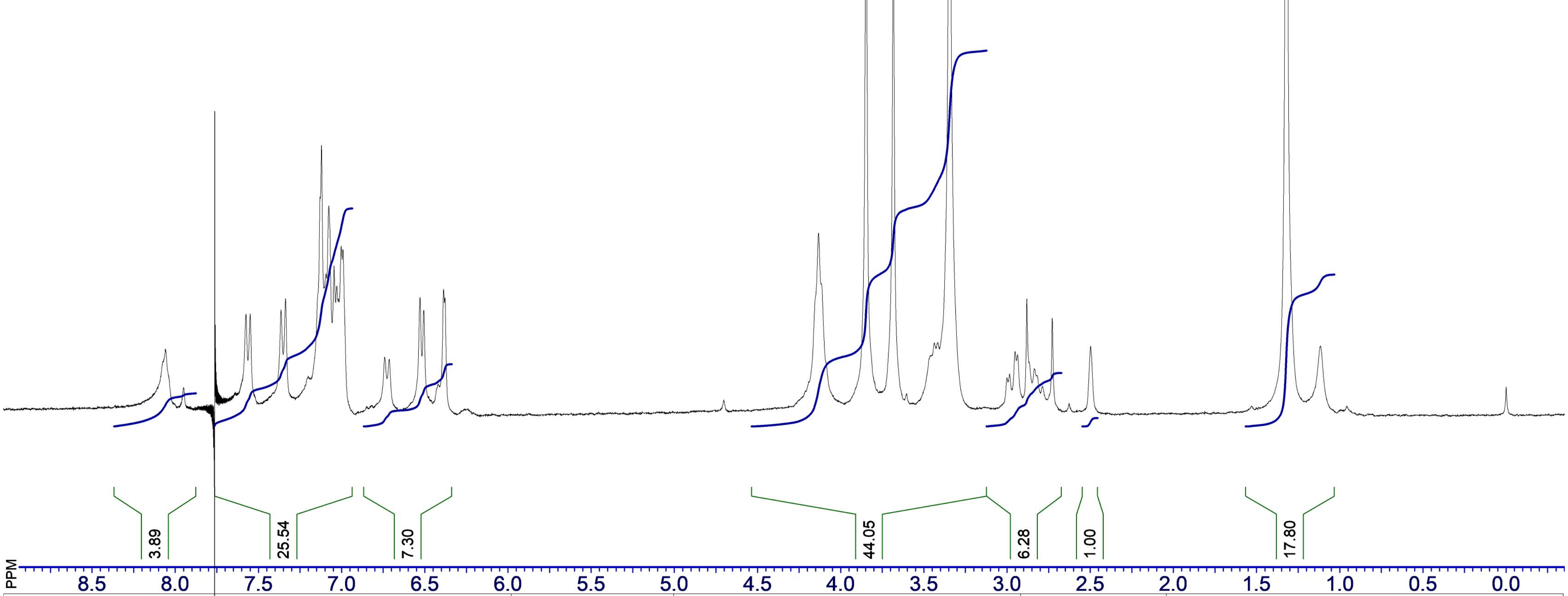

**File name: ExLab15-002501**	Owner:	SF: 299.9450 MHz	NS: 18	SI: 32768, TD: 17472
Date: 28-Sep-2015	Solvent:	SW: 5099	TE: 300	
