# Supplementary material for: Identification of CB1 Ligands among Drugs, Phytochemicals and Natural-Like Compounds: Virtual Screening and In Vitro Verification
Source: ACS Chem Neurosci. 2022 Oct 5;13(20):2991–3007. doi: 10.1021/acschemneuro.2c00502 (PMC9585589; doi:10.1021/acschemneuro.2c00502)
Supplement: Supplementary file 3 — cn2c00502_si_003.zip [file cn2c00502_si_003.zip › Purity_identity_files/Second iteration/Molport/Spectra_IBScreen/STOCK4S-74545.pdf]

STRUCTURE

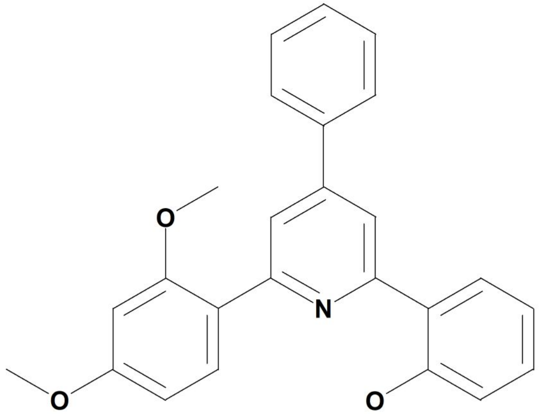

ID1	F:	MW:
STOCK4S-74545	$C_{25}H_{21}NO_3$	383.45

Com:	Saltdata:	ID1	RRKI-250

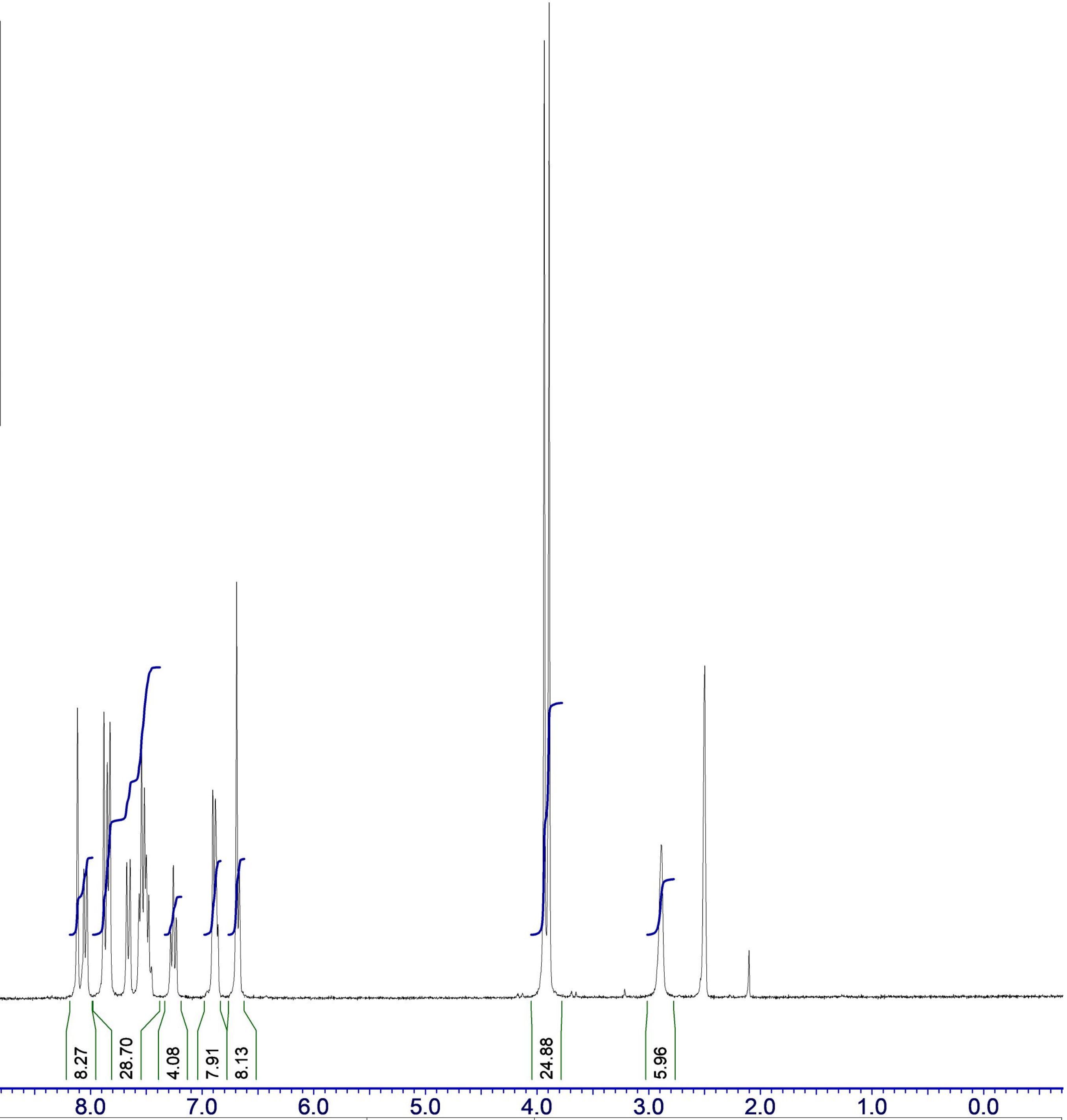

**File name: D31502**	Owner:	SF: 300.1415 MHz	NS: 16	SI: 8192, TD: 14336
Date:	Solvent:	SW: 5376	TE: 318	/KSIA D31502 RRKI-250
