# Supplementary material for: Identification of CB1 Ligands among Drugs, Phytochemicals and Natural-Like Compounds: Virtual Screening and In Vitro Verification
Source: ACS Chem Neurosci. 2022 Oct 5;13(20):2991–3007. doi: 10.1021/acschemneuro.2c00502 (PMC9585589; doi:10.1021/acschemneuro.2c00502)
Supplement: Supplementary file 3 — cn2c00502_si_003.zip [file cn2c00502_si_003.zip › Purity_identity_files/Second iteration/Molport/T2382_132640_HPLC.pdf]

TargetMol
A DRUG SCREENING EXPERT

SAMPLE INFORMATION

Sample Name:	132640-1	Acquired By:	System
Sample Type:	Unknown		
Vial:	10	Acq. Method Set:	B70D30
Injection #:	1	Processing Method	132540z
Injection Volume:	5.00 ul	Channel Name:	254.0nm
Run Time:	20.0 Minutes	Proc. Chnl. Descr.:	PDA 254.0 nm (PDA 190.0 to

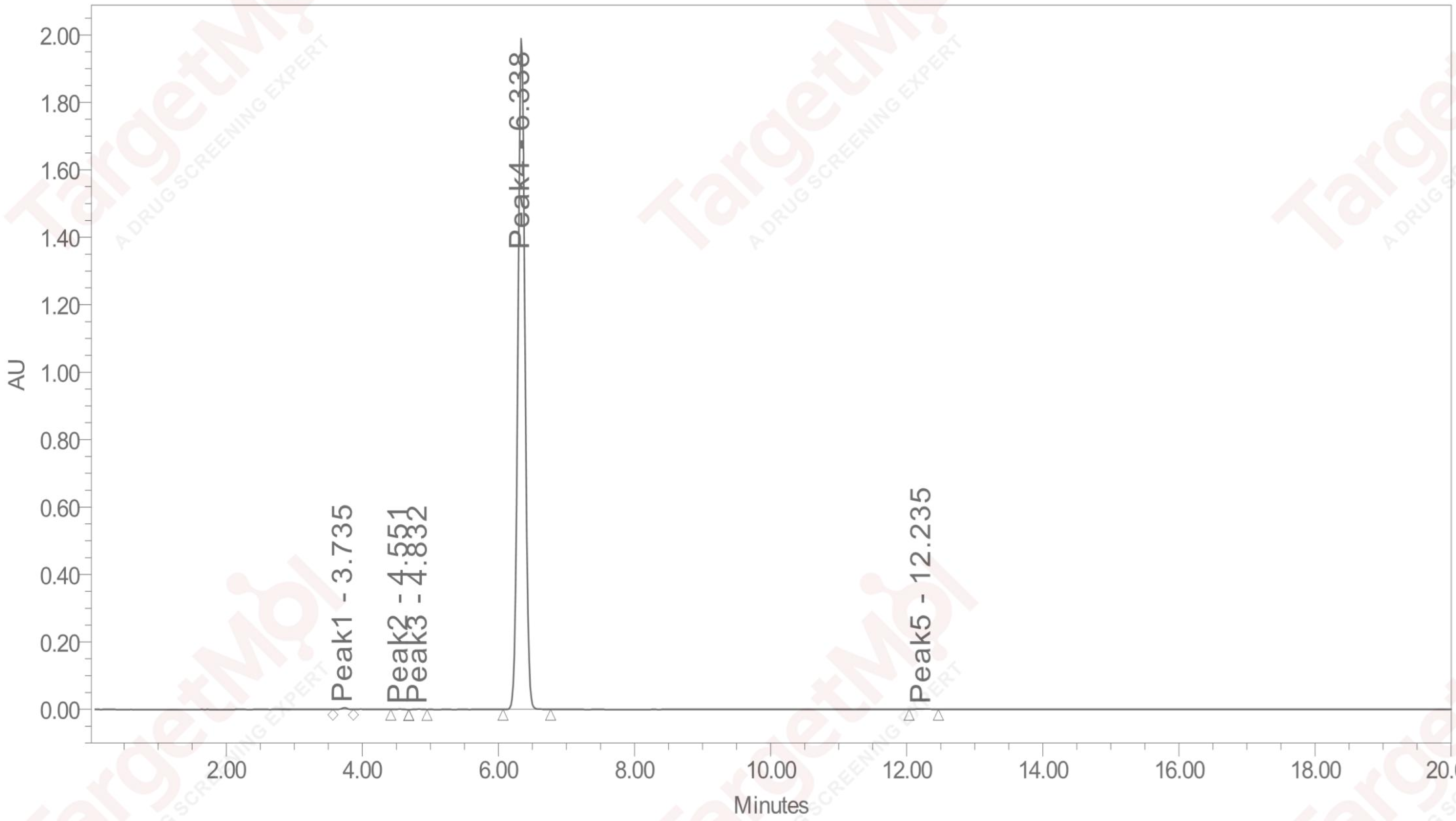

	Peak Name	RT	Area	% Area	Height
1	Peak1	3.735	27776	0.20	4848
2	Peak2	4.551	5290	0.04	903
3	Peak3	4.832	5168	0.04	769
4	Peak4	6.338	14193502	99.65	1988307
5	Peak5	12.235	11142	0.08	941
