# Supplementary material for: Identification of CB1 Ligands among Drugs, Phytochemicals and Natural-Like Compounds: Virtual Screening and In Vitro Verification
Source: ACS Chem Neurosci. 2022 Oct 5;13(20):2991–3007. doi: 10.1021/acschemneuro.2c00502 (PMC9585589; doi:10.1021/acschemneuro.2c00502)
Supplement: Supplementary file 3 — cn2c00502_si_003.zip [file cn2c00502_si_003.zip › Purity_identity_files/Second iteration/Molport/T5858_140606_HPLC.pdf]

Tgt Mass(EZX) :

Sample Name : 140606
Acq. Operator : 5
Spec. Reported : Integration

Location : P1-C-03
Inj : 0
Inj Volume : 5 ul

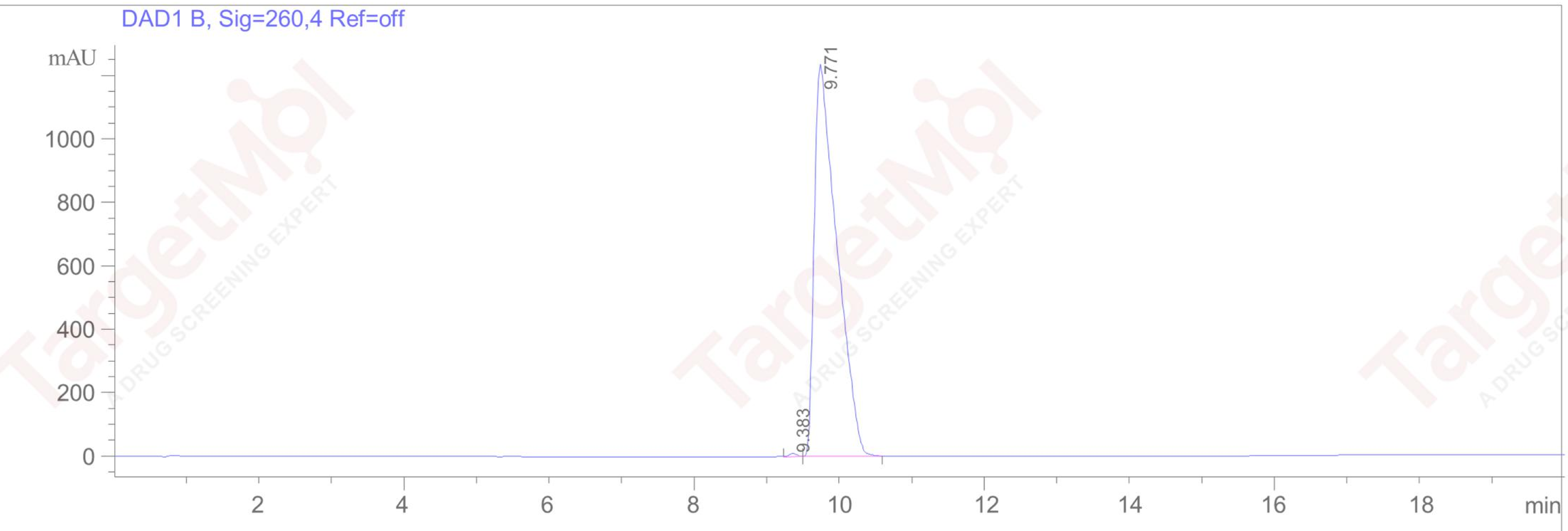

Integration Results for DAD1 B, Sig=260,4 Ref=off

RetTim	Width	Area	Height	Area%
9.38	0.05	35.15	10.80	0.26
9.77	0.25	13389.46	1236.46	99.74

TargetMol A DRUG SCREENING EXPERT
