# Supplementary material for: Identification of CB1 Ligands among Drugs, Phytochemicals and Natural-Like Compounds: Virtual Screening and In Vitro Verification
Source: ACS Chem Neurosci. 2022 Oct 5;13(20):2991–3007. doi: 10.1021/acschemneuro.2c00502 (PMC9585589; doi:10.1021/acschemneuro.2c00502)
Supplement: Supplementary file 3 — cn2c00502_si_003.zip [file cn2c00502_si_003.zip › Purity_identity_files/Second iteration/Molport/T6453_145439_HPLC.pdf]

Area Percent Report

Agilent Technologies

Data file: E:\HPLC\data\TEST\0CJK-2 10-28 16-40-44\007-P2-C7-14543917-40-45.D
Sample name: 145439

**Instrument:**	2DLC	**Sample type:**	Sample
**Acq. method:**	F0.6 285nm.M	**Location:**	P2-C7
**Analysis method:**	F0.6 285nm.M	**Injection:**	1 of 1
		**Injection volume:**	0.500

FLOW RATE : 0.6ml/min
COLUNM : ACE C18 50*2.1 2um
COLUNM TEMPERATURE : 30
GRADIENT PROGRAME :

TIME(MIN)	MOBILE PHASE-A(H2O+0.1TFA)	MOBILE PHASE-B(CH3CN+0.1TFA)
0	90%	10%
6	0%	100%
8	0%	100%

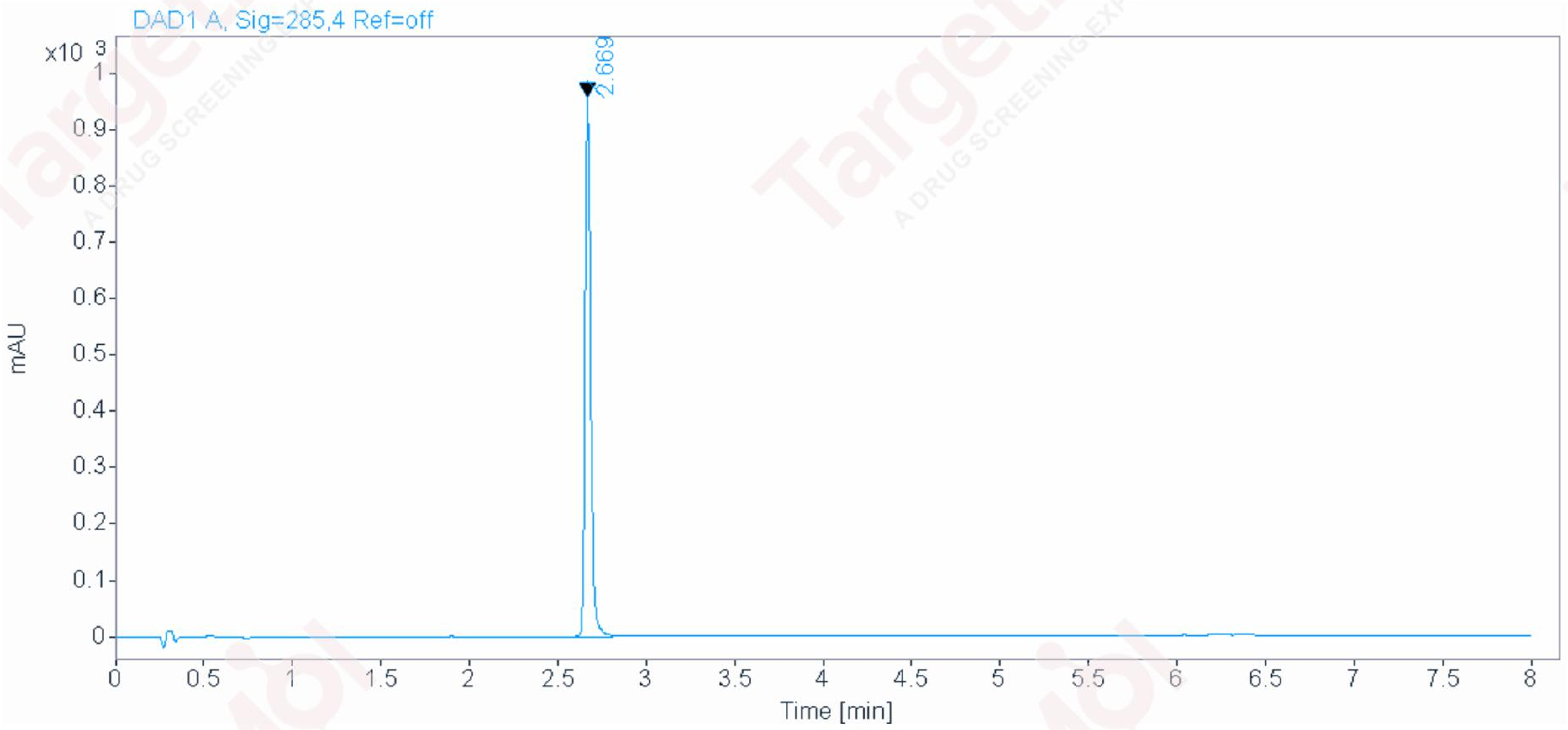

Signal: DAD1 A, Sig=285,4 Ref=off

RT [min]	Type	Width [min]	Area	Height	Area%	Name
2.669	BB	0.0359	2128.7493	957.6265	100.0000	
		Sum	2128.7493			

TargetMol A DRUG SCREENING EXPERT
