# Supplementary material for: Identification of CB1 Ligands among Drugs, Phytochemicals and Natural-Like Compounds: Virtual Screening and In Vitro Verification
Source: ACS Chem Neurosci. 2022 Oct 5;13(20):2991–3007. doi: 10.1021/acschemneuro.2c00502 (PMC9585589; doi:10.1021/acschemneuro.2c00502)
Supplement: Supplementary file 3 — cn2c00502_si_003.zip [file cn2c00502_si_003.zip › Purity_identity_files/Second iteration/Molport/V003-5572.PDF]

Sample: 128
File: Ar33058a_28
Vial: D/4

Date: 03-Apr-2010
Time: 01:10:14
Description: 10649881

AMRI code: ALB-H01831608
Vial label: M1770762ACC0049

(2) ELSD Signal

max. intensity: 2E4

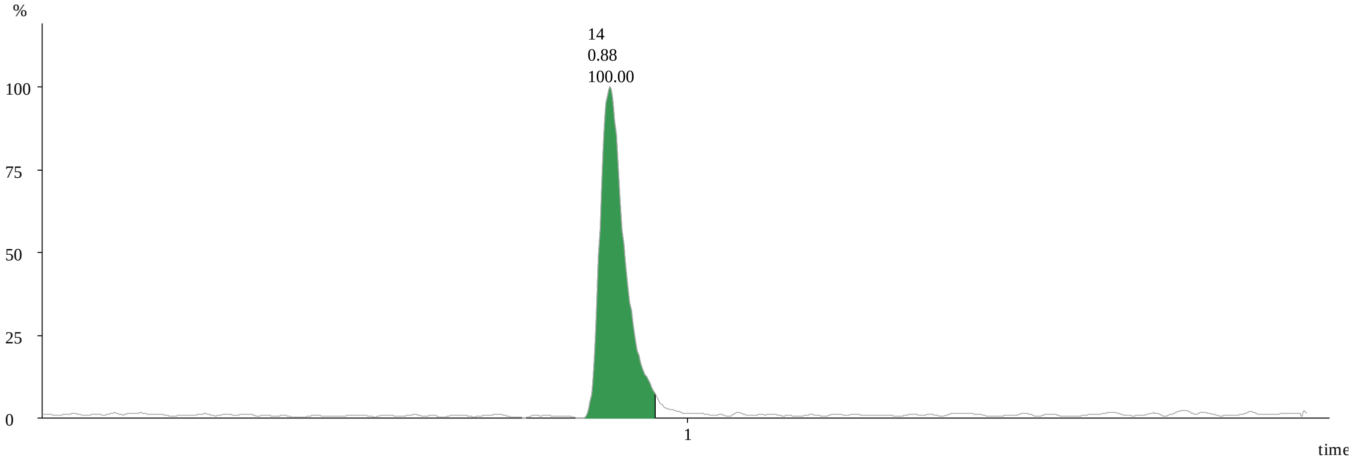

Peak_ID	Peak	Area	Area%	Height	Time	Mass Found
14	0.84 0.95	8.E2	100	2.E4	0.88	440.23

DAD: 220

max. intensity: 1.1E6

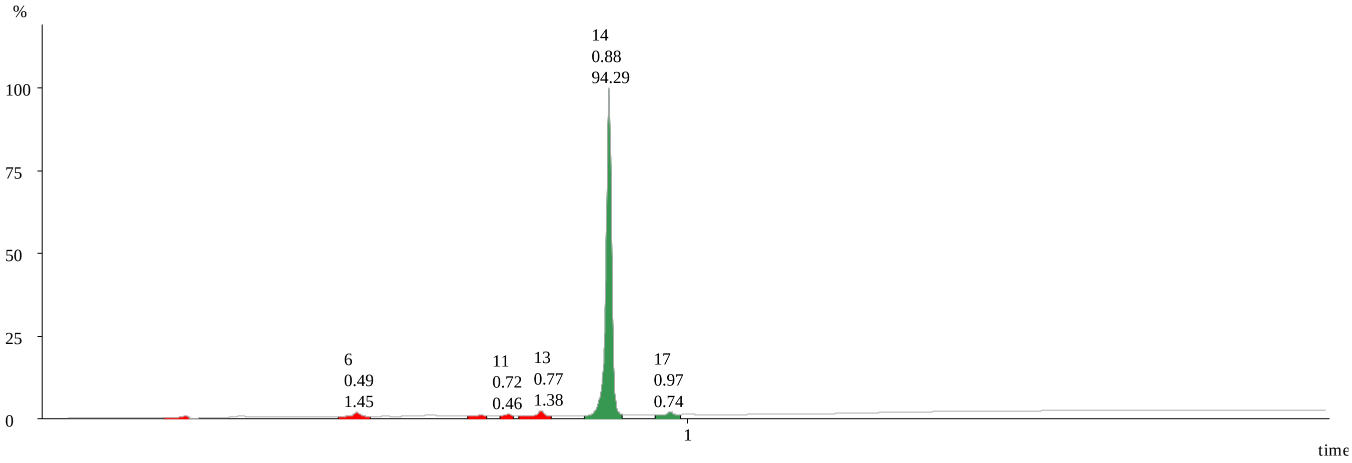

Peak_ID	Peak	Area	Area%	Height	Time	Mass Found
1	0.19 0.23	2.E2	1.28	9.E3	0.22	
6	0.46 0.51	2.E2	1.45	1.E4	0.49	
9	0.66 0.69	5.E1	0.4	5.E3	0.68	
11	0.71 0.73	6.E1	0.46	6.E3	0.72	
13	0.74 0.79	2.E2	1.38	2.E4	0.77	
14	0.84 0.90	1.E4	94.29	1.E6	0.88	440.23
17	0.95 0.99	9.E1	0.74	9.E3	0.97	440.23

File: Ar33058a_28
Time: 01:10:14
AMRI code: ALB-H01831608
Vial: D/4
Description: 10649881
Vial label: M1770762ACC0049

MS AP+ :441.23

max. intensity: 9.7E5

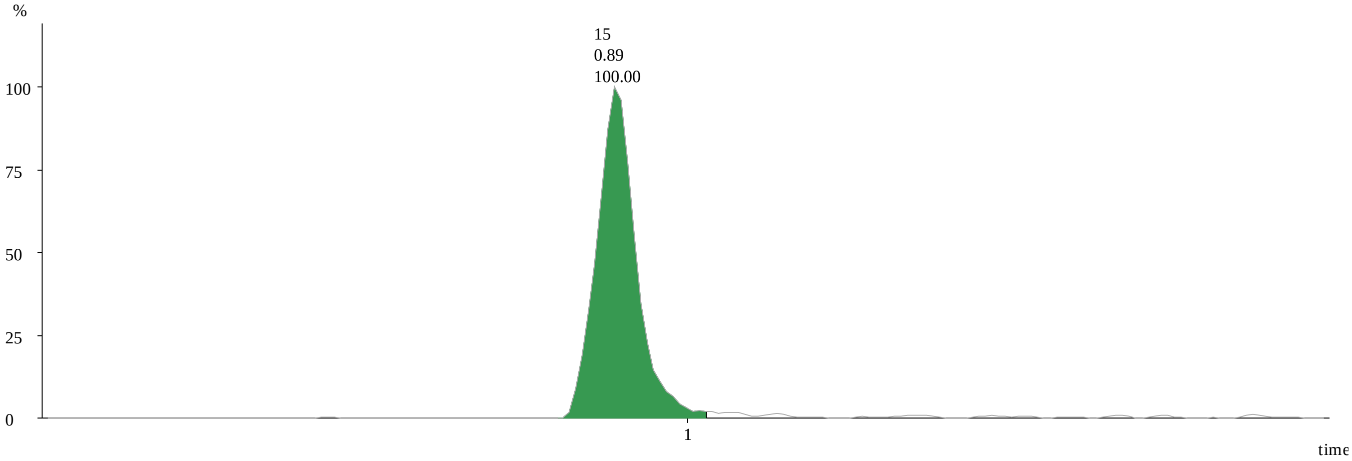

Peak_ID	Peak	Area	Area%	Height	Time	Mass Found
15	0.80 1.03	7.E4	100	1.E6	0.89	440.23

MS AP+ :TIC

max. intensity: 1.5E6

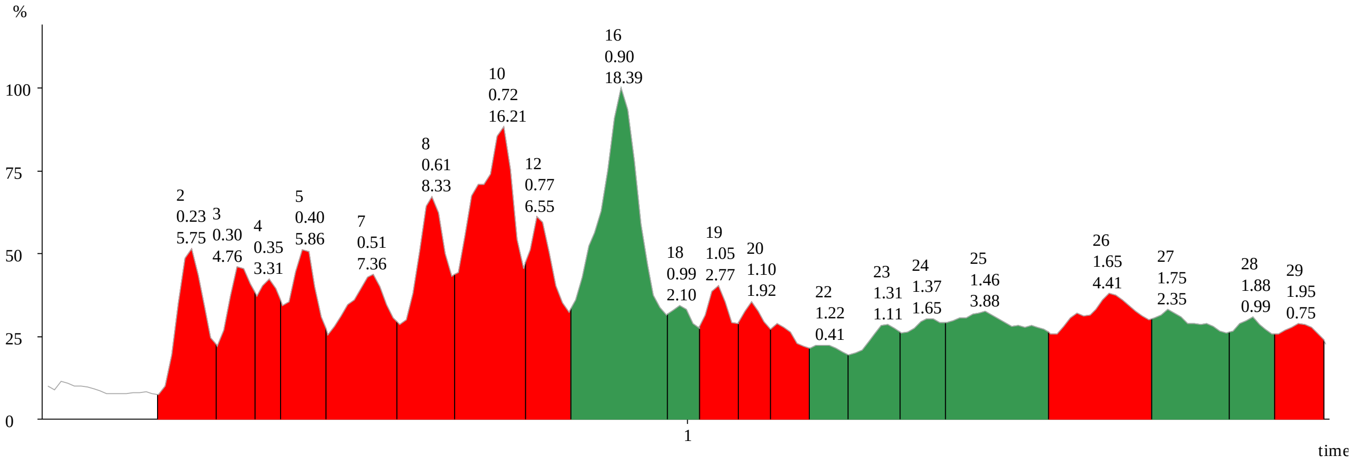

Peak_ID	Peak	Area	Area%	Height	Time	Mass Found
2	0.18 0.27	3.E4	5.75	7.E5	0.23	
3	0.27 0.33	3.E4	4.76	6.E5	0.30	
4	0.33 0.37	2.E4	3.31	5.E5	0.35	
5	0.37 0.44	3.E4	5.86	6.E5	0.40	
7	0.44 0.55	4.E4	7.36	5.E5	0.51	
8	0.55 0.64	5.E4	8.33	8.E5	0.61	
10	0.64 0.75	9.E4	16.21	1.E6	0.72	
12	0.75 0.82	4.E4	6.55	7.E5	0.77	
16	0.82 0.97	1.E5	18.39	1.E6	0.90	440.23
18	0.97 1.02	1.E4	2.1	3.E5	0.99	440.23
19	1.02 1.08	2.E4	2.77	4.E5	1.05	
20	1.08 1.13	1.E4	1.92	3.E5	1.10	
21	1.13 1.19	6.E3	1.15	2.E5	1.14	
22	1.19 1.25	2.E3	0.41	5.E4	1.22	440.23
23	1.25 1.33	6.E3	1.11	1.E5	1.31	440.23
24	1.33 1.40	9.E3	1.65	2.E5	1.37	440.23
25	1.40 1.56	2.E4	3.88	2.E5	1.46	440.23

26	1.56 1.72	2.E4	4.41	2.E5	1.65	
27	1.72 1.84	1.E4	2.35	2.E5	1.75	440.23
28	1.84 1.91	6.E3	0.99	1.E5	1.88	440.23
29	1.91 1.99	4.E3	0.75	8.E4	1.95	

MS: AP+

Combine (20:23-(14:16+28:30))

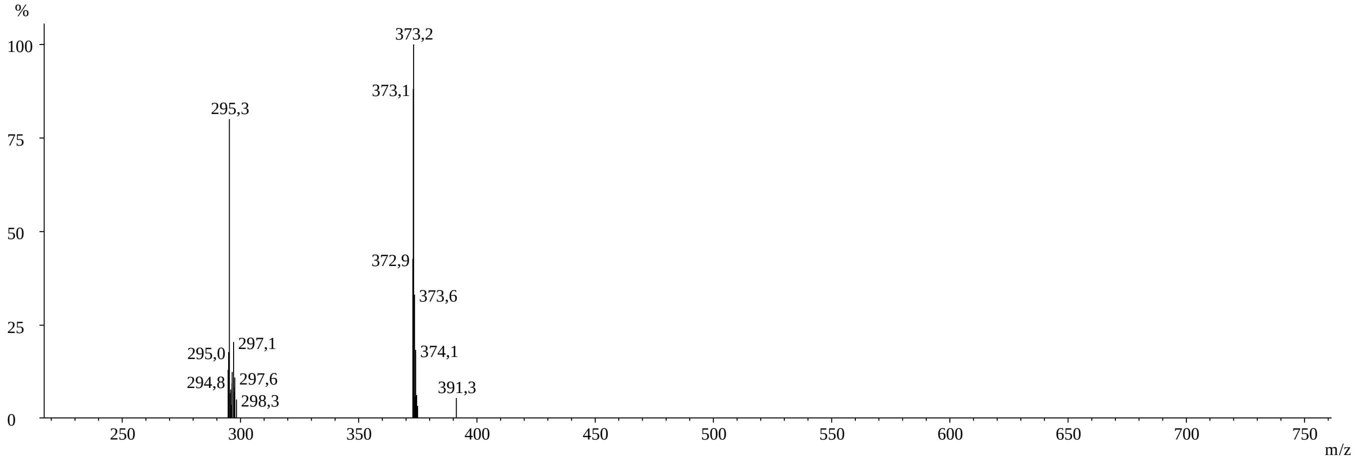

Peak_ID	Compound	Time	Mass found
1		0.22	

MS: AP+

Combine (47:50-(40:42+55:57))

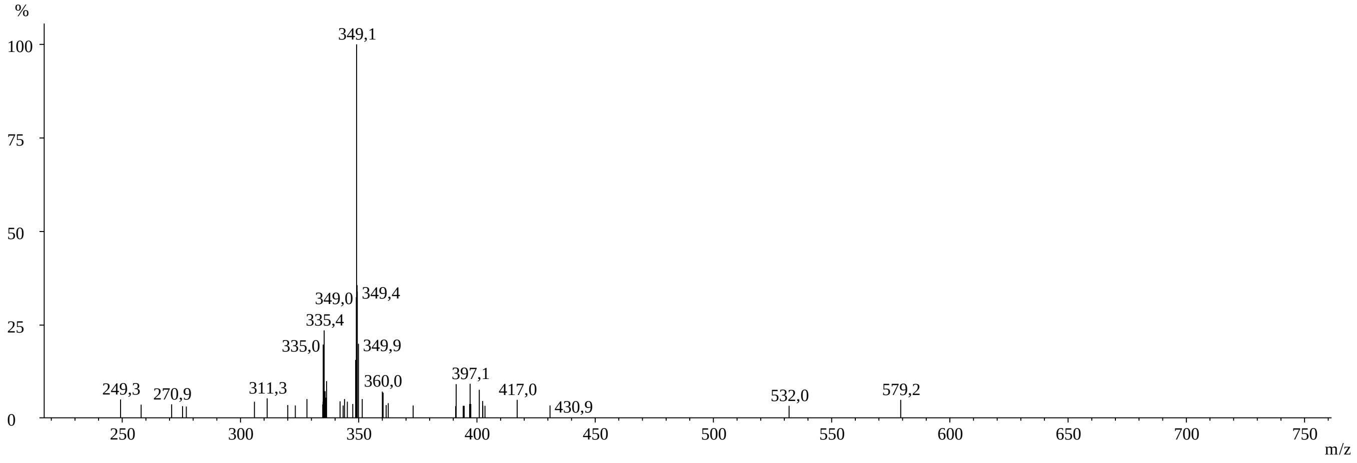

Peak_ID	Compound	Time	Mass found
6		0.49	

File: Ar33058a_28 Time: 01:10:14 AMRI code: ALB-H01831608
Vial: D/4 Description: 10649881 Vial label: M1770762ACC0049

MS: AP+

Combine (75:78-(68:70+83:85))

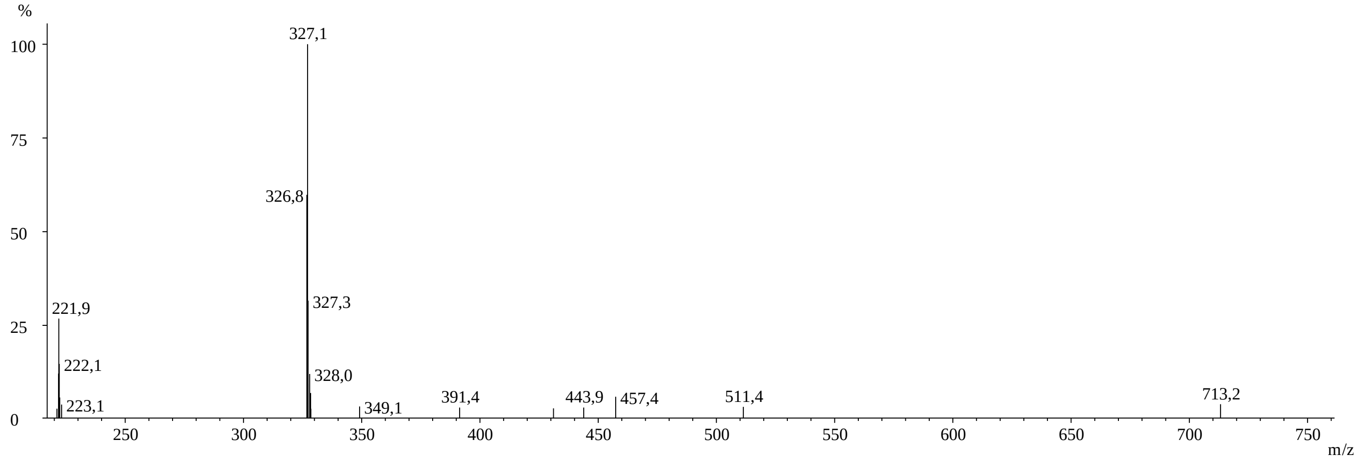

Peak_ID	Compound	Time	Mass found
13		0.77	

MS: AP+

Combine (86:89-(79:81+94:96))

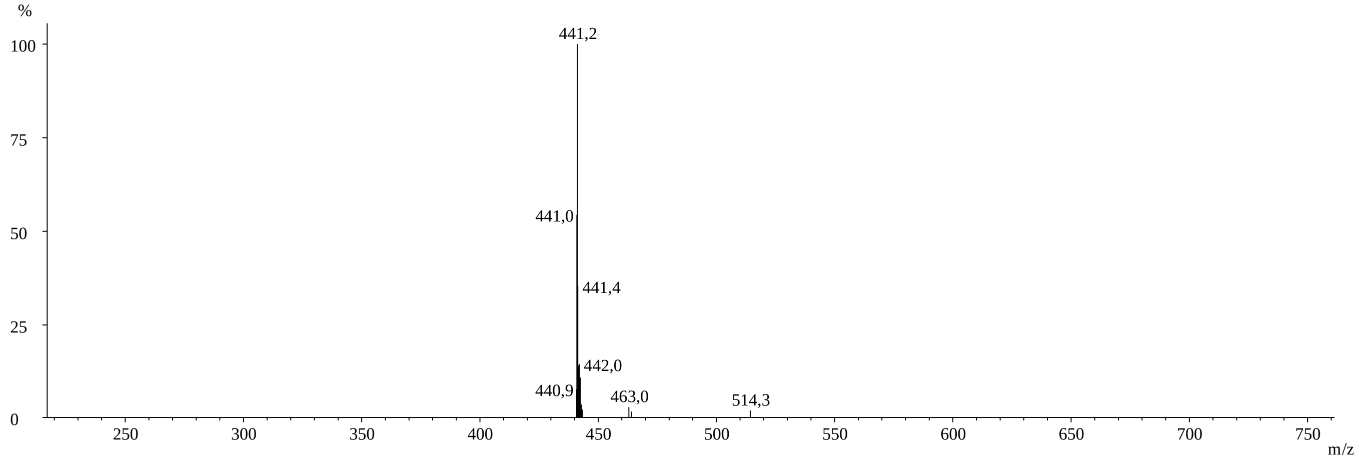

Peak_ID	Compound	Time	Mass found
14	Found	0.88	440.2300
