# Supplementary figures and images for: Identification of CB1 Ligands among Drugs, Phytochemicals and Natural-Like Compounds: Virtual Screening and In Vitro Verification
Source: ACS Chem Neurosci. 2022 Oct 5;13(20):2991–3007. doi: 10.1021/acschemneuro.2c00502 (PMC9585589; doi:10.1021/acschemneuro.2c00502)

CFN92320

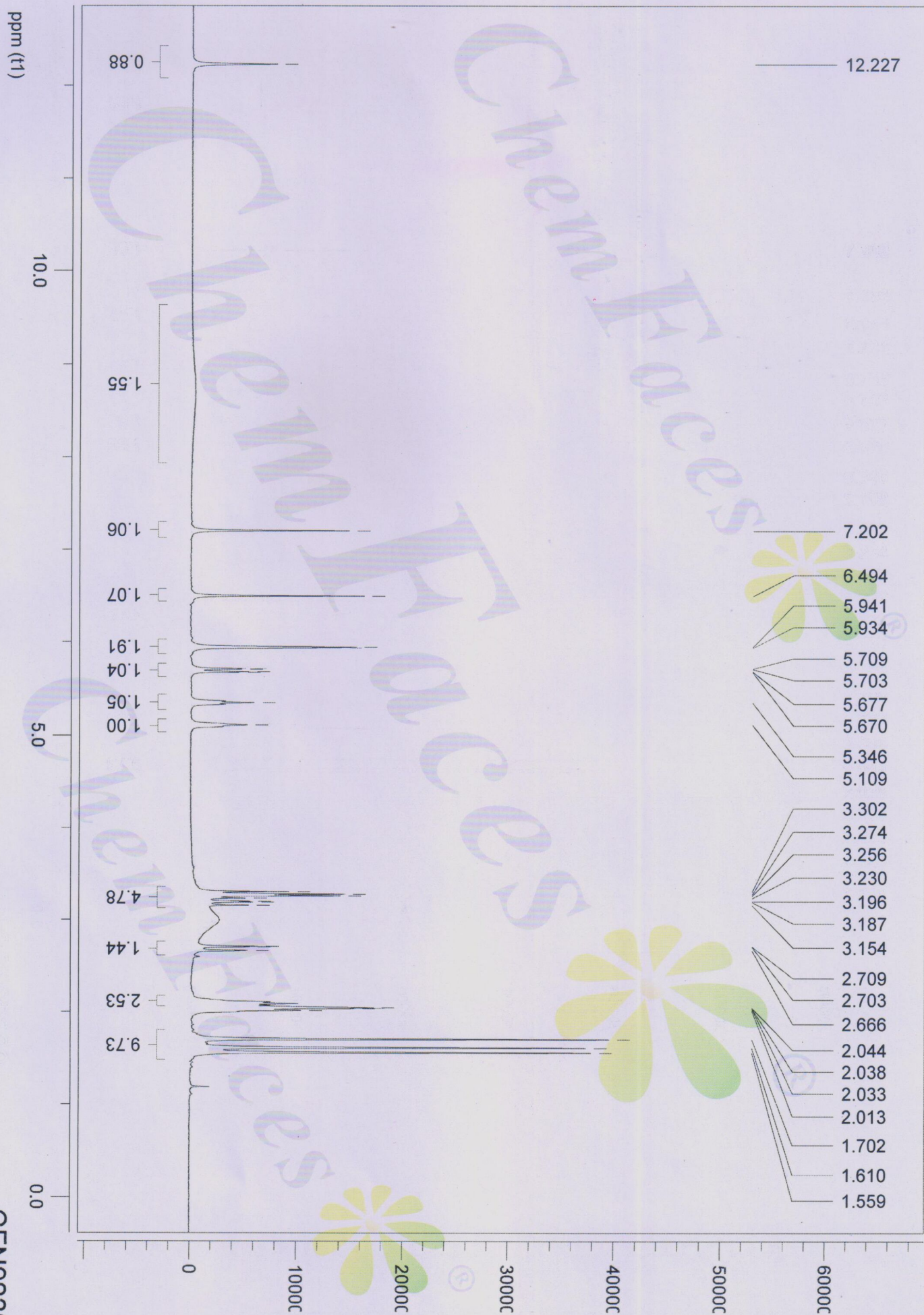

Supplement: Supplementary file 3 — cn2c00502_si_003.zip [file cn2c00502_si_003.zip › Purity_identity_files/Second iteration/ChemSpace/CFN92320 NMR.pdf]

CFN97979

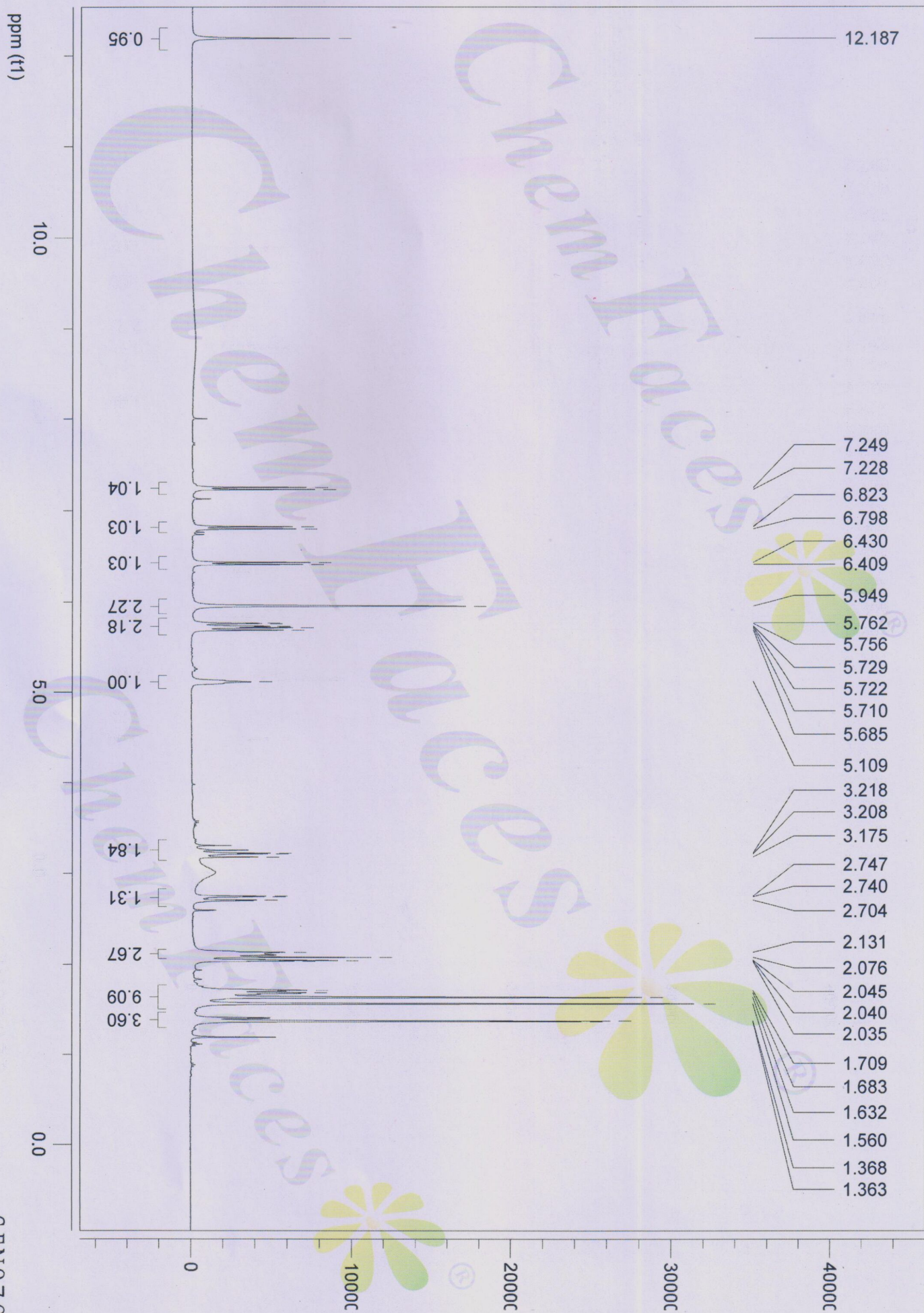

Supplement: Supplementary file 3 — cn2c00502_si_003.zip [file cn2c00502_si_003.zip › Purity_identity_files/Second iteration/ChemSpace/CFN97979 NMR.pdf]

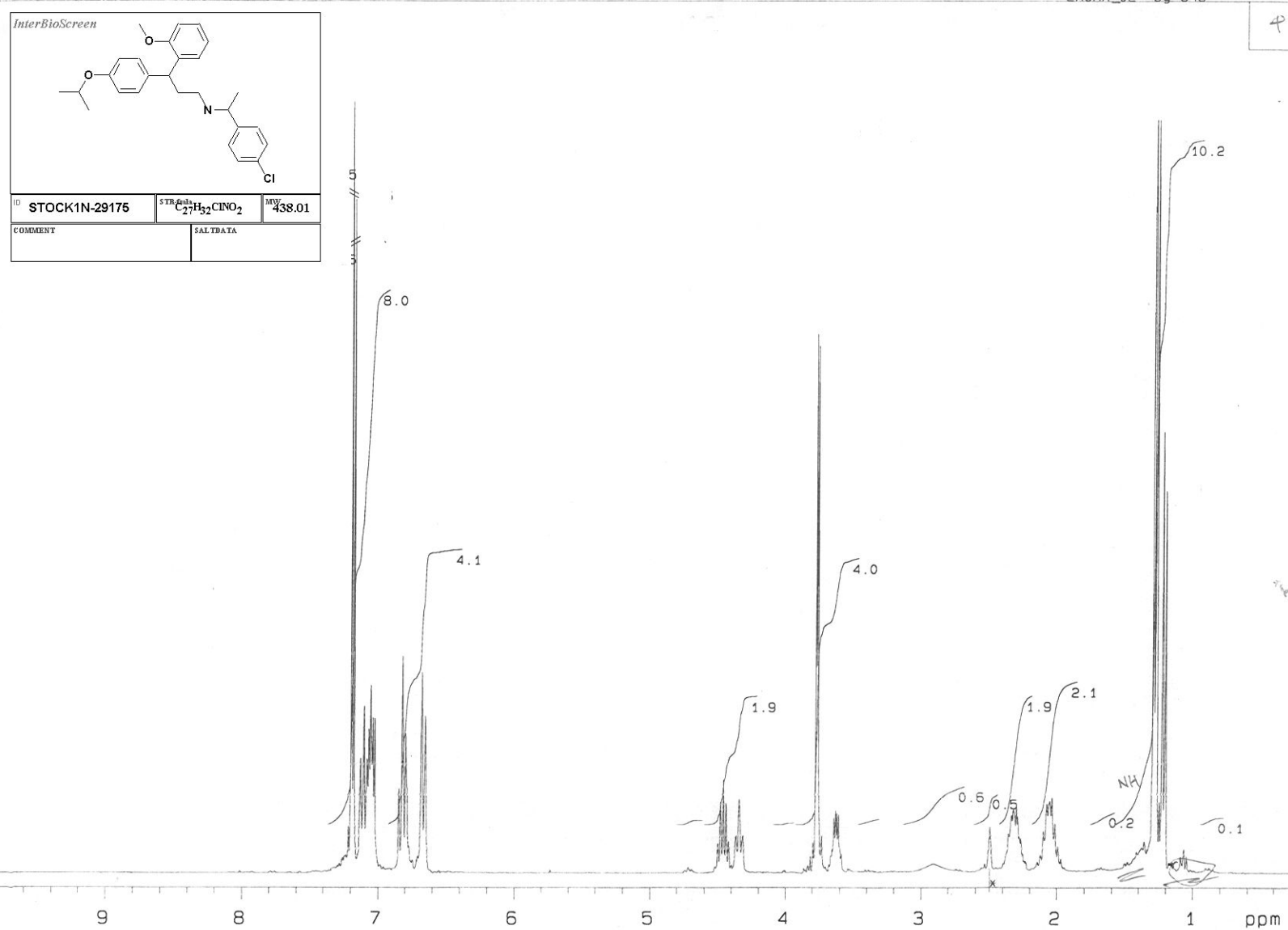

Supplement: Supplementary file 3 — cn2c00502_si_003.zip [file cn2c00502_si_003.zip › Purity_identity_files/Second iteration/Molport/Spectra_IBScreen/STOCK1N-29175.pdf]

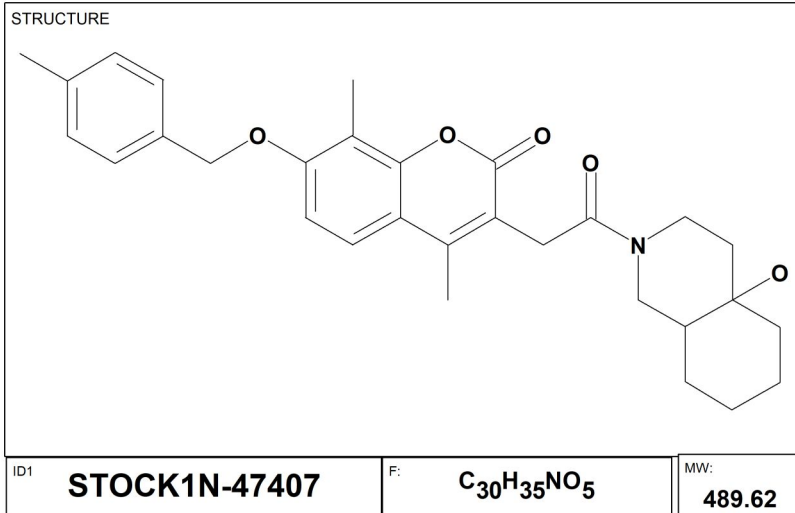

Com: Saltdata: ID1 **NC\_0075-4302**

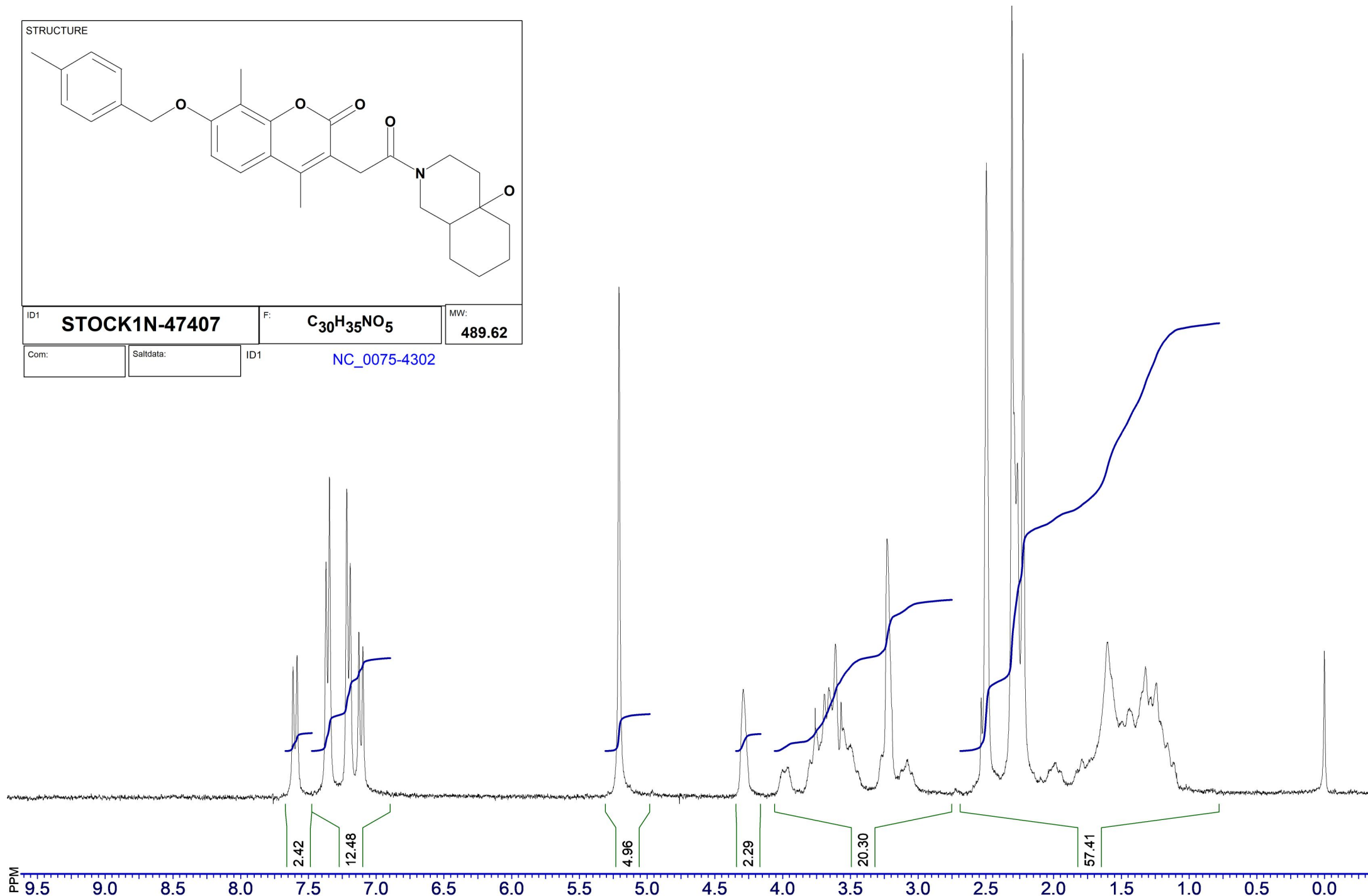

|                             |          |                  |        |                      |
|-----------------------------|----------|------------------|--------|----------------------|
| File name: NC-0075-4302.nmr | Owner:   | SF: 299.9450 MHz | NS: -1 | SI: 16384, TD: 32768 |
| Date: 30-Dec-1899           | Solvent: | SW: 5099         | TE: 0  | M 74 in DMSO-D6      |

Supplement: Supplementary file 3 — cn2c00502_si_003.zip [file cn2c00502_si_003.zip › Purity_identity_files/Second iteration/Molport/Spectra_IBScreen/STOCK1N-47407.pdf]

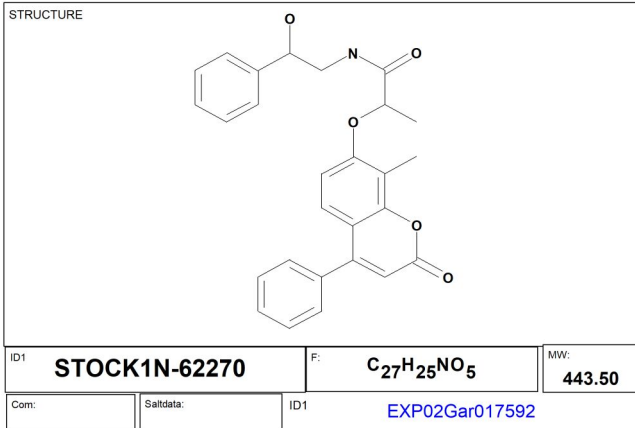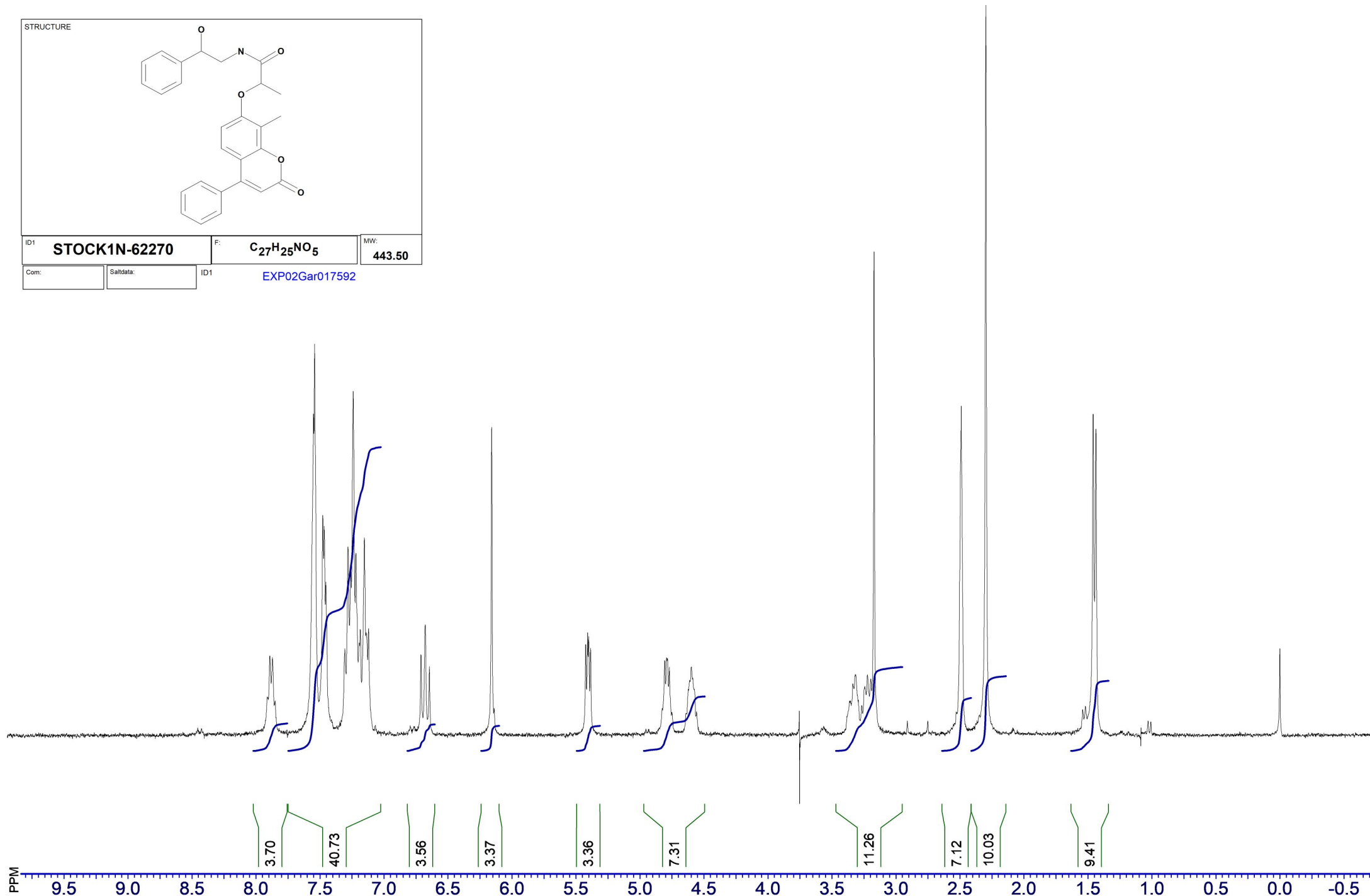

|                                  |          |                  |        |                      |
|----------------------------------|----------|------------------|--------|----------------------|
| File name: <b>EXP02Gar017592</b> | Owner:   | SF: 299.9450 MHz | NS: -1 | SI: 16384, TD: 32768 |
| Date:                            | Solvent: | SW: 5099         | TE: 0  | A-725 in DMSO-D6     |

Supplement: Supplementary file 3 — cn2c00502_si_003.zip [file cn2c00502_si_003.zip › Purity_identity_files/Second iteration/Molport/Spectra_IBScreen/STOCK1N-62270.pdf]

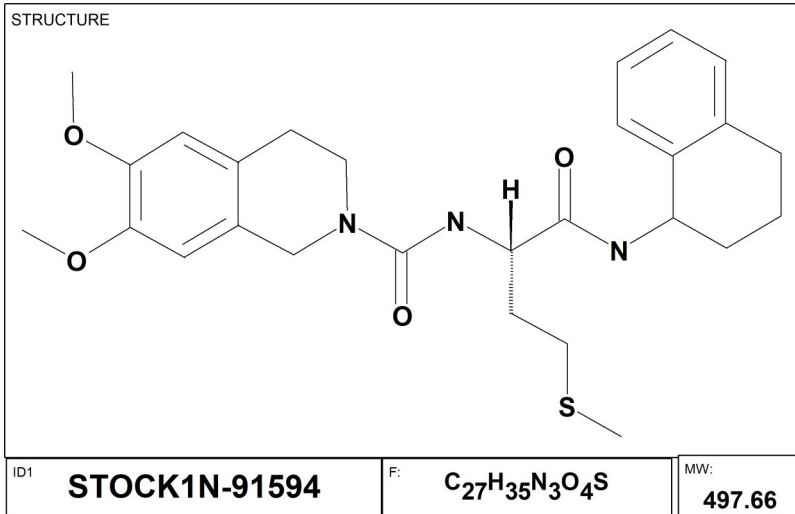

Com: Saltdata: ID1 [ExLab-005740](#)

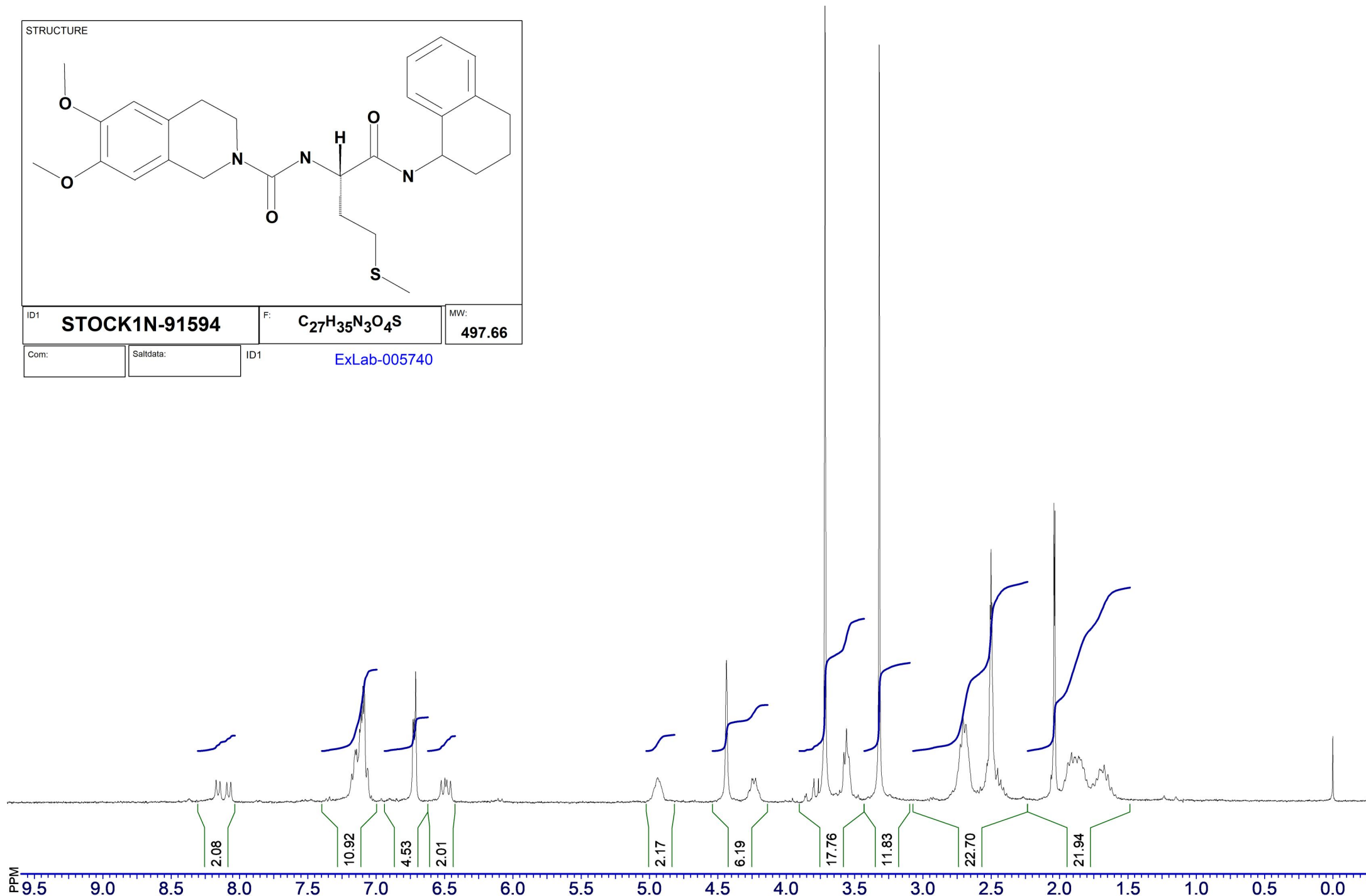

File name: EXLab-005740.nmr

Owner:

SF: 299.9450 MHz

NS: -1

SI: 16384, TD: 32768

Date: 30-Dec-1899

Solvent:

SW: 5099

TE: 0

N 1780 IN DMSO-D6

Supplement: Supplementary file 3 — cn2c00502_si_003.zip [file cn2c00502_si_003.zip › Purity_identity_files/Second iteration/Molport/Spectra_IBScreen/STOCK1N-91594.pdf]

<sup>1</sup>H NMR (400 MHz, DMSO-*d*<sub>6</sub>) δ ppm 0.96 (t, *J*=7.40 Hz, 3 H) 1.74 (tddd, *J*=7.58, 7.58, 7.55, 7.49, 7.46 Hz, 2 H) 3.05 - 3.22 (m, 2 H) 7.23 - 7.34 (m, 1 H) 7.49 - 7.67 (m, 3 H) 7.72 - 7.88 (m, 2 H) 8.25 (s, 1 H) 8.64 (br. s., 1 H) 8.72 (d, *J*=2.20 Hz, 1 H) 9.77 (br. s., 1 H) 13.02 (br. s., 1 H)

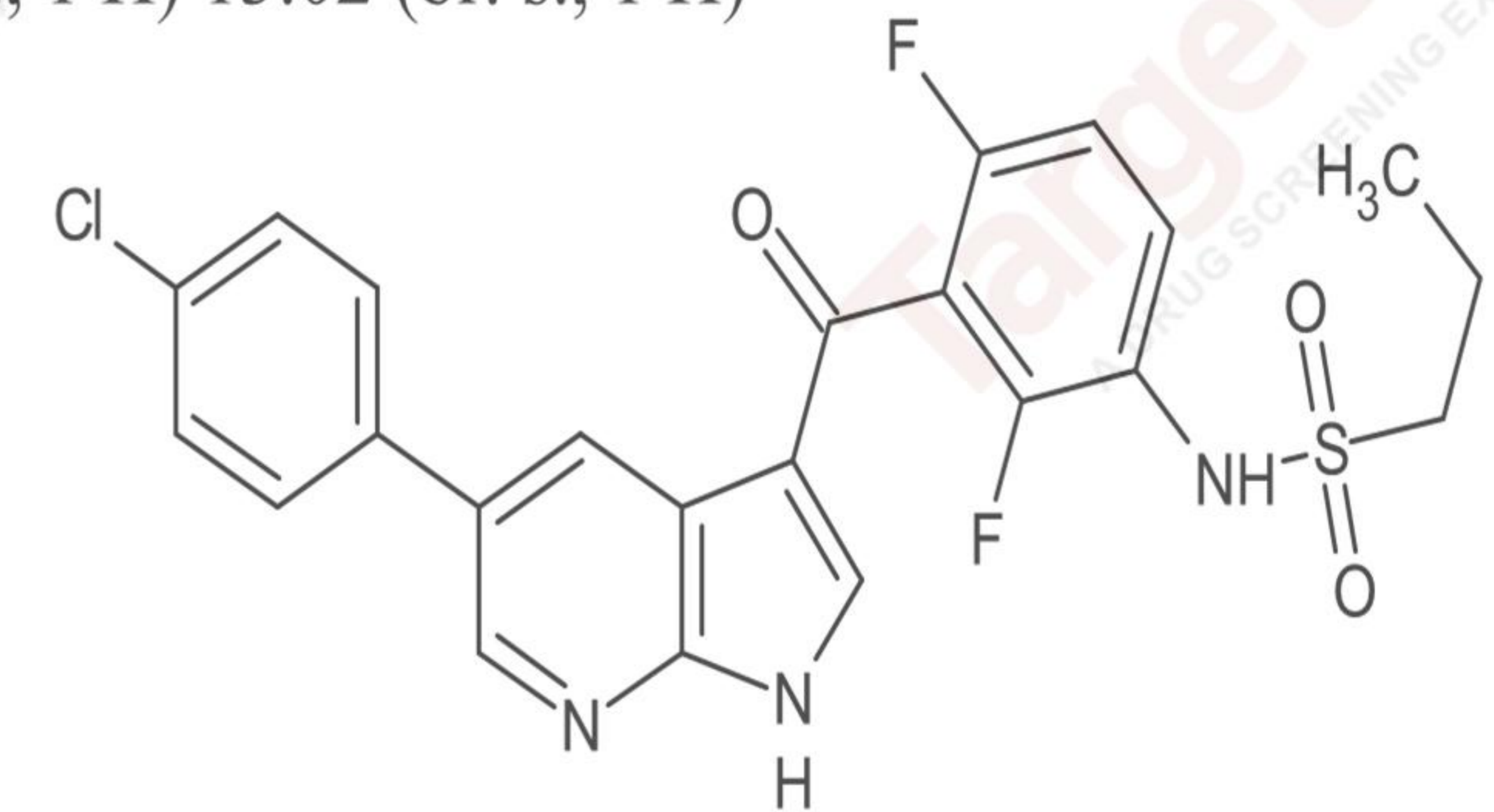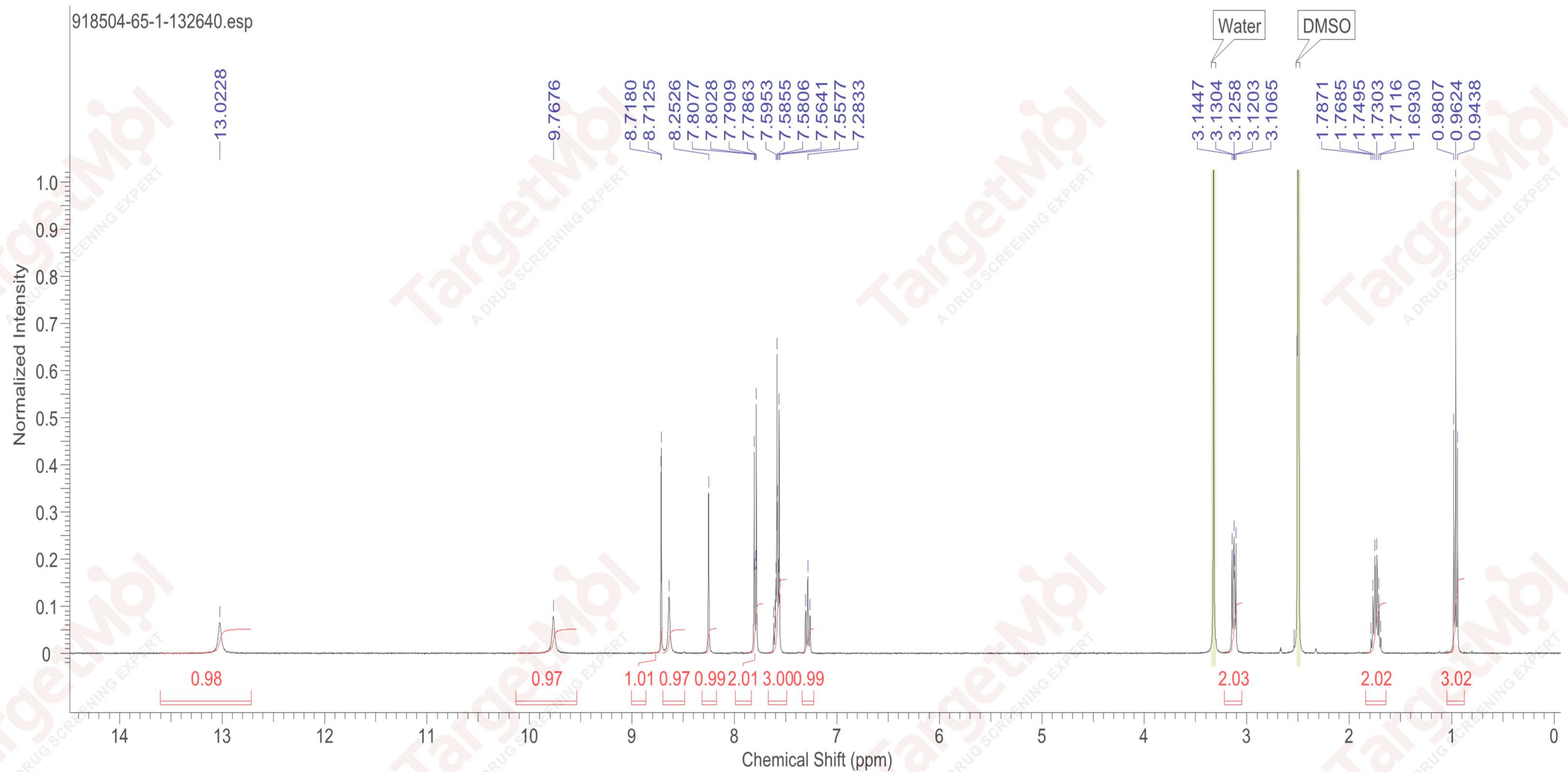

Supplement: Supplementary file 3 — cn2c00502_si_003.zip [file cn2c00502_si_003.zip › Purity_identity_files/Second iteration/Molport/T2382_132640_HNMR.pdf]

$^1\text{H}$  NMR (400 MHz,  $\text{CHCl}_3$ )  $\delta$  ppm 2.16 (d,  $J=15.89$  Hz, 4 H) 2.51 (br. s., 2 H) 2.88 (d,  $J=28.00$  Hz, 3 H) 3.17 - 3.86 (m, 8 H) 4.40 (br. s., 1 H) 7.12 (s, 1 H) 7.16 (dd,  $J=8.80, 1.96$  Hz, 1 H) 7.18 - 7.25 (m, 2 H) 7.34 (d,  $J=8.80$  Hz, 1 H) 7.37 - 7.49 (m, 2 H) 7.62 (d,  $J=1.83$  Hz, 1 H)

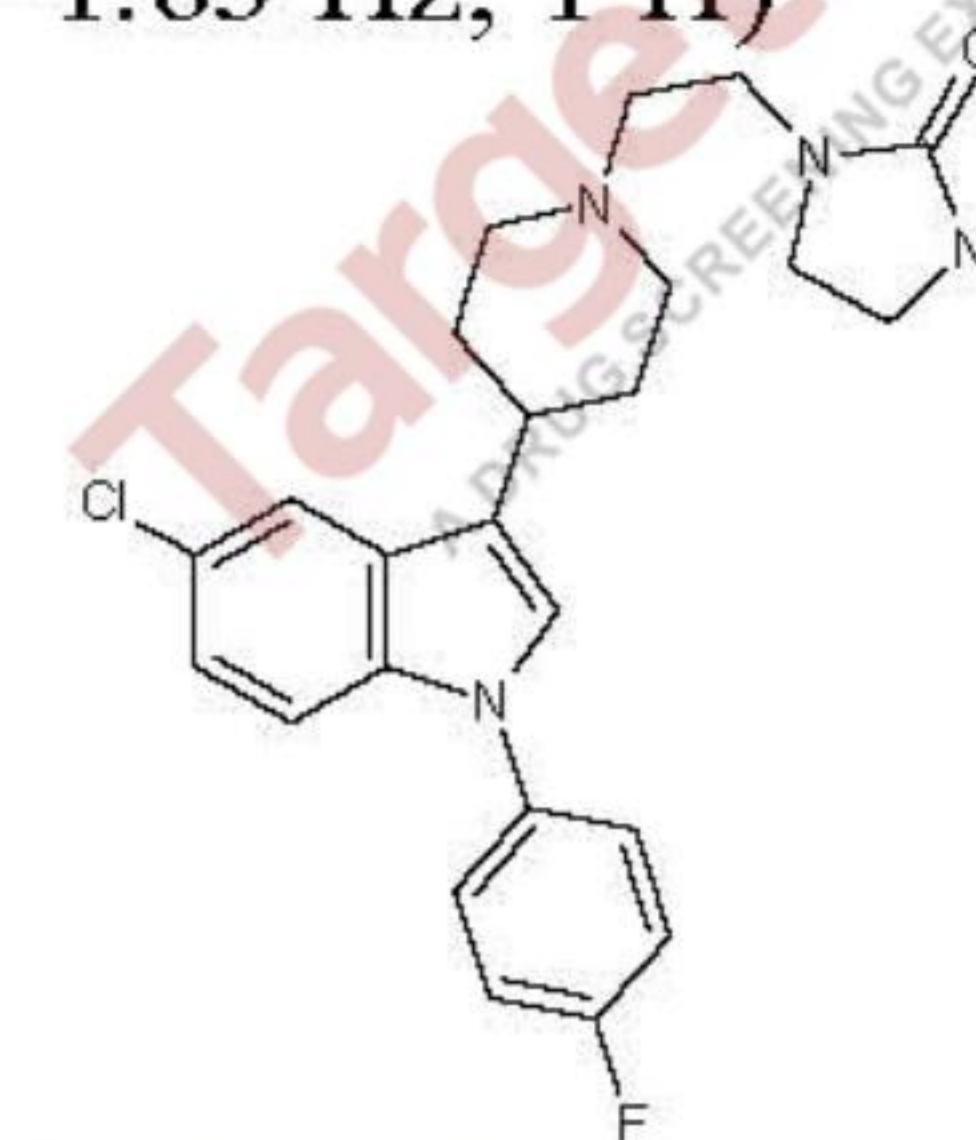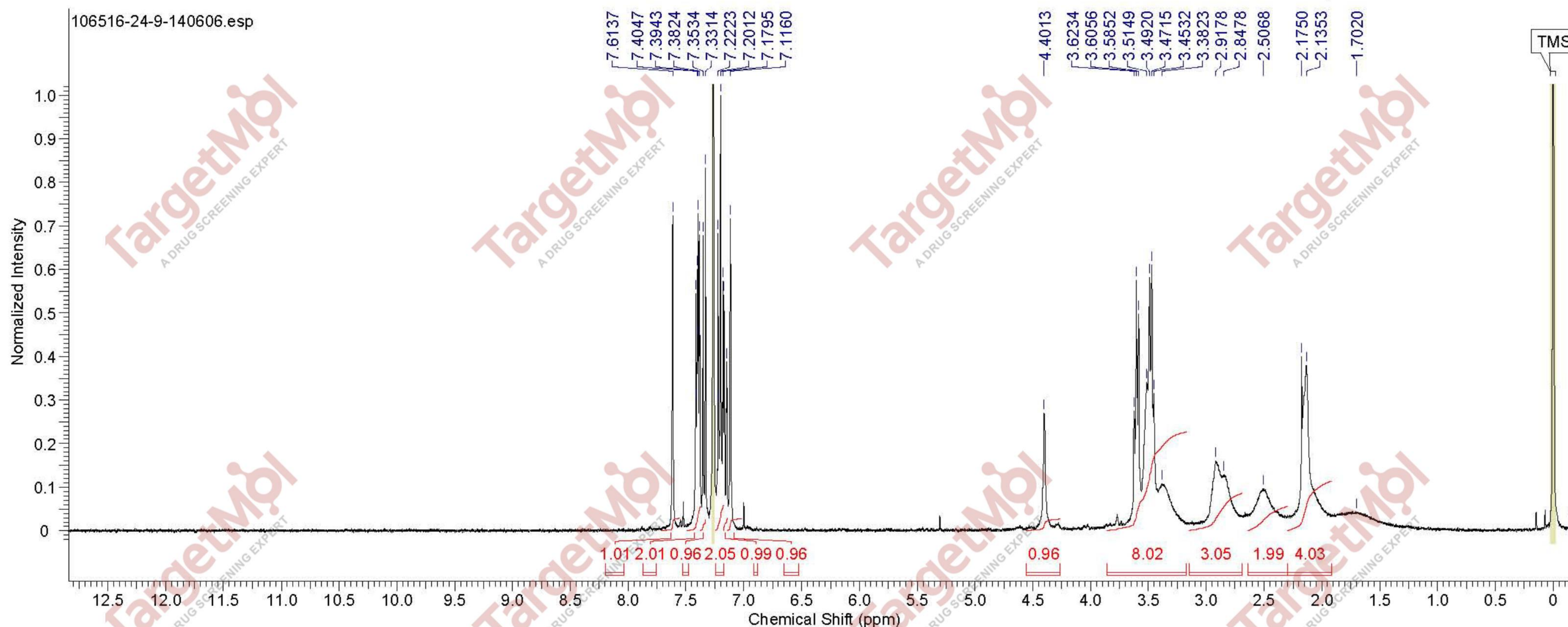

Supplement: Supplementary file 3 — cn2c00502_si_003.zip [file cn2c00502_si_003.zip › Purity_identity_files/Second iteration/Molport/T5858_140606_HNMR.pdf]
